# Selective footprints and genes relevant to cold adaptation and other phenotypic traits are unscrambled in the genomes of divergently selected chicken breeds

**DOI:** 10.1186/s40104-022-00813-0

**Published:** 2023-02-24

**Authors:** Michael N. Romanov, Alexandra S. Abdelmanova, Vladimir I. Fisinin, Elena A. Gladyr, Natalia A. Volkova, Olga A. Koshkina, Andrey N. Rodionov, Anastasia N. Vetokh, Igor V. Gusev, Dmitry V. Anshakov, Olga I. Stanishevskaya, Arsen V. Dotsev, Darren K. Griffin, Natalia A. Zinovieva

**Affiliations:** 1 L.K. Ernst Federal Research Centre for Animal Husbandry, Dubrovitsy, Podolsk, Moscow Region Russia; 2 School of Biosciences, University of Kent, Canterbury, UK; 3 Federal State Budget Scientific Institution Federal Research Centre “All-Russian Poultry Research and Technological Institute” of the Russian Academy of Sciences, Sergiev Posad, Moscow Region Russia; 4 Breeding and Genetic Centre “Zagorsk Experimental Breeding Farm” – Branch of the Federal Research Centre “All-Russian Poultry Research and Technological Institute” of the Russian Academy of Sciences, Sergiev Posad, Moscow Region Russia; 5 Russian Research Institute of Farm Animal Genetics and Breeding – Branch of the L.K. Ernst Federal Research Centre for Animal Husbandry, St. Petersburg, Russia

**Keywords:** Acclimation, Adaptation, Chicken breeds, Cold tolerance, Divergent selection, Genetic diversity, Genome-wide scan, Phenotypic traits, Selective sweeps

## Abstract

**Background:**

The genomes of worldwide poultry breeds divergently selected for performance and other phenotypic traits may also be affected by, and formed due to, past and current admixture events. Adaptation to diverse environments, including acclimation to harsh climatic conditions, has also left selection footprints in breed genomes.

**Results:**

Using the Chicken 50K_CobbCons SNP chip, we genotyped four divergently selected breeds: two aboriginal, cold tolerant Ushanka and Orloff Mille Fleur, one egg-type Russian White subjected to artificial selection for cold tolerance, and one meat-type White Cornish. Signals of selective sweeps were determined in the studied breeds using three methods: (1) assessment of runs of homozygosity islands, (2) *F*_ST_ based population differential analysis, and (3) haplotype differentiation analysis. Genomic regions of true selection signatures were identified by two or more methods or in two or more breeds. In these regions, we detected 540 prioritized candidate genes supplemented them with those that occurred in one breed using one statistic and were suggested in other studies. Amongst them, *SOX5*, *ME3*, *ZNF536*, *WWP1*, *RIPK2*, *OSGIN2*, *DECR1*, *TPO*, *PPARGC1A*, *BDNF*, *MSTN*, and beta-keratin genes can be especially mentioned as candidates for cold adaptation. Epigenetic factors may be involved in regulating some of these important genes (e.g., *TPO* and *BDNF*).

**Conclusion:**

Based on a genome-wide scan, our findings can help dissect the genetic architecture underlying various phenotypic traits in chicken breeds. These include genes representing the *sine qua non* for adaptation to harsh environments. Cold tolerance in acclimated chicken breeds may be developed following one of few specific gene expression mechanisms or more than one overlapping response known in cold-exposed individuals, and this warrants further investigation.

**Supplementary Information:**

The online version contains supplementary material available at 10.1186/s40104-022-00813-0.

## Background

Poultry is a traditional, important, integral and the fastest growing component of livestock agriculture. An estimated 70 billion chickens per year are raised and slaughtered for meat alone worldwide [1]. To increase the production of both eggs and meat, producers rely on developments in the field of biotechnology, classical genetics, and genomic research in both chicken (*Gallus gallus*; GGA) and other poultry species, thereby, improving and optimising breeding performance [2]. Diverse poultry breeds are adapted to local environments [3], formed in the course of past and recent admixture events [4] and divergently selected for a suite of phenotypic characters of interest. They can serve as valuable resources for peculiar genetic variants (e.g., [5, 6]; see also examples in Table S1), while further research of selective sweeps and underlying candidate genes in these breeds (e.g., [7–9]) is also possible and worthy of consequent breeding applications.

**Fig. 1:**
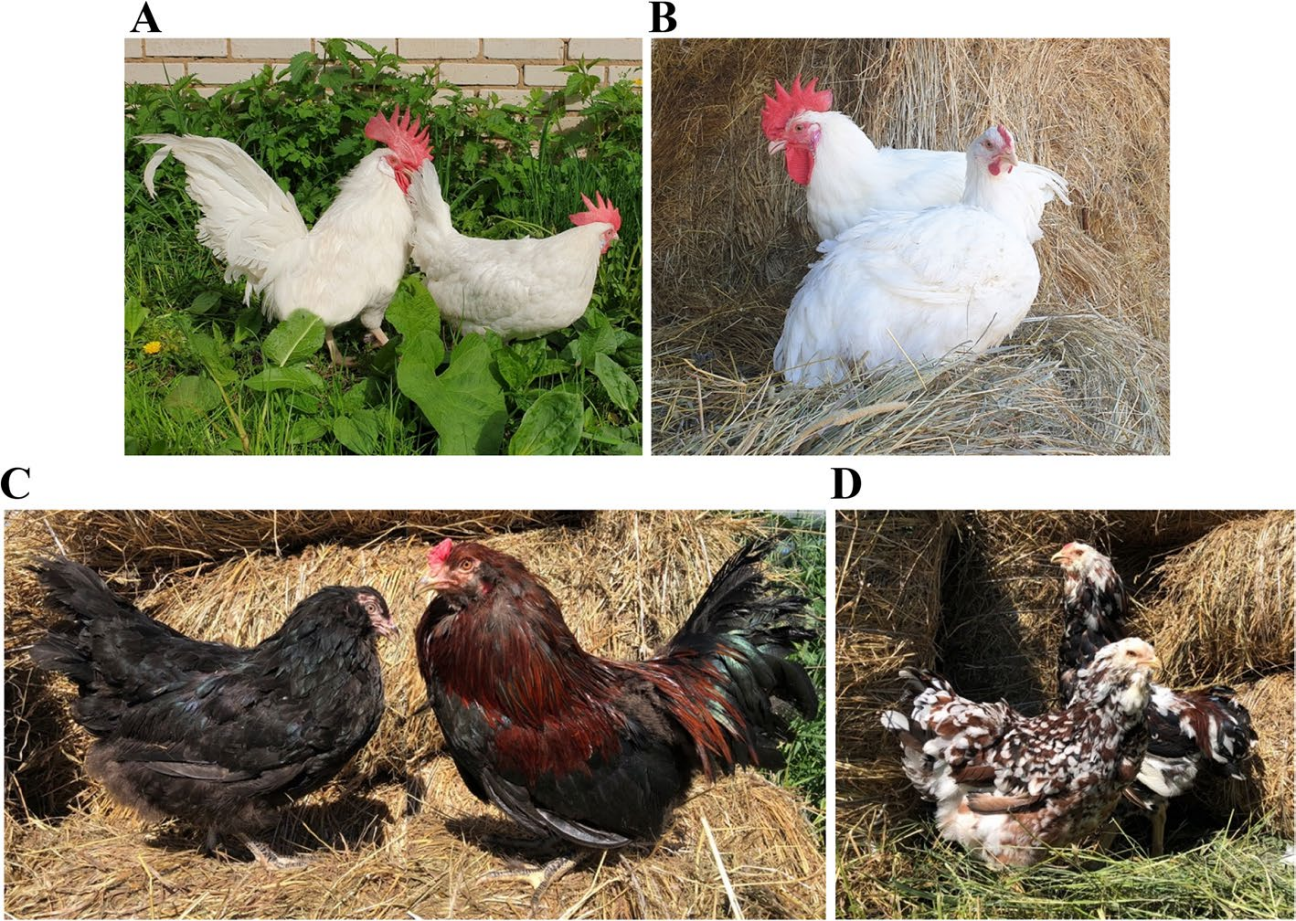
Four chicken breeds used in this study. **A** Russian White (male, left; female, right); **B** White Cornish (male, left; female, right); **C** Ushanka (female, left; male, right); and **D** Orloff Mille Fleur (female, left; male, right)

Recently, we carried out preliminary single nucleotide polymorphism (SNP)-based research in two breeds, the native egg-type Russian White (RUW) and meat-type White Cornish (WCR), using the Chicken 50K_CobbCons chip [10]. RUW (Fig. 1A) is one of the distinctive native egg-type breeds developed by crossing the White Leghorn and local Russian laying hens and bred for egg production in the former Soviet Union in 1929–1953 [10–12]. This breed is remarkable for carrying the classical genes [5, 13] for dominant white plumage and single comb (Table S1). WCR (Fig. 1B) is a typical meat-type breed broadly used as a male parent stock for broiler production [2, 14]. It is characterized by the classical genes for recessive white plumage and pea comb ([5]; Table S1). In the recent experiment [10], we genotyped 54 birds from these two breeds, estimated their genetic diversity and inbreeding, and unveiled footprints of artificial selection and related candidate genes associated with performance traits. In particular, we found significant SNPs and identified candidate genes for such traits as body temperature, egg performance and feed intake in RUW chickens, and body weight and feed efficiency in WCR chickens. Fedorova et al. [15] further attempted to identify key candidate genes in the RUW genome that could be associated with cold adaptation.

Among the native Russian breeds, of great interest is Ushanka (USH; Fig. 1C), one of the oldest native breeds, also known by names of the Russian Ushanka, South Russian Ushanka, and Ukrainian Muffed [16, 17]. The following description is available for USH ([13, 18]; see also the breed’s classical phenotype-relevant mutations in Table S1): this is an old native breed of unknown exact ancestry in the South of Russia, with its first description known since the 1880s. They are medium-sized, very hardy birds, not afraid of frost and well adapted to various climatic conditions. They have a rose comb and well-developed muffs and beard. Originally, they had black plumage in hens and black with a red hackle in roosters. Day-old chick down colour is black, although there is currently a wide variety of plumage coloration due to no special selection for this phenotypic trait. They have a creamy eggshell, predominantly black shank (caused by a wild type allele of the dermal melanin inhibitor) and retain a broody behaviour. In a recent study, Larkina et al. [19] assigned USH to dual purpose breeds.

Orloff Mille Fleur (OMF; Fig. 1D) belongs to the old Russian breeds and is also cold resistant [16, 20]. The following information was reported on OMF ([13, 18]; see also Table S1): it was widespread in the 18^th^ and 19^th^ centuries and believed to be created in Central Russia on the estate of Count Alexei Orlov Chesmensky (1737–1808) by crossing Malay-type game individuals with local bearded chickens [20]. They are distinguished by high vitality and unpretentiousness and tolerate both heat and severe frosts well. Due to late feathering, chicks require special care at raising, since they do not tolerate dampness and cold. They are traditionally used as a dual purpose breed and also for cock fighting. They are superbly selected for appearance and ornamental traits, much appreciated by poultry fanciers. Features include muffs and beard, walnut comb, fancy mille fleur plumage pattern, light beige eggshell colour, and light yellow chick down at one day old, with longitudinal striping of varying degrees on the back. They also have a preserved broodiness instinct. There were few reports suggesting that USH chickens were used as local bearded fowls at creating the Orloff breed [21]. Some other authors [20] also hypothesized that the breed stemmed from the Gilan breed (brought from the Gilan Province, Persia) or crested chickens were mated with Malay fowls to produce OMF.

It should be noted that the continental climate is dominant in a significant part of the territory of Russia and adjacent countries wherefrom USH, OMF and RUW originated. It is characterized by consistently cold winters, consistently hot summers and low rainfall. Investigation of genetic response and genes underlying acclimation into diverse climatic conditions is important for poultry industry since it can improve our understanding of mechanisms of environmental adaptation process in chickens and serve as a molecular basis for efficient breeding toward temperature stress tolerance [15, 22–24]. A useful high throughput approach to tackle a jigsaw of potential and relevant genes associated with adaptation, performance and phenotypic traits of interest is a genome-wide search for selection signatures. These can be discovered using different methodologies, e.g., by determining contiguous sequences of homozygous identical-by-descent haplotypes, known as runs of homozygosity (ROHs), or inferring fixation index (*F*_ST_) of genomic windows as a measure of genetic differentiation (e.g., [8–10, 14, 15, 24]).

Given that USH, OMF, and RUW are cold tolerant, the purpose of this study was to identify and compare the respective footprints of selection in the two old breeds, USH and OMF, with the previous [10] and new, expanded, data collected for defining the genomic features of RUW. With this in mind, we evaluated the genomic architecture and traces of selection in USH, previously understudied in terms of its genetic and genomic features, in comparison with three other breeds including OMF, RUW and WCR. Accordingly, we searched for loci under selection pressure that can be associated with phenotypic traits of interest in USH and other breeds.

## Methods

### Experimental animals

Chickens of the USH and OMF breeds (Fig. 1C, D) developed ~ 150–200 years ago in the conditions of Russian local farms were used in the present study. In addition, the analysed dataset included the RUW and WCR breeds (Fig. 1A, B) divergently selected for contrasting traits of egg and meat performance, respectively. Birds of the USH, OMF and WCR breeds were purchased from the Federal Research Centre “All-Russian Poultry Research and Technological Institute” (FRCARPRTI) and placed in the bioresource Gene Pool Collection of Farm and Wild Animals and Birds at the L.K. Ernst Federal Research Centre for Animal Husbandry (LKEFRCAH). Samples of the RUW breed were provided by the Russian Research Institute of Farm Animal Genetics and Breeding (RRIFAGB).

### Sample collection and DNA extraction

The total sampling size was 156 animals including 40 USH, 30 OMF, 64 RUW and 22 WCR individuals. DNA extraction from chicken feather samples was performed using a commercial nexttec™ 1-Step DNA Isolation kit equipped with nexttec™ cleanColumns (Nexttec Biotechnologie GmbH, Leverkusen, Germany) and following the manufacturer’s protocols. Solutions of the isolated DNA were quality controlled by measuring the double-stranded DNA concentration using a Qubit 3.0 fluorometer (Thermo Fisher Scientific, Wilmington, DE, USA) and determining the ratio of the DNA light absorbance values at 260/280 nm using a NanoDrop-2000 spectrophotometer (Thermo Fisher Scientific, Wilmington, DE, USA).

### SNP genotyping and quality control

Sample genotyping was performed using the Chicken 50K_CobbCons SNP chip (Illumina, San Diego, CA, USA), and there were 53,872 SNPs before all filters. Input files for analysis and subsequent visualization of the results were prepared using the R software environment [25]. Genotyping quality was controlled using PLINK 1.9 software [26] and applying the following filters: at least 90% of loci (--geno 0.1) were successfully genotyped in at least 90% of samples (--mind 0.1), and the frequency of minor alleles was at least 5% (--maf 0.05). Only SNPs located on 28 autosomes (GGA1 to GGA28) were considered for the further analysis. After filtering, the genotype dataset to search for and analyse signatures of selection included 44,339 autosomal SNPs. For certain types of analyses, i.e., genetic diversity assessment, principal component analysis (PCA), Neighbor-Net plotting, and admixture estimation, an additional filter was applied by introducing > 50% linkage disequilibrium threshold (using the PLINK --indep-pairwise 50 5 0.5 command), after which 28,993 SNPs were obtained. Coordinates of SNP positions in the GGA reference genome assembly GRCg6a [27] were accepted in this study.

### Genetic diversity and population structure

Using the R package diveRsity [28], the following indicators were calculated to assess within-breed genetic diversity: the observed heterozygosity (*H*_*O*_), unbiased expected heterozygosity (_*U*_*H*_*E*_) [29], rarefied allelic richness (*A*_*R*_) [30], and _*U*_*H*_*E*_-based inbreeding coefficient (_*U*_*F*_IS_).

Genetic differences between the studied breeds were ascertained in PLINK 1.9. PCA visualization was performed in the R package ggplot2 [31]. Pairwise distances for identical-by-state regions were applied to construct a Neighbor-Net dendrogram using the SplitsTree 4.14.5 program [32].

Model-based clustering was fulfilled for refining population structure using the ADMIXTURE v1.3 software [33]. The optimal number of clusters (ancestral populations) K was determined using the lowest error in the cross-validation procedure as calculated for K values from 1 to 8. Visualization of the admixture analysis results was performed using the R package pophelper [34].

### Selective sweeps

To search for signals of selective sweeps in the studied breeds, the following three methods were used: (1) assessment of ROH islands overlapping in different breeds, (2) calculation of *F*_ST_ values in pairwise comparison of breeds, and (3) haplotype differentiation analysis.

#### ROH mining

To compute ROHs, a window-free method for consecutive SNP-based detection [35] was employed as implemented in an R package detectRUNS [36]. During this analysis, one SNP with a missing genotype and one heterozygous SNP were admitted avoiding an underestimation of ROHs with a length of 8 megabases (Mb) or more [37]. The minimum allowable ROH length was 500 kilobases (kb) to exclude too short and widespread regions from the analysis. The minimum number of SNPs (*l*) was calculated according to the method proposed by Lencz et al. [38] and modified by Purfield et al. [39]:

$$l=\frac{{log}_e\frac\alpha{n_s\times n_i}}{{log}_e(1-\overline{het})},$$

where *n*_*s*_ is number of SNPs genotyped in an individual sample, *n*_*i*_ is number of genotyped individuals, *𝛼* is proportion of false-positive ROHs (set to 0.05 in this study), and $\overline{het}$ is mean heterozygosity for all SNPs. Using this formula, we found *l* = 24 in our case.

Based on information about the number and length of homozygous regions in the analysed breed genomes, the genomic inbreeding coefficient (*F*_ROH_) was calculated, which was the ratio of the sum of lengths of all ROH per individual to the total length of the chicken reference genome covered with autosomal SNPs (~ 940 Mb). Homozygosity segments were distributed according to the length of the detected regions between the following length classes: 0.5–1, 1–2, 2–4, 4–8, 8–16 and > 16 Mb. To determine the proportion of the genome covered by ROH segments of various lengths, we figured out the sum of ROH in the following length classes: > 0.5, > 1, > 2, > 4, > 8, and > 16 Mb. Suggestive ROH islands were defined as homozygous regions overlapping by 0.3 Mb that were shared by more than 50% of the analysed individuals in each breed.

#### *F*_ST_ estimation

Pairwise values of genetic distances between all SNPs based on *F*_ST_ were calculated in PLINK 1.9. The top 0.1% *F*_ST_ values served to detect a selection signature as was propounded elsewhere [40].

#### HapFLK procedure

The hapFLK 1.4 program [41] was used to analyse the haplotype differentiation in the studied breeds to define footprints of selection. The number of haplotype clusters was revealed during the cross-validation procedure in fastPHASE program [42] and was 35. The results of the hapFLK test were visualized using the qqman package [43]. The hapFLK regions with at least one SNP at the *P*-value threshold of 0.01 (–log_10_(*P*) > 2) were chosen for detailed analyses.

### Search for candidate genes and QTLs within selective sweep regions

In the genomic regions under putative selection as recognized by the three different statistics, i.e., *F*_ST_, ROH, and hapFLK methods, candidate genes were mined. For this, lists of potential regions and genes under selection pressure were established if they were identified by two and more methods or in two and more breeds. For the ROH and hapFLK statistics, we searched for genes that fall entirely or partially within the given boundaries of the found intervals. For the *F*_ST_ statistic per single SNP, we looked at genes falling within the window of ± 200 kb from the target SNP. In the case when more than one SNP was revealed, the boundaries of the interval for gene search were set as follows: − 200 kb from the position of first SNP and + 200 kb to the position of last SNP.

For structural annotations of the above selective sweep areas, chicken genes inside the chosen regions and their human orthologs were retrieved from the Ensembl Genes release 106 database using BioMart data mining tool [44] as described elsewhere [10]. The list of selected genes was manually curated and supplemented with other focused genes if those were reported previously (e.g., [10, 15, 22, 24]) for the selective sweep regions found in the present study.

Furthermore, to broaden the candidate list with more previously discovered and significant genes, a more extensive gene excavating was completed that encompassed the regions identified by one technique. Afterwards, National Center for Biotechnology Information PubMed-available information from other published studies was analysed for functional annotation of all candidates and selection of prioritized candidate genes (PCGs) that were the most relevant for characterizing phenotypic and genomic features of the chicken breeds investigated. Distribution of PCGs among the four breeds studied was visualized by plotting Venn diagrams [45]. We also looked at a publicly available database, Chicken QTLdb [46], to see if there were any quantitative trait loci (QTLs) and associated genes that corresponded with the detected genomic regions.

## Results

### Between- and within-breed genetic diversity

**Fig. 2:**
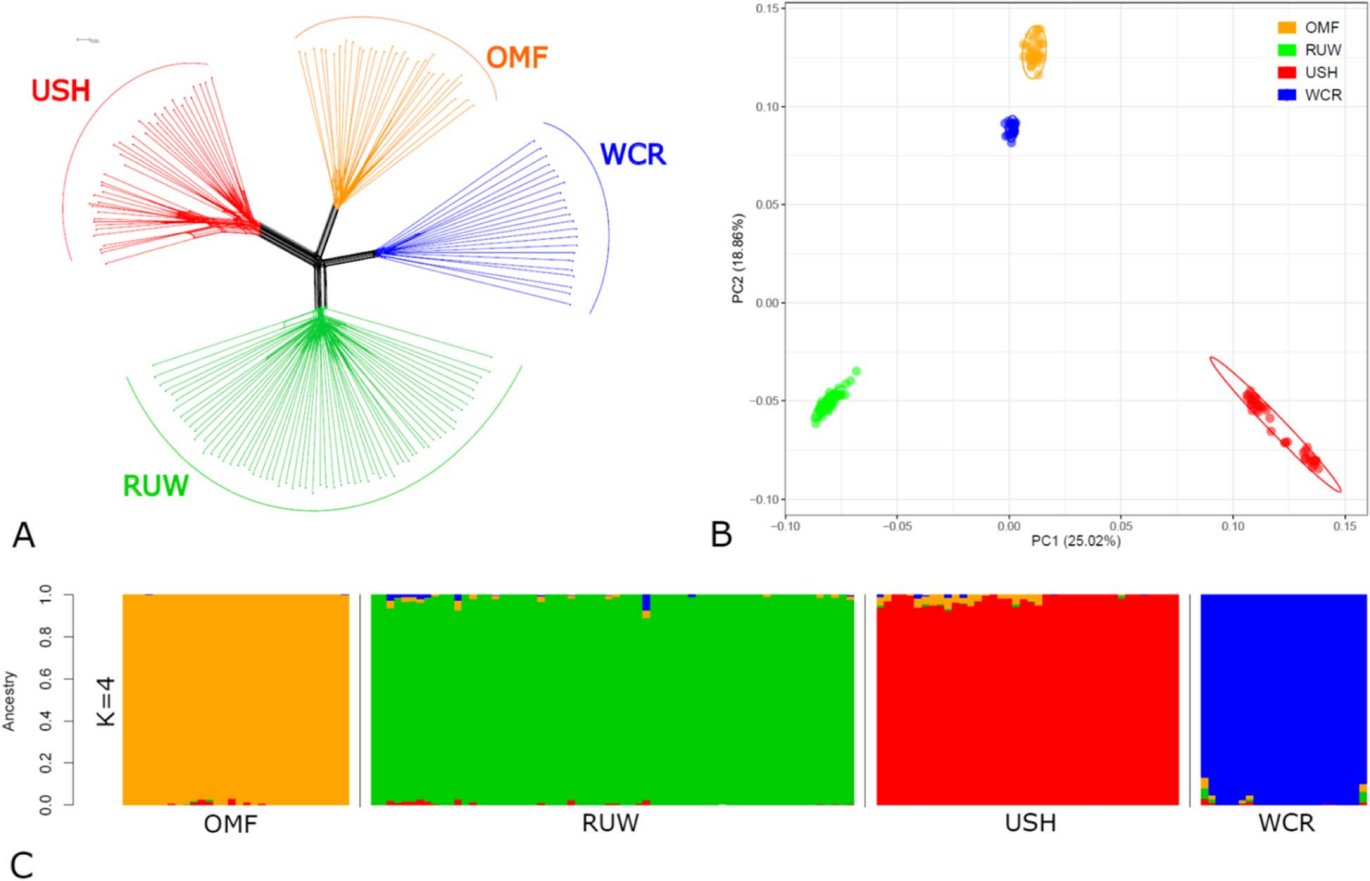
Chicken breed relationships based on genome-wide SNP genotypes. **A** Neighbor-Net tree constructed using the *F*_ST_ genetic distances within and between the studied populations; **B** PCA plot showing the distribution of breeds and individuals in the dimensions of two coordinates, i.e., the first (PC1; *X*-axis) and second (PC2; *Y*-axis) principal components, with respective percentage of the total variance, which can be explained by each of the two PCs; and **C** ADMIXTURE bar plot representing individual ancestry proportions in the studied populations at K = 4. Breeds: OMF, Orloff Mille Fleur; RUW, Russian White; USH, Ushanka; WCR, White Cornish

Using genotypes for a total of 28,993 validated genome-wide SNPs, the four breeds demonstrated a distinct genetic differentiation as visualized with the respective Neighbor-Net tree (Fig. 2A), PCA plot (Fig. 2B), and ADMIXTURE bar plot (Fig. 2C). As resulted from the clearcut PCA-inferred breed distribution, there were compact localization and appropriate breed assignment of all the genotyped individuals (Fig. 2B). The four breeds occupied distinctive positions on the PCA plot, with USH and RUW being the most distant and OMF and WCR being closer to each other.

Although K = 8 represented the most optimal and probable number of clusters (ancestral populations) (Fig. S1), each breed already revealed its own genetic structure at K = 4, with a very few instances of admixture and introgression from other breeds or use of few archived USH samples from 2011 to 2012 (Fig. 2C), which was important for subsequent search for loci under selection pressure. The within-breed population structure and admixture patterns for K = 2, 3 and 8 are given in Fig. S2. Specifically, USH and RUW were characterized with a single ancestry at K = 2, while OMF and WCR had two ancestries. When K = 3, there were single ancestries in USH, OMF and RUW, with WCR showing three ancestries. At K = 8, we observed three ancestries in USH, two in OMF and RUW, and one in WCR.

When within-breed diversity parameters were assessed (Table 1), the highest genetic variability was observed in WCR and the lowest one in USH, with OMF and RUW having intermediate values of *H*_*O*_, _*U*_*H*_*E*_ and *A*_*R*_ (*P* < 0.001). Judging from _*U*_*F*_IS_ values, USH (0.055) was characterized by an increased excess of homozygotes under Hardy–Weinberg equilibrium, with a deficiency of heterozygotes being also found in WCR (0.015) and their slight excess in RUW (–0.004), while OMF was almost close to the Hardy–Weinberg equilibrium state (0.001; Table 1).

**Table 1:** Descriptive statistics for genetic diversity indices in the four breeds studied^1^

Breed	*n*	*H*_*O*_ (M ± SE)	_*U*_*H*_*E*_ (M ± SE)	*A*_*R*_ (M ± SE)	_*U*_*F*_IS_ [CI 95%]
OMF	30	0.305 ± 0.001	0.305 ± 0.001	1.847 ± 0.002	0.001 [–0.001; 0.003]
RUW	64	0.332 ± 0.001	0.330 ± 0.001	1.927 ± 0.001	–0.004 [–0.006; − 0.002]
USH	40	0.246 ± 0.001	0.263 ± 0.001	1.787 ± 0.002	0.055 [0.053; 0.057]
WCR	22	0.373 ± 0.001	0.379 ± 0.001	1.969 ± 0.001	0.015 [0.012; 0.018]

^1^ Breeds: *OMF* Orloff Mille Fleur, *RUW* Russian White, *USH* Ushanka, *WCR* White Cornish. *n* number of individuals, *H*_*O*_ observed heterozygosity, *M* Mean value, *SE* Standard error, _*U*_*H*_*E*_ unbiased expected heterozygosity, *A*_*R*_ rarefied allelic richness, _*U*_*F*_IS_ unbiased inbreeding coefficient [CI 95%, range variation of _*U*_*F*_IS_ coefficient at a confidence interval of 95%]. All pairwise breed differences were significant at *P* < 0.001

Characterization of within-breed genome-wide homozygosity degree in terms of ROH metrics (Table 2) revealed that USH had the highest mean overall ROH length and respective inbreeding coefficient (*P* < 0.001). In WCR, these parameters had the lowest values, while in OMF and RUW they were intermediate (*P* < 0.001). Mean ROH number values were the highest in USH and OMF as compared to the lowest one in RUW (*P* < 0.05).

**Table 2:** Descriptive statistics for runs of homozygosity (ROHs) in the four breeds studied^1^

Breed	*n*	Mean length of ROHs, Mb (M ± SE)	Mean number of ROHs (M ± SE)	*F*_ROH_ (M ± SE)
OMF	30	265.30 ± 12.21	125.20 ± 4.49^ab^	0.283 ± 0.013
RUW	64	206.71 ± 4.41	112.80 ± 1.57^c^	0.220 ± 0.005
USH	40	411.31 ± 13.46	126.50 ± 2.50^a^	0.438 ± 0.014
WCR	22	160.46 ± 4.43	117.82 ± 2.75^bc^	0.171 ± 0.005

^1^ Breeds: *OMF* Orloff Mille Fleur, *RUW* Russian White, *USH* Ushanka, *WCR* White Cornish. *n* number of individuals, *M* Mean value, *SE* Standard error, *F*_ROH_ inbreeding coefficient inferred from mean ROH lengths. Values with the same superscript have no significant difference

**Fig. 3:**
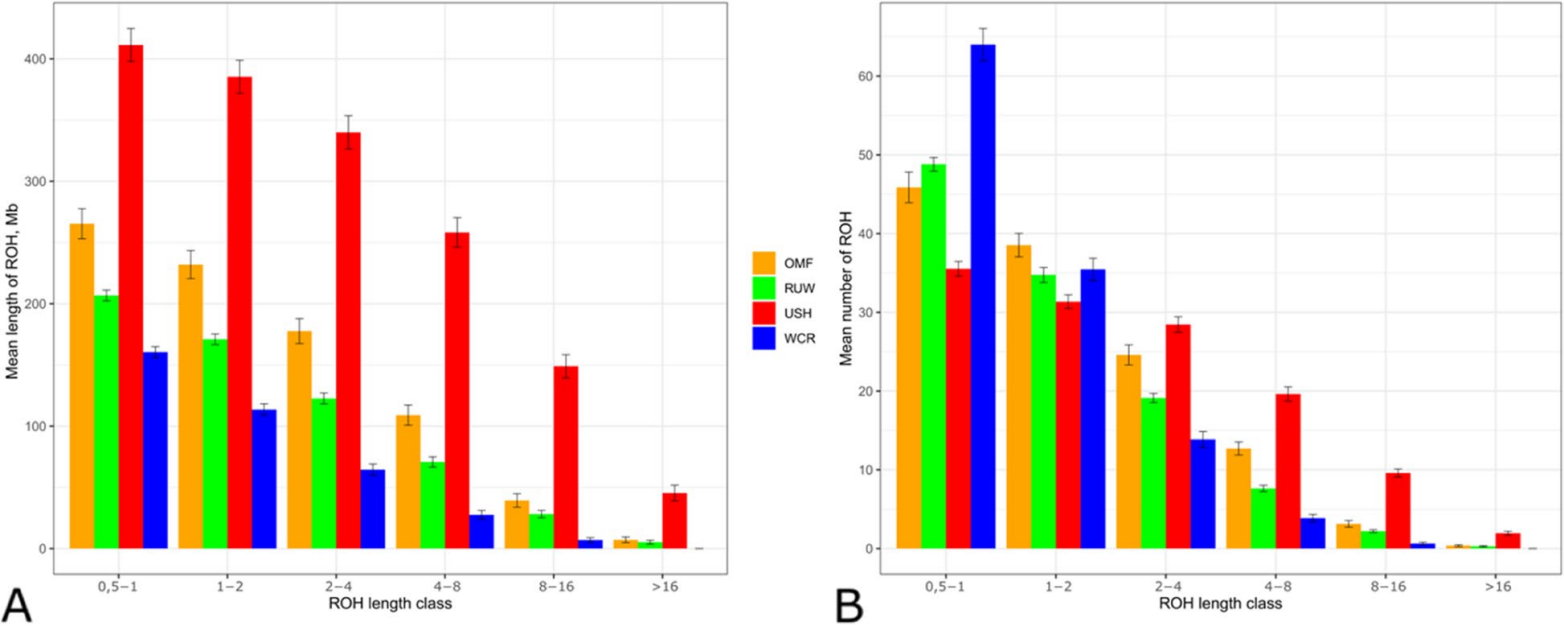
Runs of homozygosity (ROH) patterns in the four breeds studied. Distribution of ROHs is shown by ROH length class (*X*-axis; 0.5–1, 1–2, 2–4, 4–8, 8–16, and > 16 Mb) by their mean length (**A**) and number (**B**) (*Y*-axis). Breeds: OMF, Orloff Mille Fleur; RUW, Russian White; USH, Ushanka; WCR, White Cornish

ROH distribution analysis (Fig. 3, Table S2) showed that USH had the greatest overall ROH lengths across various ROH length classes, with OMF, RUW and WCR having the second, third and fourth much shorter overall ROHs, respectively (Fig. 3A). Concerning ROH numbers among length classes (Fig. 3B), there was the greatest number of shortest ROHs in WCR that was essentially reduced in longer ROH classes. A somewhat opposite distribution pattern was inherent in USH that had fewer shorter ROHs (up to 2 Mb) relative to other breeds, however this breed was superior to others for the longer ROH classes.

### Signatures of selection

#### ROH islands

On 22 out 28 GGA autosomes, there were a total of 256 ROH islands (Table 3 and S3), with their greatest number being identified in USH (165), an intermediate number in OMF (55), and the lowest ones in WCR (19) and RUW (17). The appropriate distribution of ROH islands by chromosomes (Table 3) showed the greatest genome coverage per chromosome in USH (*P* < 0.05) and the lowest one in OMF and RUW (at *P* < 0.001 relative to USH).

**Table 3:** Runs of homozygosity (ROH) islands identified in the genomes of the studied chicken breeds^1^

GGA	No. of ROH islands				Coverage by ROH islands, Mb			
	**OMF**	**RUW**	**USH**	**WCR**	**OMF**	**RUW**	**USH**	**WCR**
1	12	5	33	4	11.927	5.594	67.422	6.584
2	9	2	22	7	9.986	2.415	50.988	8.074
3	8	4	16	1	6.731	3.222	17.116	0.463
4	4	3	20	2	2.491	3.739	34.117	1.513
5	3	1	12	–	2.334	0.903	17.723	3.240
6	–	–	2	–	–	–	3.931	–
7	6	–	11	1	5.201	–	19.405	0.571
8	1	–	6	1	0.883	–	7.851	0.586
9	1	1	3	–	0.530	0.525	4.776	–
10	2	–	3	–	0.952	–	7.785	–
11	2	–	7	–	1.242	–	9.060	–
12	5	1	5	–	3.192	0.640	8.495	–
13	–	–	3	–	–	–	2.618	–
14	1	–	3	–	0.700	–	4.070	–
15	–	–	2	–	–	–	2.406	–
18	–	–	1	1	–	–	1.506	0.789
19	–	–	3	–	–	–	2.623	–
20	–	–	3	–	–	–	2.223	–
21	–	–	1	–	–	–	0.769	–
26	1	–	2	–	0.828	–	0.919	–
27	–	–	1	–	–	–	1.978	–
28	–	–	4	1	–	–	3.338	0.679
Total	55	17	165	19	46.998	17.038	272.987	22.500
Mean length (M ± SE)					1.821 ± 0.064^ab^	1.891 ± 0.143^ac^	2.638 ± 0.132	2.075 ± 0.183^bc^

^1^ Breeds: *OMF* Orloff Mille Fleur, *RUW* Russian White, *USH* Ushanka, *WCR* White Cornish. *M* Mean value, *SE* Standard error. Mean values with the same superscript have no significant difference

On 12 autosomes, there were 32 ROH regions overlapped in two or three breeds with the respective distribution of ROH islands among chromosomes (Table 4, Fig. S3), including 28 such regions in USH, 22 in OMF, 8 in WCR, and 7 in WUR.

**Table 4:**
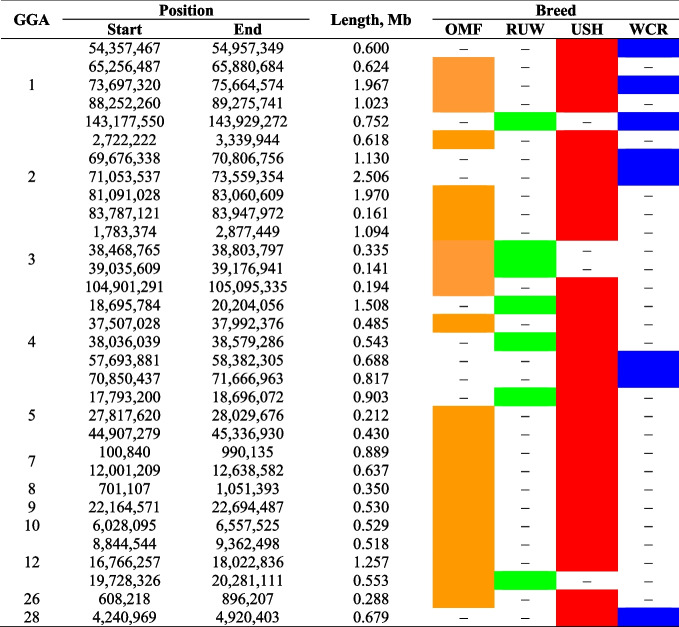
Overlapped ROH islands in the genomes of the studied chicken breeds^1^

^1^ Breeds: *OMF* Orloff Mille Fleur, *RUW* Russian White, *USH* Ushanka, *WCR* White Cornish

#### *F*_ST_ statistic at pairwise comparison of breeds

Based on the top 0.1% SNPs and distribution of SNPs by *F*_ST_ values at pairwise comparison of the studied breeds (Table S4, Fig. S4), we identified 15 blocks on ten autosomes with *F*_ST_ value ranging between 0.967 and 1.000 (Table 5). All these blocks were shared by USH, and five blocks by each of OMF, WCR and RUW.

**Table 5:** *F*_ST_ values and blocks of SNPs joined by two or more top 0.1% neighboured SNPs at pairwise comparison of the four breeds studied^1^

GGA	Breed	No. of SNPs	Position		*F*_ST_ value
			**Start**	**End**	
2	USH/WCR	2	39,154,873	39,623,877	0.967
	USH/RUW	3	107,995,442	108,912,691	0.981–1.000
	USH/OMF	2	133,841,003	133,862,313	1.000
3	USH/RUW	2	10,305,039	10,908,971	0.981–0.990
	USH/RUW	4	23,769,071	24,130,581	0.980
5	USH/WCR	16	31,277,206	32,352,944	0.967–1.000
	USH/OMF	2	41,458,372	41,510,780	1.000
7	USH/WCR	2	20,333,216	20,504,792	0.967–0.983
8	USH/WCR	5	7,210,566	7,320,642	0.967–1.000
	USH/RUW	2	15,567,946	15,611,024	0.990
9	USH/RUW	2	6,080,490	6,252,976	0.980–0.981
12	USH/OMF	2	17,199,402	17,729,551	1.000
26	USH/OMF	2	390,677	503,333	1.000
27	USH/OMF	2	3,802,896	3,859,021	0.986–1.000
28	USH/WCR	4	4,330,208	4,522,560	0.967–1.000

^1^ Breeds: *OMF* Orloff Mille Fleur, *RUW* Russian White, *USH* Ushanka, *WCR* White Cornish

#### HapFLK statistic

**Fig. 4:**
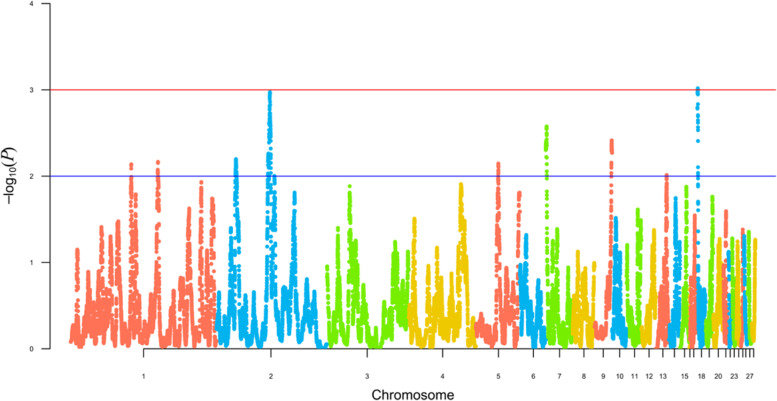
Search for signatures of selection in the four breed genomes as revealed by the hapFLK analysis. Values for the *X*-axis are chicken autosomes, and those for the *Y*-axis are values of statistical significance (–log_10_*P*-values). The blue line indicates threshold of significance at *P* < 0.01 (i.e., –log_10_(*P*) > 2), with the red line conforming to *P* < 0.001 (i.e., –log_10_(*P*) > 3)

Using HapFLK analysis, eight regions of selection signatures were discovered on six autosomes (Fig. 4; Table 6). Six of these HapFLK blocks were found in USH, five in OMF, three in WCR, and one in RUW, with six blocks being shared between two or three breeds.

**Table 6:** HapFLK blocks revealed in the genomes of the studied chicken breeds^1^

GGA	Breed	Position		Length, Mb	No. of SNPs	Most significant SNP	*P*-value
		**Start**	**End**				
1	USH, OMF	82,083,018	82,184,177	0.101	6	82,184,177	7.29E-03
	USH	117,913,388	118,392,895	0.480	14	118,357,776	6.83E-03
2	OMF, WCR	25,717,703	27,076,503	1.359	46	26,292,612	6.35E-03
	USH, WCR	69,676,338	73,482,942	3.807	117	72,332,610	1.06E-03
5	OMF	30,484,744	30,832,693	0.348	13	30,660,125	7.14E-03
7	USH, OMF	100,840	1,540,849	1.440	64	1,327,148	2.66E-03
9	USH, RUW	23,051,176	24,121,334	1.070	56	23,581,143	3.85E-03
18	USH, OMF, WCR	36,059	824,291	0.788	42	493,446	9.58E-04

^1^ Breeds: *OMF* Orloff Mille Fleur, *RUW* Russian White, *USH* Ushanka, *WCR* White Cornish

Finally, we compiled genomic regions with the signals of selection sweeps identified in the genomes of the four studied chicken breeds by three different statistics (Table S5) and used this list for candidate gene/QTL search as outlined in the two subsections below.

### Candidate genes affected by selection

Across 23 autosomes (GGA1–GGA15, GGA18–GGA21, GGA23, and GGA26–28), we revealed 77 genomic regions that demonstrated footprints of selection in two and more breeds or supported by two and more methods. We also included 38 regions found in one breed and identified by one method when these encompassed genes of interest known from our previous investigation [10] and other relevant studies (Table S6). Structural annotation of these 115 regions resulted in a total of 3925 chicken genes. Of those, 2373 genes were candidates annotated in chicken, with the rest being 1349 Ensembl novel genes (including 96 genes orthologous to known human genes), 87 uncharacterized loci (including 21 genes homologous to annotated human genes), and 115 microRNAs (with 16 homologous to human microRNAs). In all, there were 2220 orthologous human genes, with 2177 being annotated, 23 uncharacterized and 20 Ensembl novel genes. Descriptive characteristics of all candidate genes under putative selection pressure were provided in Table S6.

Functional annotation of candidate genes led to a suite of 540 PCGs previously reported elsewhere (e.g., [10, 15, 22, 24]; as summarized in Table S6) that could be under selection and responsible for specific phenotypic traits in the four breeds investigated. These genes were distributed among 22 autosomes (as shown in Table S7) and were also divided into 12 functional categories based on phenotypic features they are related to as follows (with respective gene numbers given in parentheses): cold tolerance (84); domestication (6); egg traits (68); energy and feed intake (27); fat metabolism (83); growth, meat, carcass (131); immunity (140); reproduction (55); response to heat (35); skin, feather, other skin appendages (76); stress and adaptation (47); and thermosensation (5) (Table S8). Several genes previously suggested to influence more than one phenotypic trait were attributed to more than one functional category.

**Fig. 5:**
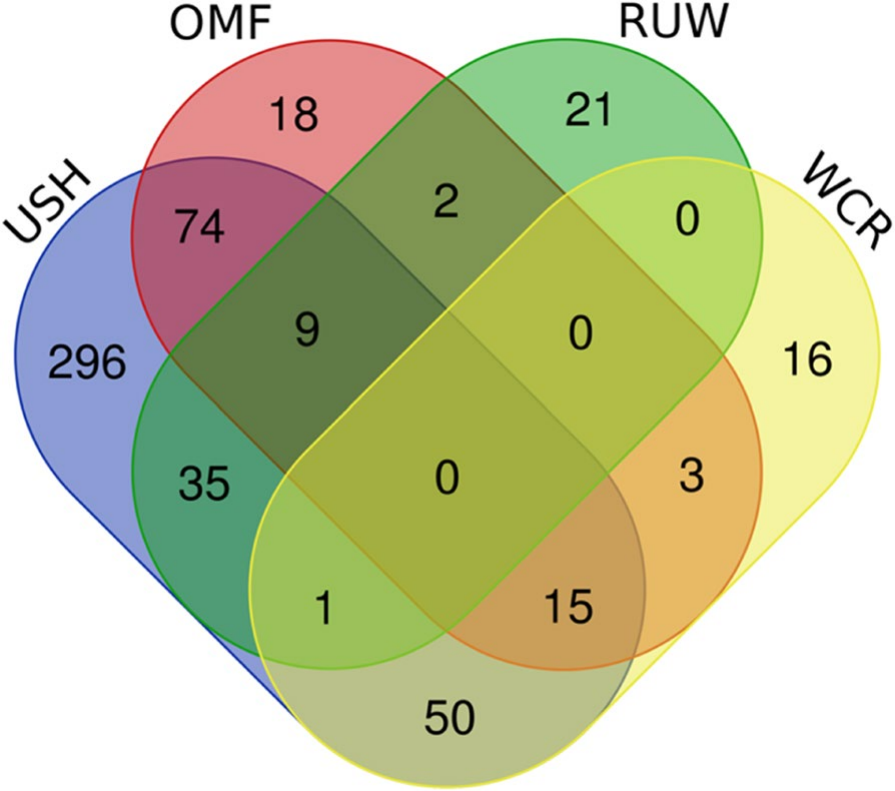
Venn diagram representing distribution of 540 prioritized candidate genes between the four breeds studied. OMF, Orloff Mille Fleur; RUW, Russian White; USH, Ushanka; WCR, White Cornish

We also analysed distribution of the 540 PCGs among the four breeds and found out that USH had their greatest number (480, or 89%), while OMF, WCR and RUW had lower numbers: 121 (22%), 85 (16%) and 68 (13%), respectively (Table S9). The sharing pattern of the PCGs is given in Fig. 5. According to it, USH had 296 unique genes (62% relative to the breed’s gene number), whereas we observed just 21 (17%), 18 (21%) and 16 (24%) unique genes in RUW, OMF and WCR, respectively. Pairwise comparison of shared PCGs between two breeds showed that their greatest number was between USH and OMF (98, or 20% as compared to the USH gene number), and USH also shared 66 genes (14%) with WCR and 45 genes (9%) with RUW. There were 18 genes shared between OMF and WCR (15% relative to the OMF gene number) and 11 genes between OMF and RUW (9%), with just one gene shared between WCR and RUW (1% as compared to the WCR gene number). No gene was shared by all the four breeds.

Venn diagrams for the 540 PCGs distributed by breed by phenotypic category are demonstrated in Fig. S5, and their summary is provided in Table 7. In all instances (except domestication), USH was represented with the greatest numbers of unique and shared genes in each category.

**Table 7:** Numbers of unique and shared prioritized candidate genes by phenotypic category across the studied chicken breeds^1^

Phenotypic category^2^	*n*^3^	Unique genes				Shared genes			
		**USH**	**OMF**	**RUW**	**WCR**	**USH**	**OMF**	**RUW**	**WCR**
Cold tolerance	84	60	0	5	0	13	9	3	1
Domestication	6	0	1	2	0	3	2	1	2
Egg traits	68	44	1	2	1	21	8	3	12
Energy and feed intake	27	22	1	0	1	8	5	3	2
Fat metabolism	83	38	5	2	2	21	19	4	2
Growth, meat, carcass	131	74	3	6	5	41	24	10	17
Immunity	140	69	3	5	5	57	34	18	14
Reproduction	55	28	0	2	2	23	14	6	7
Response to heat	35	19	1	0	1	13	10	3	5
Skin, feather, skin other appendages	76	37	2	5	5	26	15	8	6
Stress and adaptation	47	27	4	1	1	17	7	8	2
Thermosensation	5	3	0	0	0	2	2	0	1

^1^ Breeds: *OMF* Orloff Mille Fleur, *RUW* Russian White, *USH* Ushanka, *WCR* White Cornish. ^2^ Phenotypic categories were attributed to PCGs based on relevant PubMed publications (see Materials and Methods). ^3^ Total number of genes per category

### Overlapping with Chicken QTLdb loci

Using Chicken QTLdb [46], search for QTLs overlapping with selection footprints was performed to validate the identified genomic regions harbouring these signals of selective sweeps and, accordingly, potential genes underlying phenotypic traits in the four chicken breeds. As a result, 2460 known QTLs were revealed that also functionally overlapped with the candidate genes we detected in the present study. Table S10 contains a list of 302 Chicken QTLdb loci assigned to traits potentially linked to adaptability and acclimation as well as 81 associated genes (including 42 PCGs) also found within the selective sweeps in our investigation. For those, we counted 97 combinations of phenotypic traits, regions under suggestive selection pressure and 256 QTLs observed in USH, 31 in OMF, 19 in RUW, and 13 in WCR. Eight QTLs were potentially related to thermoregulation; all of them were found in USH, with one shared with WCR and OMF, too (Table S10).

## Discussion

### Between- and within-breed genetic diversity

It is well known that the genomes of chicken breeds spread throughout the world have been shaped by past and recent admixture events [4]. From analysing the breed structure and admixture patterns (Fig. 2C and S2), we can see that the two original ancestral populations are likely to correspond to the two main roots of domesticated chickens [47] (as shown in Fig. S2 with green and red colours, respectively): (1) Mediterranean, or egg-type (with RUW as a prime example), and (2) Asiatic, or meat-type. At K = 3, we observe the addition of another evolutionary branch of chicken breed formation, the game one (orange colour in Fig. S2). This origin branch is solely presented in OMF, while WCR is clearly subdivided into all the three branches: red Asiatic (due to the meat-type Cochin breed among WCR ancestors), green European (ancestral local chickens of England), and orange game (original game breeds used to create WCR).

At K = 4, all the breeds are well separated, with minor inclusions of other genotypes. Eventually, at optimal K = 8 (Fig. S2) we can see a very minor admixture between breeds and a clear division of the three domestic breeds into ancestral subpopulations. On the RUW chart, noteworthy is the contribution of two parent breeds, White Leghorn and local Russian laying hens, and two subpopulations, FRCARPRTI and RRIFAGB, genotyped in this study and diverged into two independent branches. In OMF forming its own cluster at K = 3 and 4 (Fig. S2 and 2C), there are also two initial components, Malay (game) type chickens and some local chickens, which is reflected in the OMF breed pattern at K = 8 (Fig. S2).

Of even greater interest is the oldest USH breed divided into two clusters (at K = 6; data not shown) and even into three clusters (at K = 8; Fig. S2). As we understand it, this intra-breed pattern, on the one hand, is due to the use of samples collected 10 years ago and use of the current ones. On the other hand, this may also reflect the contribution of unknown ancestral populations to the formation of USH. This aspect of the creation and maintenance of the USH gene pool with a small population size requires further detailed study.

The resulting intra-breed structure patterns are also confirmed by the PCA-based topology of the four breeds, with a very compact arrangement of WCR individuals and a sparser arrangement in three other breeds, especially USH (Fig. 2B). The close position of OMF and WCR on the PCA plot, in principle, is well explained, since game chickens participated in the development of these breeds. A separate position was observed in the egg-type RUW breed (two subpopulations). As for USH, it also shows very unique genotypes and is located almost equidistant from all other breeds, confirming that it is a very distinctive breed. Overall, the obtained patterns of intra- and inter-breed genetic differences (Fig. 2A–C) are in very good agreement with breed history and demographics.

### Signatures of selection

In terms of genomic regions under selection pressure, the largest number of ROH islands (165 out of 256; Table S5) was found in USH, which indicates a very pronounced originality of this breed.

Considering RUW recently examined by us [10], we extended its sample size to 71 individuals and included this breed in the present analysis in order to confirm previously identified pressure loci in a larger sampling and directly compare these data with USH and two other breeds. Since the WCR sampling did not change, we did not expect changes in loci under selective pressure in this breed.

Using a larger sample size in RUW, we confirmed ROH in the region of 123.0.124.3 Mb on GGA2, which was previously identified by us [10] and seems to be associated with body temperature regulation [10, 12]. The same region somewhat overlapped in the region of three genes (*CALB1*, *DECR1* and *NBN*) with the neighbouring ROH (124.3.125.2 Mb; Table S6) that is under selection pressure in USH, which can be considered as evidence of a shared, to some extent, nature of cold tolerance in these breeds. This finding seems to us very important and is also supported by the study of Fedorova et al. [15], where six candidate genes for cold tolerance in RUW are proposed in the 123.0.125.1 Mb region. In a genome-wide association study, Kudinov et al. [12] defined in the same region one more gene, *MMP16*, as a candidate for chick down colour in the RUW subpopulation at RRIFAGB, specially selected for cold tolerance. In OMF, this region of potential selection for cold tolerance was not identified by us.

Thus, a common region has been outlined on GGA2 for association with thermoregulation in RUW and USH. Note that OMF, in principle, should also demonstrate cold tolerance, possibly related to other genomic region(s) and candidate genes, which requires further research.

### Candidate genes affected by selection

We performed a detailed structural and functional annotation of regions and genes under selection pressure by developing an effective strategy. This strategy included establishing lists of genes in regions identified in each breed by at least two methods (true selective sweep regions), and genes localized in areas of overlapping regions identified by one method (e.g., ROH) in two or more breeds. In addition, we also added lists of genes localized by one method separately for each breed if these genes were identified as important candidates in other investigations.

#### PCGs

Many regions and PCGs (Table S6) were not identified in our previous study using SNP genotypes of only two breeds, RUW (with a smaller sample size) and WCR [10]. For example, USH and OMF had four genes (*TMEM168*, *IFRD1*, *DOCK4* and *IMMP2L*) previously not found [10], involved in immune response and also playing a role in response to heat stress and in muscle growth and differentiation. On GGA1 there was a peculiar cluster of genes associated with reproductive traits and eggshell quality (including shell colour): *PIK3C2G*, *PLCZ1*, *CAPZA3*, *SLCO1C1*, *SLCO1B3*, *SLCO1A2*, *IAPP*, and *TSPO*. Below we will highlight main genes, first of all, those that can directly affect thermoregulation, cold tolerance and general adaptability in chickens.

#### Candidates for thermoregulation and cold tolerance: breed comparison

A number of PCGs we found (Table S6) can be linked to the adaptive abilities of chickens in terms of thermoregulation and cold tolerance. One of them is the *SOX5* gene, which affects the comb shape. Being a skin appendage, comb is a character of sexual dimorphism in chickens (i.e., associated with reproduction [48]) and, along with other integumentary tissues of wattles, can simultaneously perform an important thermoregulatory function, redirecting blood flow to the skin and allowing for heat exchange during high temperatures [49]. In particular, *SOX5* is responsible for the formation of a reduced pea comb [50]. In classical chicken genetics, this character is controlled by the dominant allele at the respective locus denoted by the symbol *P* [5, 6]. When interacting with another dominant allele, *R*, at the rose comb locus [48], an even more reduced walnut comb is formed [5]. Pea comb is sometimes found in USH and WCR, and walnut comb is a breed character of the Orloff chickens [5, 20]. In 1985, USH was examined at the FRCARPRTI collection farm by the Moscow Institute of General Genetics researchers: there were birds with single (R*N gene), rose (R*R gene) and pea (P*P gene) combs ([51], p. 365). With regard to the walnut comb in the Orloff breed and its association with cold tolerance, it was reported that adult roosters and hens tolerate frost well, do not freeze combs, and can be kept in unheated poultry houses [20]. The previously undescribed region on GGA1 (64.0.68.5 Mb) corresponds to overlapping ROHs in USH and OMF, and their overlay occurs precisely in the *SOX5* gene (Table S6); this seems to be a fairly weighty argument in favour of suggesting a possible relationship between *SOX5* and cold tolerance.

Recently, Xu et al. [24] reported candidate positively selected genes (PSGs) for cold tolerance found in a local Canadian Chantecler breed by three methods in one GGA1 region (190.1.190.3 Mb), e.g., *PRSS23*, *ME3*, *FAM181B*, *PRCP* and *DDIAS*. In our case, this region was observed in USH as a ROH, and also by the *F*_ST_ method in USH and OMF (both cold tolerant). Furthermore, when seeking functional candidates for cold tolerance, additional genes were found in the respective ROH regions in USH, which were also potential PSGs for cold tolerance in Chantecler chickens [24] (Table S6). Three more relevant genes were found within the ROH regions in USH, including two potential genes of arctic adaptation in sled dogs [52], *APOO* and *TRPV2*, as well as *FGF5*, a putative gene of local adaptive evolution in goats (as reviewed in [24]). One of these genes, *APOO* (apolipoprotein O), is also remarkable for being involved in lipid metabolism [53], downregulated in pectoralis major, and associated with lower intramuscular fat deposition in fast-growing chickens at hatch [54]. Moreover, *APOO* was localized within a region with confirmed selection signal in Korean native chickens [9] and is a candidate gene associated with egg production performance from 37 to 50 weeks [55]. In another GGA1 region (188.7.195.7 Mb), one more gene, *WNT11* (Wnt family member 11), can also be noted because it regulates traits that could potentially contribute to the development of protective mechanisms of cold tolerance. For instance, *WNT11* is a promising candidate gene for feathered-leg trait in chickens [56], being also expressed according to the moulting cycle [57] and required for dense dermis and subsequent cutaneous appendage formation [58]. In a GGA4 region (37.3.46.0 Mb), there is the *ACSL1* (acyl-CoA synthetase long-chain family member 1) gene that contributes to the antiviral response against avian leukosis virus subgroup J [59] and may be associated with feed efficiency, fat metabolism and heat stress [60]. Among the above genes, Xu et al. [24] especially distinguished *ME3* and *ZNF536* in two vital candidate regions related to cold tolerance in Chantecler chickens, which we also identified in the respective ROH regions in USH.

Within the OMF-specific ROH regions, the genes *EVC2* and *UNC79* were found, which were also candidate PSGs for cold tolerance in Chantecler chickens [24]. In total, out of 36 PSGs found for Chantecler by three methods [24], we have 23 genes in our study including 21 in USH and 2 in OMF.

Additionally, among the cold tolerance-related PSGs detected for Chantecler by two methods [24], six genes were revealed in USH: *PLCZ1*, *TYR* (known as a key gene for skin lightening in humans associated with cold adaptation in humans as reviewed in [24]), *HTR5A*, *EML5*, *LRP2*, and *KIF1B*.

Therefore, we have observed a significant overlay in the lists of candidate genes under selection pressure in Chantecler, USH, and, to some extent, OMF. At the same time, when comparing datasets for Chantecler and two North Chinese breeds, Xu et al. [24] did not find any match for major candidate genes. These ambiguous comparative data leave room for discussion and further in-depth study of the genetic control of cold resistance trait in such native Russian breeds as USH and in other similar breeds of the world gene pool.

It is also interesting to collate the data for signals of selective sweep obtained in a recent study [15] for RUW and in ours in terms of overlapping sets of identified candidate genes for cold tolerance. In comparison with the data by Fedorova et al. [15], we discovered the following overlaps of PCGs in regions under selection pressure in RUW: *HNF4G* (GGA2, 118.7.119.8 Mb); *WWP1*, *RIPK2*, *OSGIN2* and *DECR1* (GGA2, 123.0.124.3 Mb), the latter region being also identified in RUW in our previous investigation [10]. At the border with this region, we also observed the *CALB1* (calbindin 1) gene, the respective protein participating in eggshell calcification process [61]. Its activity in intestinal segments and eggshell gland was shown as negatively affected by high ambient temperature causing deterioration of eggshell quality characteristics under heat stress conditions [62]. The *CALB1* gene expression had a negative correlation with activity of immunoglobulin IgG2 [63] and was downregulated in cecum of *Salmonella* challenged chicks that also correlated with lower calcium content in blood [64].

In addition, we observed the following candidate genes for cold tolerance in USH that coincided with those in the study by Fedorova et al. [15]: *NECAB1*, *RUNX1T1*, *PRMT3*, *NELL1*, *ANO5*, *SLC17A6*, *GAS2*, *SLC5A12*, *FIBIN*, *LGR4*, *BDNF*, *NBEAL1*, *IDH1*, *PIKFYVE*, *PPP1R1C*, *SOCS3*, *AFMID*, *TK1*, *TMC6*, *GCGR*, *NPB*, *SIRT7*, *PYCR1*, *SPAG9*, *WFIKKN2*, *CACNA1G*, *ACSF2*, *CD300LG*, *FADS6*, *KCTD2*, and *MIF4GD*. We also detected three such genes (*GGPS1*, *COA6* and *DISC1*) in *OMF*, and one (*NDUFA4*), in both OMF and WCR (Tables S5 and S6). An important role of some of these genes in chickens has also been identified in other studies. For example, the neuropeptide *BDNF* (brain derived neurotrophic factor) gene is critically involved in thermal-experience-dependent development and plasticity; it was expressed in chicks in response to heat and cold exposure [65]. Yossifoff et al. [66] discovered that *BDNF* expression is regulated during thermotolerance acquisition via DNA methylation of the gene promoter. Kisliouk and Meiri [67] further investigated the *BDNF* gene function in thermal-control establishment. They revealed that activation or silencing of gene transcription in chick hypothalamus was regulated by histone modifications suggesting specific epigenetic role of chromatin modifications in thermal-control establishment. Goel et al. [68] supported an idea that an early stress response-related gene expression in the hypothalamus help cells is important in adaptation to an adverse environment. A homeostatic mechanism that connects hypothalamic energy management and body composition may exist as a result of interactions between BDNF, triiodothyronine (T_3_), and/or corticosterone [69]. There is also a potential role of one *BDNF* splicing variant in Marek’s disease (MD) tumour resistance and susceptibility [70]. *NELL1* is a domestication-related gene within a positive selective signature region [71] that is associated with selection on skeletal integrity that was probably co-selected with growth rate and meat yield in chickens [7, 72].

Collectively, we determined nine overlapping regions in two studies, Fedorova et al. [15], and the present one, that included 40 genes. Herewith, Fedorova et al. [15], similar to our study, found a selective sweep signal in RUW in the GGA2 region, 123.0.124.3 Mb, which presumably contains the candidate gene(s) for cold tolerance. Overall, there is a good agreement between the results of the two studies.

#### Thermoregulation and cold tolerance candidates related to thyroid hormones

We have found other examples of genes that may be involved in the genetic mechanisms of adaptation to low temperatures in chickens. For instance, Xie et al. [22] analysed the thyroid transcriptome in chickens in relation to adaptive responses to cold environmental conditions and drew attention to the *TPO* (thyroid peroxidase) gene localized on GGA3 and having 15 exons. In that study, thyroid transcriptomes were studied in response to low and normal temperatures in the cold tolerant Northern Chinese Bashang Long-tail breed and in Rhode Island Reds. It is known that the synthesis of thyroid hormones elevates in a cold environment in birds and mammals. In particular, at low temperatures the level of T_3_ grows, and the size and activity of the thyroid gland increase, too (as reviewed in [22]). The TPO enzyme (as an important element in the synthesis of thyroid hormones) had upregulated expression in these two studied breeds kept at low temperatures, while alternative splicing was observed among the *TPO* gene transcripts. The latter led to skipping exons 4 and 5 in the cold environment and the corresponding synthesis of a *TPO* short isoform with multiple open reading frames generated in Bashang Long-tail and Rhode Island Red chickens. This suggested a tentative molecular mechanism underlying cold adaptation and/or acclimation in chickens. How this affects the cold tolerance of chickens, according to the authors [22], requires further investigation. In any event, *TPO* exemplifies, along with *BDNF*, a crucial role of epigenetic control (via methylation, histone modification, and splicing) in regulating genes involved in cold tolerance manifestation. Besides, polymorphisms in the *TPO* gene associated with chicken growth and carcass traits (body weight, breast bone length, pectoral angle, claw weight, and leg muscle weight) were discovered [73].

In the current study, we observed a ROH in USH on GGA3 just at the site where *TPO* is located as well as an *F*_ST_-derived selection footprint for USH and RUW (Table S5). We can suggest *TPO* as a good candidate, however, in this case, we might have a nonidentical genetic mechanism of cold resistance associated with this gene and different from that described by Xie et al. [22]. In their study, differential expression of the *GPD1L* (glycerol-3-phosphate dehydrogenase 1-like) gene was also noted, being increased under normal conditions and decreased at low temperatures. The mechanism of this response is unknown, and what functions this gene has in the thyroid gland remains unclear. In the present investigation, we identified this candidate gene in a ROH region on GGA2 in USH.

In general, our research resulted in a number of other PCGs (e.g., *PTHLH*, *THRSP*, *PTH2R*, *TRH*, *TRHR*, *LOC416924* (*THADA*), *HSP90B1*) associated with biochemical networks and pathways in which thyroid hormones are involved as mediators of cold resistance. Thus, they should also be considered in future studies on chickens in connection with cold adaptation and acclimation.

#### PCGs for thermoregulation and cold tolerance: other genes

Amongst the numerous genes related to phenotypic traits for which the studied breeds could be selected, we were able to find, for example, the following PCGs:

1. ***CRLF1*** **(cytokine receptor like factor 1)**, known for its mutation (deletion) in humans that causes a specific syndrome of body response to cold temperatures, i.e., cold-induced sweating [74]. In our study, this gene is detected in a ROH region in USH and can also be considered as a potential candidate associated with response to low temperatures in chickens.
2. **TRP (transient receptor potential) gene family**. These genes are involved in the operation of cell ion channels. In mammals, these genes were also found to be responsible for cold sensation as well as responses to both cold and hot conditions [74]. We detected some of these genes in ROH regions in USH and OMF including *PKD2*, *TRPM1*, *TRPC7*, and *TRPV2*.
3. ***PPARGC1A*** **(PPARG coactivator 1 alpha) and neighbouring genes**. *PPARGC1A* regulates expression of genes related to adaptive thermogenesis, muscle fibre type differentiation, energy metabolism, and fuel homeostasis [75]. This gene is known to be overexpressed in the skeletal muscles of cold-exposed chickens [76]. In the present investigation, this gene was also revealed in a ROH on GGA4 in USH. Cold exposure in rats resulted in elevated *PPARGC1A* expression in type I (slow-twitch) and type II (fast-twitch) fibres of gastrocnemius muscle and higher glucose uptake [75]. In birds, skeletal muscle shivering and non-shivering thermogenesis regulate body temperature in a cold environment. It was demonstrated that cold tolerance occurs in chicks after their skeletal muscles mature; in 7-day-old and younger chicks, leg muscle fibres transform to the slow-twitch type (type I) and show an increased *PPARGC1A* expression due to 24-h cold exposure causing cold tolerance. This change in response to acute cold might be involved in the increased transformation of fibre type at the initial stage of adaptation in cold-exposed chicks [77].
  Ueda et al. [76] also pointed out that there is a difference in the pectoralis muscle fibres in cold-acclimated chickens (fast-twitch-oxidative (type IIA) fibres) and control chickens (fast-twitch-glycolytic (type IIB) fibres), suggesting a role of muscle fibre specialization in adaptive thermogenesis. There is also a report that no difference was observed in *PPARGC1A* expression in skeletal muscles between cold-sensitive (1-day-old) and cold-tolerant (4-day-old) neonatal chicks [78]. We would suggest that a similar study of fibre types is worthy in the future, e.g., in USH, OMF and RUW chickens, in order to clarify the nature of chicken muscle fibre transformation and role of the *PPARGC1A* gene in adaptive thermogenesis and cold tolerance. In addition, *PPARGC1A* is a candidate gene associated with growth, body weight and muscle mass [79, 80]. It is also involved in abdominal fat deposition [81] and has an important regulatory function to intramuscular fat metabolism deposition [82].
  Two more genes in the above ROH region are *TBC1D1* and *CCKAR*. *TBC1D1* is relevant to controlling energy homeostasis in vertebrates and may play an evolutionary conserved role in this process [83]. Higher *TBC1D1* mRNA levels were reported in cocks compared to hens in the thigh muscle and abdominal fat [84], the gene being also a functional candidate gene for growth performance and fat deposition [85]. *CCKAR* is a key receptor mediating satiety within a GGA4 region harbouring functional variants affecting the growth, reproductive traits, and feed intake [86, 87]. Decreased expression of this satiety signal receptor was linked to increased growth and body weight during the domestication of chickens [88]. It is also expressed in immune organs and cells, being regulated by inflammatory stimuli associated with bacterial and viral infection [89].
  The same region encompasses other important genes including *SLIT2*, *NCAPG*, *LCORL* and *LDB2*. *SLIT2* is involved in small yellow follicle development (a key determinant of chicken reproductive performance) [90] and may regulate body weight, growth, carcass traits and feed conversion [79]. SNPs identified for the *NCAPG* gene are associated with economically important traits (egg and meat productivity, reproduction) [19, 91], with genotypic variability being established between various breeds [19]. *LCORL* is a possible candidate responsible for growth, body weight, slaughter traits and egg performance, with different genotypes being identified in diverse breeds [12, 19, 91, 92]. *LDB2* is a candidate gene for rapid growth in broilers, body weight and carcass traits [57, 92].
4. **The** ***MSTN*** **(*GDF8*) gene encoding myostatin** (GGA7, 0.1.4.6 Mb; in OMF and USH) is also associated with cold tolerance in chickens. Ijiri et al. [77] observed its depressed expression in the leg muscles of 7-day-old and younger chicks within 24 h of cold exposure, which is required for chicks to acquire cold tolerance and results in the increase of skeletal muscle in cold-exposed chicks. The gene expression and polymorphism are also associated with body weight, skeletal muscle and adipose growth, and carcass traits in chicken [93, 94]. Within the same region, we identified the *FN1* gene that might be a key candidate gene for egg production [95] and is associated with immune response [96].

Last but not least, the adaptive features of birds are associated with the formation of a feather covering built from beta-keratins that form a multigene family in the chicken [97–99]. A number of feather keratin clades were identified on GGA27 that form monophyletic groups [97]. In our study, we detected a selective sweep region on GGA27 that contain beta-keratin gene clusters, suggesting their significance for cold adaptation.

Lately, Buggiotti et al. [100] reported a rare instance of amino acid residue alteration shared by at least 16 species of hibernating/cold-adapted mammals, i.e., a Yakut cow breed-specific missense mutation in a highly conserved *NRAP* gene. An occurrence of convergent evolution along the mammalian evolutionary tree was suggested, with rapid fixation in a single isolated population of cattle exposed to a severe Siberian environment. In our investigation, we did not reveal any signal of selective sweep overlapping with the chicken *NRAP* gene located on GGA6. Near this gene, however, we detected an *F*_ST_-based selection signature in USH and OMF as well as a ROH in USH. This might leave a room for speculating about a possible role of *NRAP* or its regulatory elements in convergent adaptation to low temperature in chickens. To test this suggestion, a sequencing of the *NRAP*-containing region would be required in cold tolerant breeds like USH in the future research.

Breeding chickens in the continental climate might be associated with the selective development of appropriate adaptive features in local chicken breeds. In this regard, the effect of stabilizing selection on these breeds may be suggested in favour of transitional forms that can endure under the most typical but opposite environmental conditions, for example, cold vs. heat [74].

#### Other genes of interest

These genes identified in the regions of selection include those involved in lipid metabolism and development of immunity, i.e., related to the mechanisms of adaptation to adverse environmental factors, and determining other economically important traits in chickens. For instance, the *GH* (growth hormone) gene located within a USH-specific ROH on GGA27 is a locus of earlier classical genetic map [6] and may also be associated with MD resistance [101]. This gene was downregulated in broiler liver under chronic thermal stress [102]. There are known polymorphisms in the 3^rd^ intron of the gene that may be potential markers decreasing the abdominal fat traits and increasing growth traits of chickens [103]. *GH* is also associated with egg production, egg weight and growth/meat traits [104]. Another locus of classical genetic map [6], *ACTB* (beta-actin), was included in a USH-specific ROH on GGA14. It is differentially expressed between breeds, being a candidate gene involved with skeletal muscle growth and disease susceptibility in broilers [105], and is also a useful endogenous reference gene for chicken expression studies [64].

The *OVAL* (ovalbumin) gene, a locus of classical genetic map [6], was used for decades as a biochemical polymorphic marker in chickens, though with a rather low degree of polymorphism [106]. To date, the *OVAL* SNPs were identified that are associated with egg quality traits in layers [107]. We found out that this gene overlapped with a USH-specific ROH on GGA2.

For the *POMC* gene we detected in a USH-specific ROH, an overlay with a signal of selective sweep was also reported suggesting its association with traits of economic interest [8]. The *POMC* RNA level in the hypothalamus is responsive to fat-related measures and represents long-term energy status in chickens [108]. SNPs in the gene had potential effects on reproduction traits in chickens [109] and were linked to pelvis breadth, body weight and chest depth [110].

Within a ROH found in USH, there is the *LEPR* (leptin receptor) gene known as a candidate gene suggestive of production-oriented selection [111]. It overlaps with a signal of selective sweep in laying hens [112] and plays an important role in the regulation of reproduction and energy status in Japanese quail [113]. The *LEPR* expression decreased with age in adipose tissue from growing broilers [114], the gene being associated with growth, body weight, and feed efficiency in meat-type chickens [115]. It is also related to effects of stress on immune function in the spleen in a chicken stress model [116].

Among many other PCGs of interest, we identified *RARRES1* that is located in a ROH region on GGA9 (21.4.24.1 Mb) shared between USH and OMF. It is also known as ovocalyxin-32, an eggshell matrix protein associated with eggshell quality and egg production traits (as reviewed in [117]). It is one of the highest expressed genes in the uterus of laying hens [118]. The gene expression in the cecum of layers was also downregulated following phytobiotic administration and upregulated in response to *Salmonella* Enteritidis challenge [64], being negatively correlated with urea and urea nitrogen content in blood of layers and positively correlated with alpha amylase activity [63].

Many detected selective footprints and PCGs overlapped with Chicken QTLdb [46] loci, validating and supporting further significance of our findings in the genomes of the four investigated chicken breeds.

## Conclusion

Based on the SNP genotype data obtained for the four different breeds, we annotated here the entire array of genes (~ 4000 genes) found in the regions under selective pressure and chose 540 PCGs from this number. Priority was given to candidate genes associated with adaptation to cold or temperature impacts in general, phenotypic traits that contribute to adaptation to environmental factors (plumage, comb, immunity, etc.), as well as traits of egg and meat performance. These PCGs were additionally assigned to a specific breed, considering the methods used to determine selective sweep signals. As a result, a clear insight was obtained about which genes and in which breeds (out of the four studied) were subjected to putative selection for certain traits.

Cold tolerance in acclimated chicken breeds might be generated through one of a few unique gene expression networks or multiple overlapping responses that have been observed in cold-exposed animals. Role of epigenetic factors can also be important in regulating cold tolerance-related genes. This information will serve as the basis for a more complete understanding of the mechanisms of adaptation and acclimation in chickens and for further, more detailed study of the genes underlying phenotypic traits of interest. Analyses of the relationship between cold adaptation phenotype and genotypes using a segregating population from more than two breeds, QTL mapping/GWAS approaches can be planned for future research to find the direct evidence of candidate genes for cold adaptation.

## Supplementary Information

**Additional file 1: Table S1.** Major classical phenotypic characteristics and mutations in the Russian White (RUW), White Cornish (WCR), Ushanka (USH), and Orloff Mille Fleur (OMF) breeds studied.

**Additional file 2: Fig. S1.** Cross-validation (CV) error (*Y*-axis) for different K-values (*X*-axis). **Fig. S2.** Admixture bar plot at K = 2, 3, and 8 (the most probable number of ancestral populations) for the four chicken breeds studied: OMF, Orloff Mille Fleur; RUW, Russian White; USH, Ushanka; WCR, White Cornish.

**Additional file 3: Fig. S3.** Distribution of ROH islands among chromosomes.

**Additional file 4: Fig. S4.** Distribution of SNPs by *F*_ST_ value at pairwise comparison of breeds: A USH/OMF, B USH/RUW, and C USH/WCR. Breeds: OMF, Orloff Mille Fleur; RUW, Russian White; USH, Ushanka; WCR, White Cornish.

**Additional file 5: Table S2.** Distribution of ROHs by mean length (sheet A) and number (sheet B) among length classes.

**Additional file 6: Table S3.** Distribution of ROH islands by breeds (sheet A) and by chromosomes (sheet B).

**Additional file 7: Table S4. ***F*_ST_ values for the top 0.1% SNPs at pairwise comparison of the studied breeds: by pairs of breeds (sheet A), and by localization (sheet B).

**Additional file 8: Table S5.** Selection sweeps identified in the genomes of the studied chicken breeds by three different statistics.

**Additional file 9: Table S6.** Summary of chicken genes and their human orthologs within genomic regions of selective sweeps as retrieved from BioMart (Ensembl Genes release 106).

**Additional file 10: Table S7.** Prioritized genes under selection distributed by chromosomes. **Table S8.** Prioritized genes under selection distributed by phenotypic category. **Table S9.** Prioritized genes under selection distributed by breed.

**Additional file 11: Fig. S5.** Venn diagrams representing distribution of 540 prioritized genes between the four studied breeds by phenotypic categories (A to L).

**Additional file 12: Table S10.** Phenotypic traits linked to known QTLs and associated genes within selective sweep regions identified in the genomes of the four chicken breeds.

## Data Availability

All data generated or analysed during the present study are available from the corresponding author on reasonable request. The datasets supporting the conclusions of this article are included in the main manuscript and supplemental materials.

## Abbreviations

*A*_*R*_: Rarefied allelic richness
bp: Base pair
FRCARPRTI: Federal Research Centre “All-Russian Poultry Research and Technological Institute”
*F*_ROH_: Genomic inbreeding coefficient inferred from mean ROH lengths
*F*_ST_: Fixation index, a measure of genetic differentiation
Gb: Gigabases
GGA: *Gallus gallus*, Latin name for chicken used to designate chicken chromosomes
GRCg6a: Genome Reference Consortium build 6a for *Gallus gallus* (chicken)
h: Hour
*H*_*O*_: Observed heterozygosity
K: Number of clusters (ancestral populations)
kb: Kilobases
LKEFRCAH: L. K. Ernst Federal Research Centre for Animal Husbandry
M: Mean value
Mb: Megabases
MD: Marek’s disease
OMF: Orloff Mille Fleur
PC: Principal component
PCA: Principal component analysis
PCG: Prioritized candidate gene
PSG: Positively selected gene
QTL: Quantitative trait locus
ROH: Run of homozygosity
RRIFAGB: Russian Research Institute of Farm Animal Genetics and Breeding
RUW: Russian White
SE: Standard error
SNP: Single nucleotide polymorphism
T_3_: Triiodothyronine
_*U*_*F*_IS_: _*U*_*H*_*E*_-based inbreeding coefficient
_*U*_*H*_*E*_: Unbiased expected heterozygosity
USH: Ushanka
WCR: White Cornish
