# Supplementary material for: Selective footprints and genes relevant to cold adaptation and other phenotypic traits are unscrambled in the genomes of divergently selected chicken breeds
Source: J Anim Sci Biotechnol. 2023 Feb 24;14:35. doi: 10.1186/s40104-022-00813-0 (PMC9951459; doi:10.1186/s40104-022-00813-0)
Supplement: Supplementary file 1 — Additional file 1: Table S1. Major classical phenotypic characteristics and mutations in the Russian White (RUW), White Cornish (WCR), Ushanka (USH), and Orloff Mille Fleur (OMF) breeds studied. [file 40104_2022_813_MOESM1_ESM.docx]

**Additional file 1: Table S1.** Major classical phenotypic characteristics and mutations in the Russian White (RUW), White Cornish (WCR), Ushanka (USH), and Orloff Mille Fleur (OMF) breeds studied

| **Breed** | **Classical character (genotype)^1^** | **Underlying gene (symbol)** | **Chromosome** | **Refs** |
| --- | --- | --- | --- | --- |
| RUW | dominant white plumage (*I*/*I)* | premelanosome protein (*PMEL*) or, alternatively, erb-b2 receptor tyrosine kinase 3 (*ERBB3*) | GGA34 | [2–5] (*PMEL*), [6] (*ERBB3*) |
|  | single comb (*r*^+^/*r*^+^) | homeodomain protein (*MNR2*) | GGA7 | [7] |
| WCR | recessive white plumage (*c*/*c*) | tyrosinase (*TYR*) | GGA1 | [3–5,8] |
|  | pea comb (*P*/*P*, *r*^+^/*r*^+^) | SRY-box 5 (*SOX5*), homeodomain protein (*MNR2*) | GGA1 (*SOX5*), GGA7 (*MNR2*) | [9] (*SOX5*), [7] (*MNR2*) |
| USH | rose (*R*/*R*), sometimes single comb (*r*^+^/*r*^+^) | homeodomain protein (*MNR2*) | GGA7 | [7] |
|  | muffs and beard (*Mb*/*Mb*) | homeobox B8 (*HOXB8*) | GGA27 | [10] |
|  | extended black (*E*/*E*) and, supposedly, melanotic (*Ml*/*Ml*) | melanocortin 1 receptor (*MC1R*) for *E*, gap junction protein alpha 5 (*GJA5*) for *Ml* | GGA11 (*MC1R*), GGA1 (*GJA5*) | [3–5,11,12] (*MC1R*), [13] (*GJA5*) |
|  | dermal melanin inhibitor (*id*^+^/*id*^+^ or *id*^+^/*–*) | beta-1,4-galactosyltransferase 1 (*B4GALT1*) or versican (*VCAN*); alternatively, GRAM domain containing 3 (*GRAMD3*) | GGAZ | [3–5,14] (*B4GALT1* or *VCAN*), [15,16] (*GRAMD3*) |
| OMF | late feathering (*K*/*K* or *K*/–) | endogenous retrovirus (*EV21*); reassigned to prolactin receptor (*PRLR*) and sperm flagellar 2 (*SPEF2*) | GGAZ | [3–5,17] (*EV21*), [18] (*PRLR*/*SPEF2*) |
|  | muffs and beard (*Mb*/*Mb*) | homeobox B8 (*HOXB8*) | GGA27 | [10] |
|  | walnut comb (*P*/*P*, *R*/*R*) | SRY-box 5 (*SOX5*), homeodomain protein (*MNR2*) | GGA1 (*SOX5*), GGA7 (*MNR2*) | [9] (*SOX5*), [7] (*MNR2*) |
|  | mille fleur color phase (*e^b^*/*e^b^*, *Co*/*Co*, *mo*/*mo*) | melanocortin 1 receptor (*MC1R*) for *e^b^*, endothelin receptor B subtype 2 (*EDNRB2*) for *mo*, other genes for *Co* | GGA11 (*MC1R*), GGA4 (*EDNRB2*) | [3–5,11,12] (*MC1R*), [19] (*EDNRB2*), [20] (other genes) |

^1^ As reviewed in [1].

**References**

1. Somes RG, Jr. International registry of poultry genetic stocks. Bull Storrs Agric Exp Stn. Storrs: University of Connecticut Publication; 1988. No. 476.
2. Kerje S, Sharma P, Gunnarsson U, Kim H, Bagchi S, Fredriksson R, et al. The *Dominant white*, *Dun* and *Smoky* color variants in chicken are associated with insertion/deletion polymorphisms in the *PMEL17* gene. Genetics. 2004;168(3):1507–18. https://doi.org/10.1534/genetics.104.027995.
3. Romanov MN, Sazanov AA, Smirnov AF. First century of chicken gene study and mapping – a look back and forward. Worlds Poult Sci J. 2004;60(1):19–41. https://doi.org/10.1079/WPS20032.
4. Romanov MN, Sazanov AA, Moiseyeva IG, Smirnov AF. Poultry. In: Cockett NE, Kole C, editors. Genome mapping and genomics in animals, Vol. 3: Genome mapping and genomics in domestic animals. Berlin–Heidelberg–New York: Springer-Verlag; 2009. p. 75–141. https://doi.org/10.1007/978-3-540-73835-0_5.
5. Moiseyeva IG, Romanov MN, Nikiforov AA, Avrutskaia TB. Studies in chicken genetics. Commemorating the 120th anniversary of the outstanding Soviet geneticist A. S. Serebrovsky (1892–1948). Russ J Genet. 2012;48: 869–85. https://doi.org/10.1134/S1022795412090074.
6. Guo Y, Ou JH, Zan Y, Wang Y, Li H, Zhu C, et al. Researching on the fine structure and admixture of the worldwide chicken population reveal connections between populations and important events in breeding history. Evol Appl. 2021;15(4):553–64. https://doi.org/10.1111/eva.13241.
7. Imsland F, Feng C, Boije H, Bed'hom B, Fillon V, Dorshorst B, et al. The Rose-comb mutation in chickens constitutes a structural rearrangement causing both altered comb morphology and defective sperm motility. PLoS Genet. 2012;8(6):e1002775. https://doi.org/10.1371/journal.pgen.1002775.
8. Chang CM, Coville JL, Coquerelle G, Gourichon D, Oulmouden A, Tixier-Boichard M. Complete association between a retroviral insertion in the tyrosinase gene and the recessive white mutation in chickens. BMC Genomics. 2006;7:19. https://doi.org/10.1186/1471-2164-7-19.
9. Wright D, Boije H, Meadows JR, Bed'hom B, Gourichon D, Vieaud A, et al. Copy number variation in intron 1 of *SOX5* causes the Pea-comb phenotype in chickens. PLoS Genet. 2009;5(6):e1000512. https://doi.org/10.1371/journal.pgen.1000512.
10. Guo Y, Gu X, Sheng Z, Wang Y, Luo C, Liu R, et al. A complex structural variation on chromosome 27 leads to the ectopic expression of *HOXB8* and the Muffs and beard phenotype in chickens. PLoS Genet. 2016;12(6):e1006071. https://doi.org/10.1371/journal.pgen.1006071.
11. Ling MK, Lagerström MC, Fredriksson R, Okimoto R, Mundy NI, Takeuchi S, et al. Association of feather colour with constitutively active melanocortin 1 receptors in chicken. Eur J Biochem. 2003;270(7):1441–9. https://doi.org/10.1046/j.1432-1033.2003.03506.x.
12. Kerje S, Lind J, Schütz K, Jensen P, Andersson L. Melanocortin 1-receptor (*MC1R*) mutations are associated with plumage colour in chicken. Anim Genet. 2003;34(4):241–8. https://doi.org/10.1046/j.1365-2052.2003.00991.x.
13. Li J, Lee MO, Chen J, Davis BW, Dorshorst BJ, Siegel PB, et al. *Cis*-acting mutation affecting *GJA5* transcription is underlying the *Melanotic* within-feather pigmentation pattern in chickens. Proc Natl Acad Sci U S A. 2021;118(41):e2109363118. https://doi.org/10.1073/pnas.2109363118.
14. Dorshorst B, Okimoto R, Ashwell C. Genomic regions associated with dermal hyperpigmentation, polydactyly and other morphological traits in the Silkie chicken. J Hered. 2010;101(3):339–50. https://doi.org/10.1093/jhered/esp120.
15. Li G, Li D, Yang N, Qu L, Hou Z, Zheng J, et al. A genome-wide association study identifies novel single nucleotide polymorphisms associated with dermal shank pigmentation in chickens. Poult Sci. 2014;93(12):2983–7. https://doi.org/10.3382/ps.2014-04164.
16. Xu J, Lin S, Gao X, Nie Q, Luo Q, Zhang X. Mapping of *Id* locus for dermal shank melanin in a Chinese indigenous chicken breed. J Genet. 2017;96(6):977–83. https://doi.org/10.1007/s12041-017-0862-z.
17. Bacon LD, Smith E, Crittenden LB, Havenstein GB. Association of the slow feathering (*K*) and an endogenous viral (*ev*21) gene on the Z chromosome of chickens. Poult Sci. 1988;67(2):191–7. https://doi.org/10.3382/ps.0670191.
18. Elferink MG, Vallée AA, Jungerius AP, Crooijmans RP, Groenen MA. Partial duplication of the *PRLR* and *SPEF2* genes at the late feathering locus in chicken. BMC Genomics. 2008;9:391. https://doi.org/10.1186/1471-2164-9-391.
19. Kinoshita K, Akiyama T, Mizutani M, Shinomiya A, Ishikawa A, Younis HH, et al. Endothelin receptor B2 (*EDNRB2*) is responsible for the tyrosinase-independent recessive white (*mo^w^*) and mottled (*mo*) plumage phenotypes in the chicken. PLoS One. 2014;9(1):e86361. https://doi.org/10.1371/journal.pone.0086361.
20. Wang X, Li D, Song S, Zhang Y, Li Y, Wang X, et al. Combined transcriptomics and proteomics forecast analysis for potential genes regulating the Columbian plumage color in chickens. PLoS One. 2019;14(11):e0210850. https://doi.org/10.1371/journal.pone.0210850.
