# Supplementary material for: Selective footprints and genes relevant to cold adaptation and other phenotypic traits are unscrambled in the genomes of divergently selected chicken breeds
Source: J Anim Sci Biotechnol. 2023 Feb 24;14:35. doi: 10.1186/s40104-022-00813-0 (PMC9951459; doi:10.1186/s40104-022-00813-0)
Supplement: Supplementary file 2 — Additional file 2: Fig. S1. Cross-validation (CV) error (Y-axis) for different K-values (X-axis). Fig. S2. Admixture bar plot at K = 2, 3, and 8 (the most probable number of ancestral populations) for the four chicken breeds studied: OMF, Orloff Mille Fleur; RUW, Russian White; USH, Ushanka; WCR, White Cornish. [file 40104_2022_813_MOESM2_ESM.docx]

**Additional file 2**

**Fig. S1** Cross-validation (CV) error (*Y*-axis) for different K-values (*X*-axis)

**Fig. S2** Admixture bar plot at K = 2, 3, and 8 (the most probable number of ancestral populations) for the four chicken breeds studied: OMF, Orloff Mille Fleur; RUW, Russian White; USH, Ushanka; WCR, White Cornish
