# Supplementary material for: Selective footprints and genes relevant to cold adaptation and other phenotypic traits are unscrambled in the genomes of divergently selected chicken breeds
Source: J Anim Sci Biotechnol. 2023 Feb 24;14:35. doi: 10.1186/s40104-022-00813-0 (PMC9951459; doi:10.1186/s40104-022-00813-0)
Supplement: Supplementary file 3 — Additional file 3: Fig. S3. Distribution of ROH islands among chromosomes. [file 40104_2022_813_MOESM3_ESM.pdf]

Additional file 3
Fig. S3. Distribution of ROH islands among chromosomes

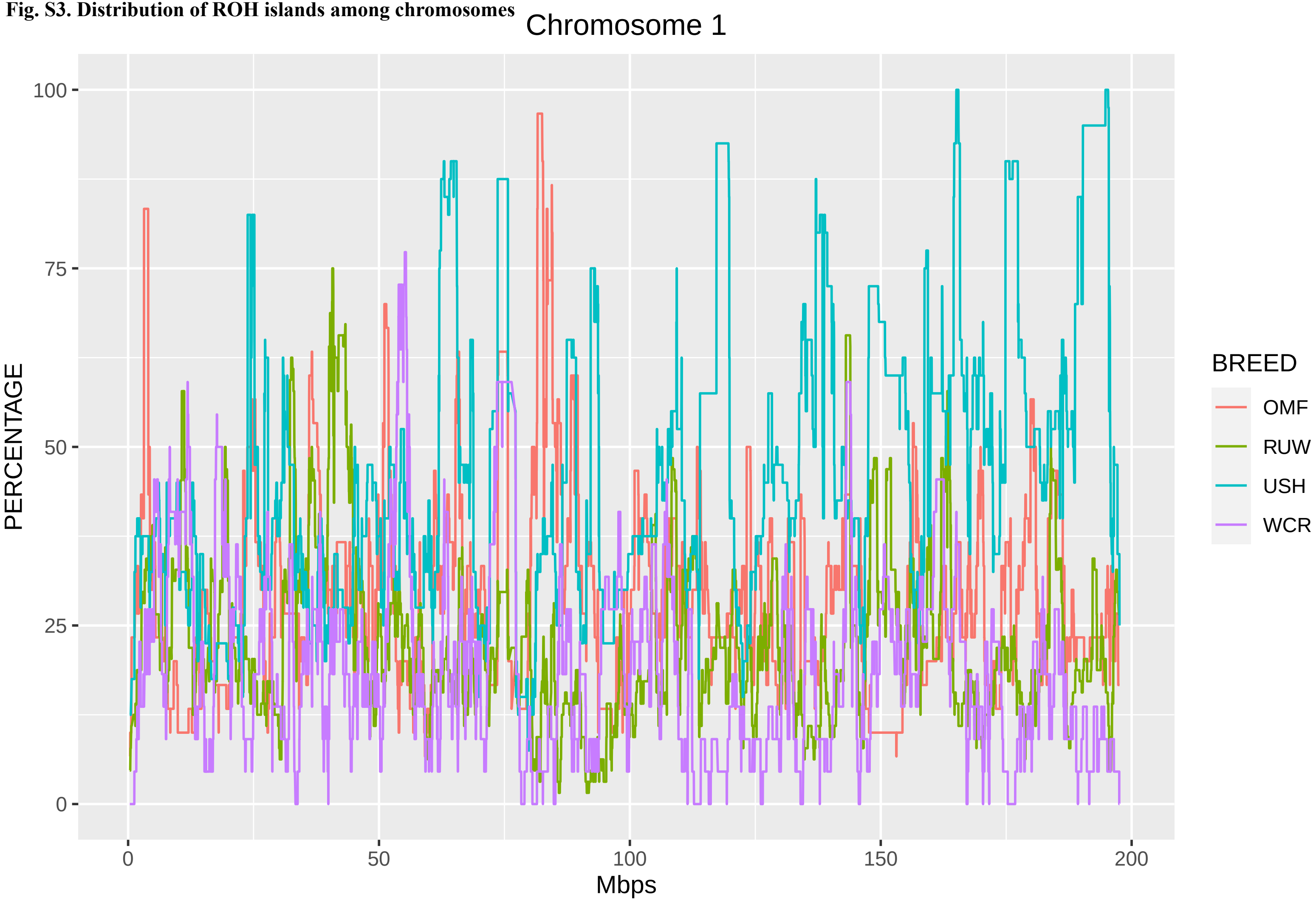

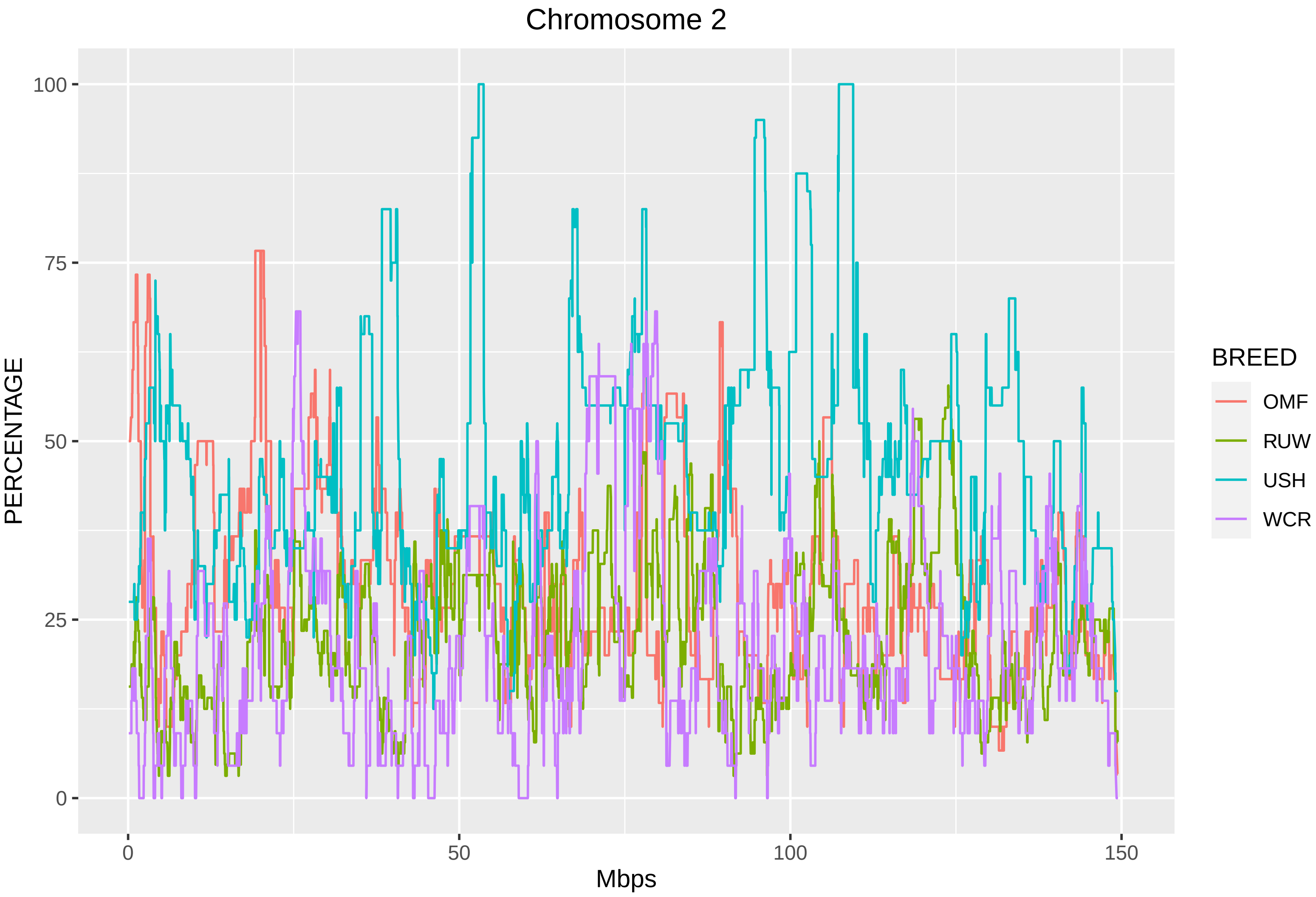
Chromosome 2
100
75
50
25
0
PERCENTAGE
0
50
100
150
Mbps
BREED
OMF
RUW
USH
WCR

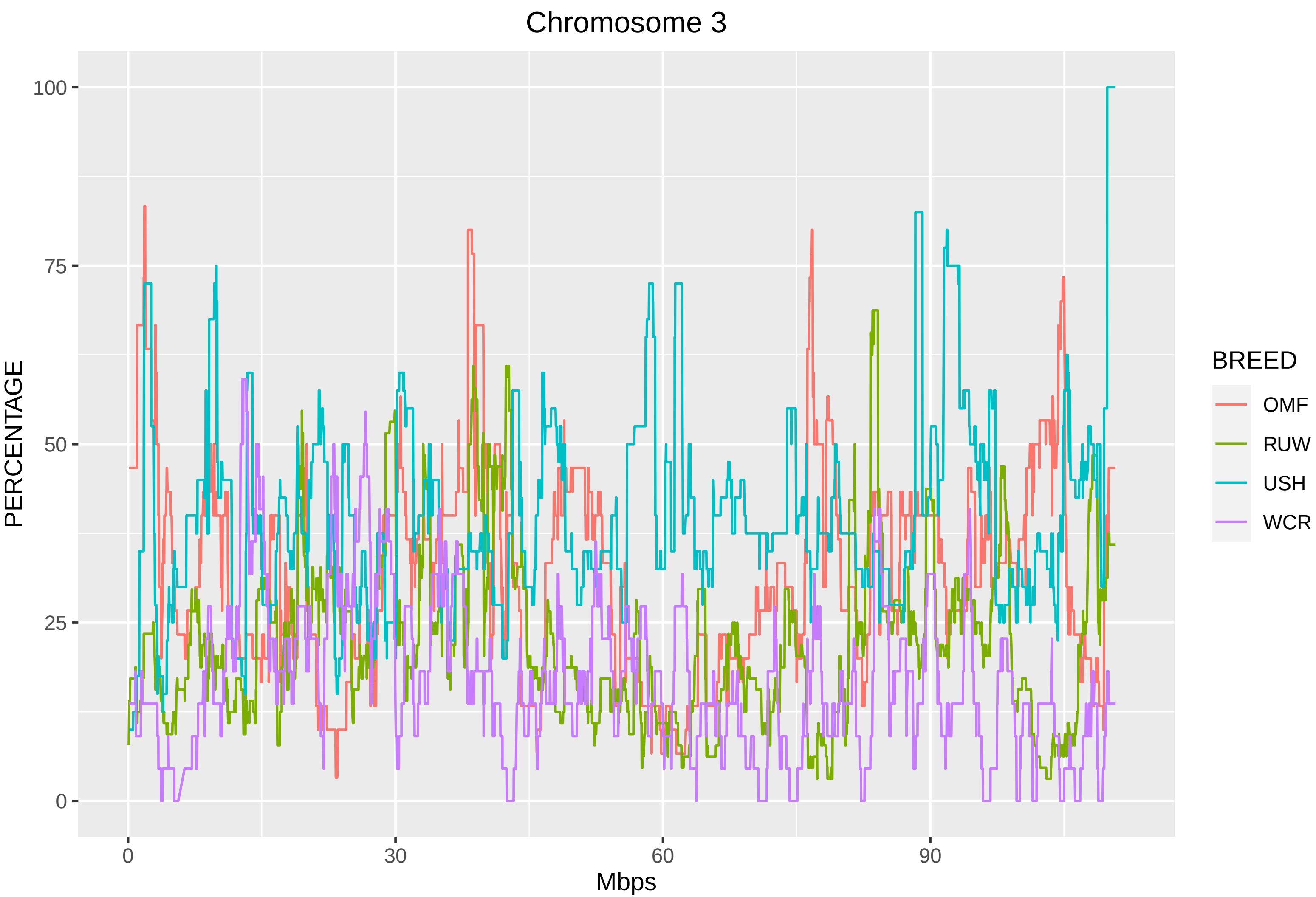
Chromosome 3
PERCENTAGE
0
25
50
75
100
0
30
60
90
Mbps
BREED
OMF
RUW
USH
WCR

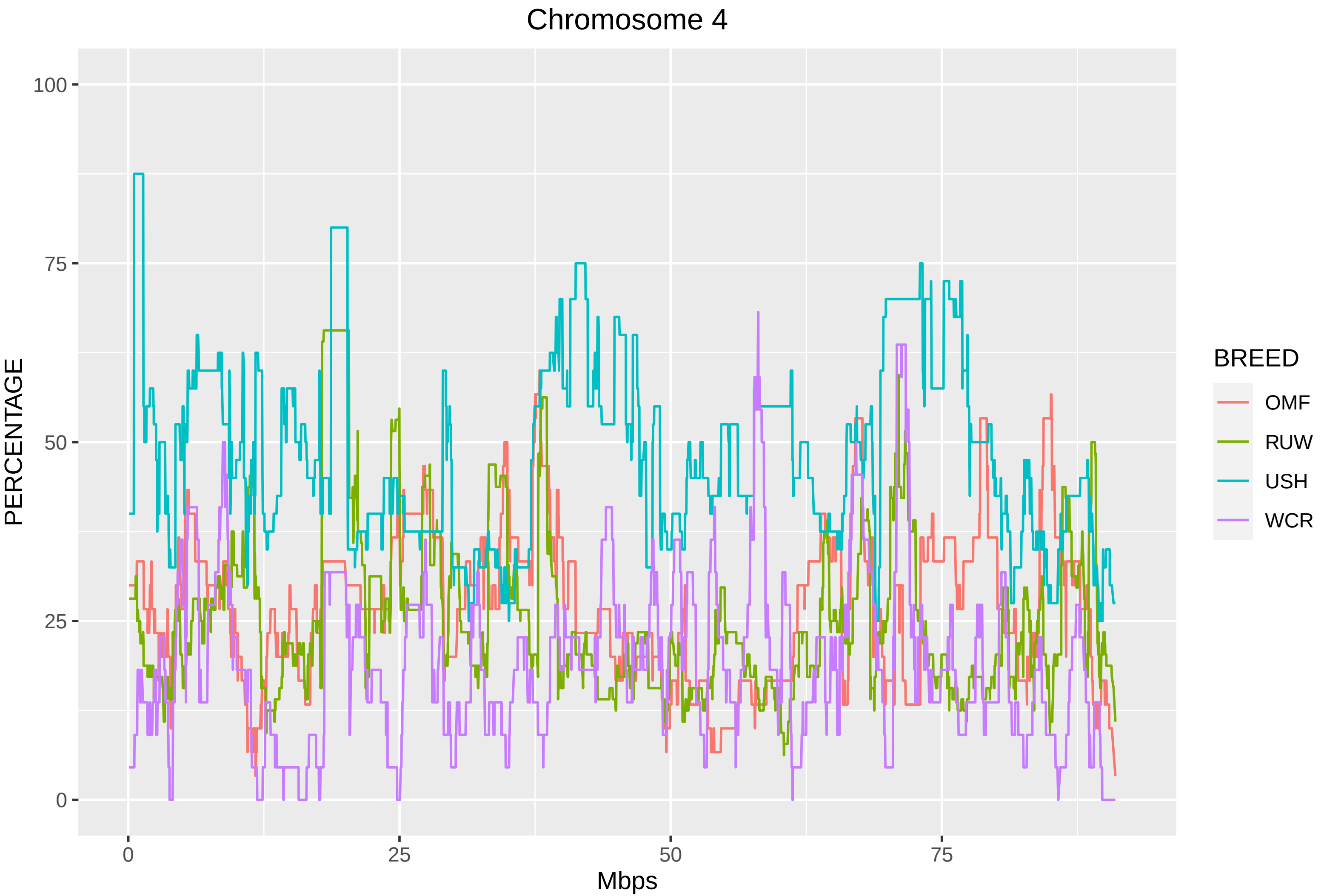
Chromosome 4
PERCENTAGE
100
75
50
25
0
0
25
50
75
Mbps
BREED
OMF
RUW
USH
WCR

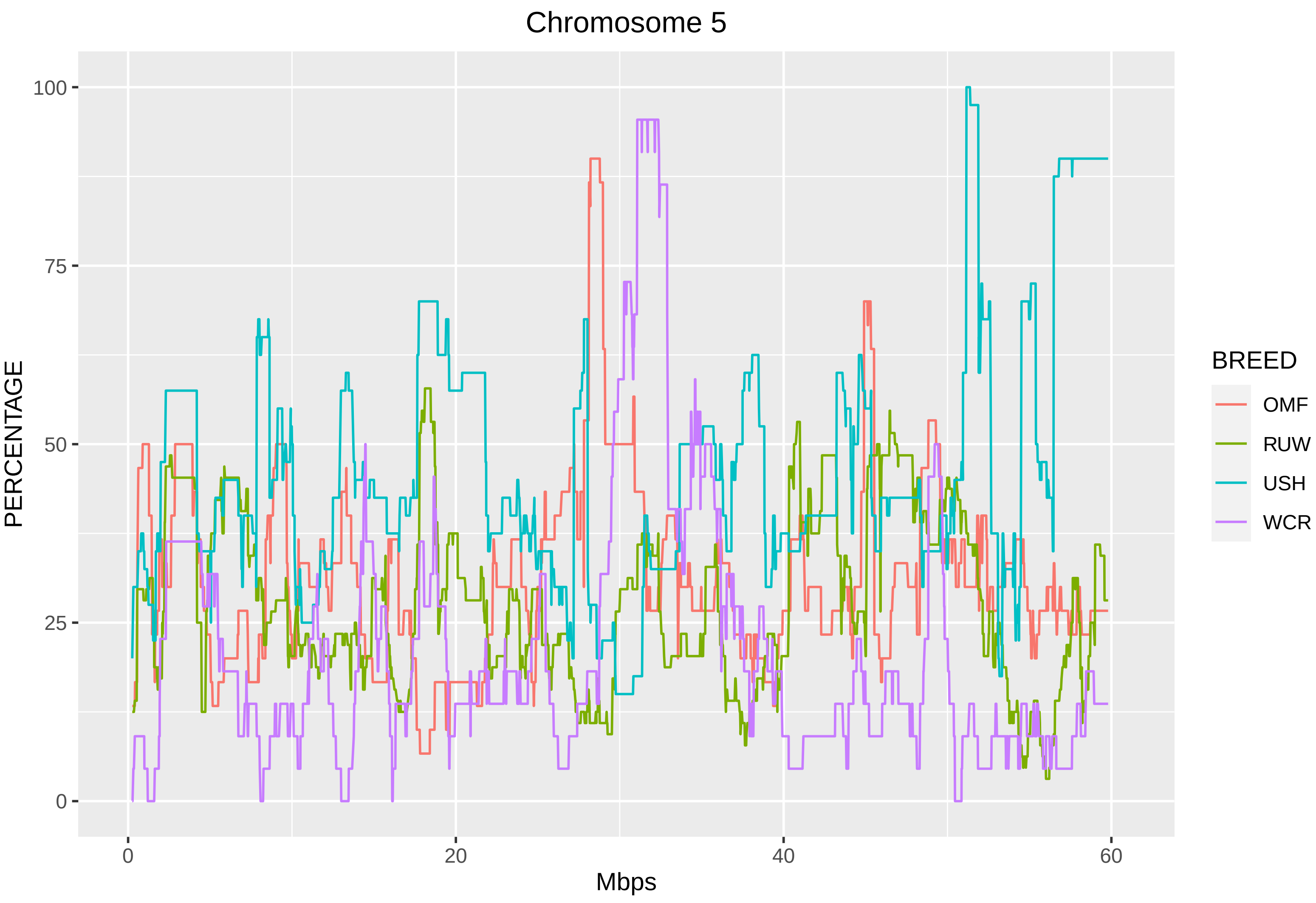
Chromosome 5
PERCENTAGE
100
75
50
25
0
0
20
40
60
Mbps
BREED
OMF
RUW
USH
WCR

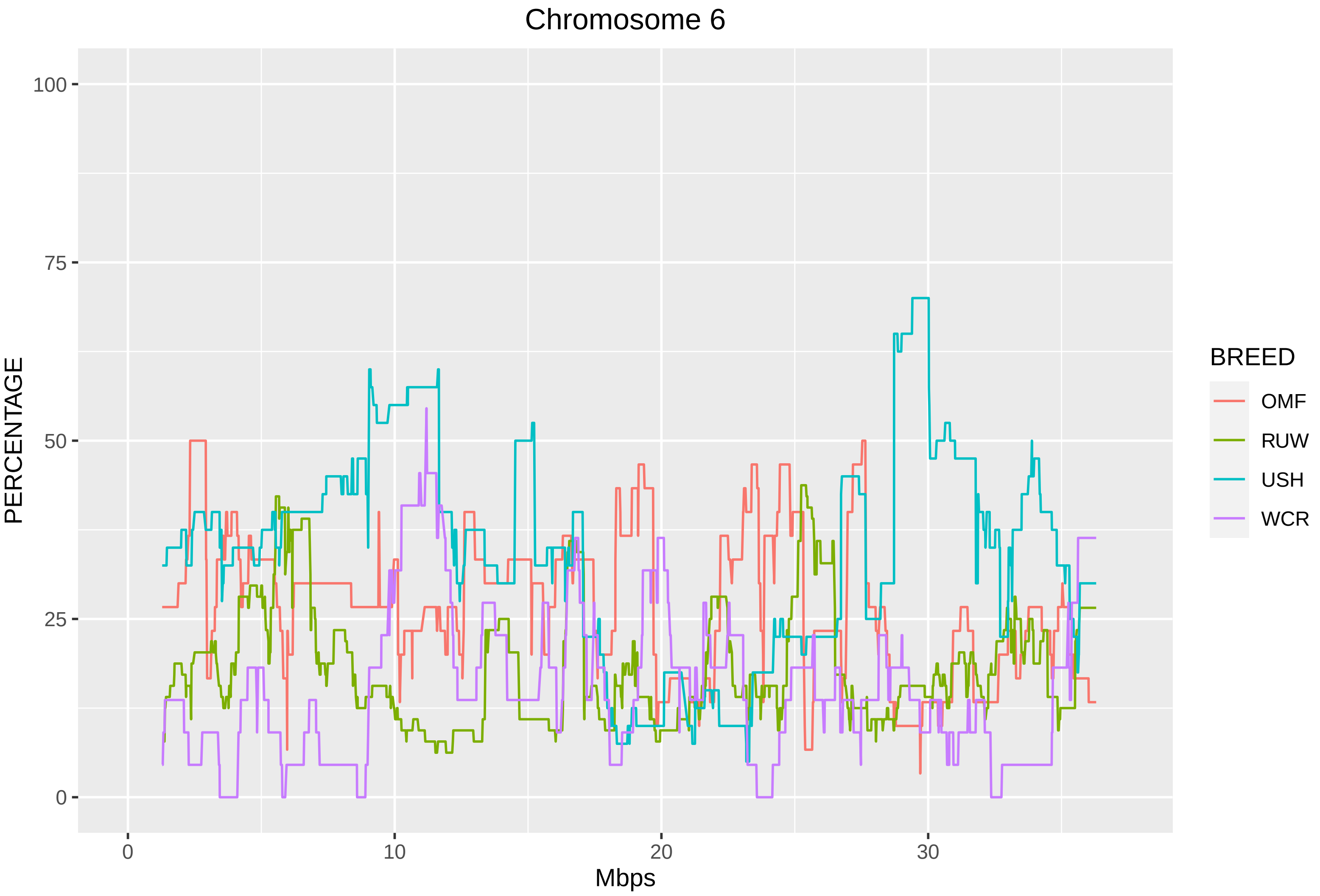
Chromosome 6
PERCENTAGE
100
75
50
25
0
0
10
20
30
Mbps
BREED
OMF
RUW
USH
WCR

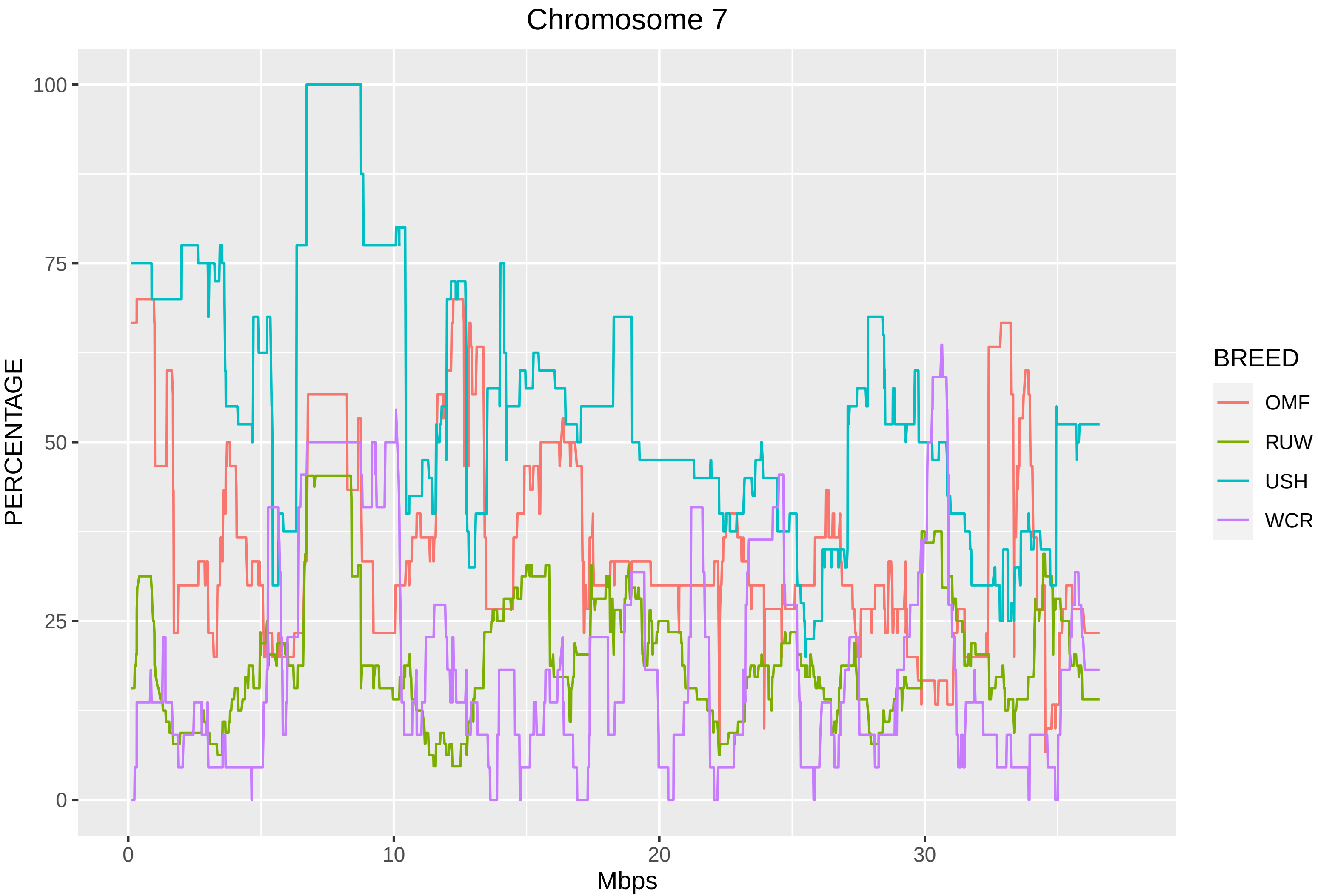
Chromosome 7
PERCENTAGE
100
75
50
25
0
0
10
20
30
Mbps
BREED
OMF
RUW
USH
WCR

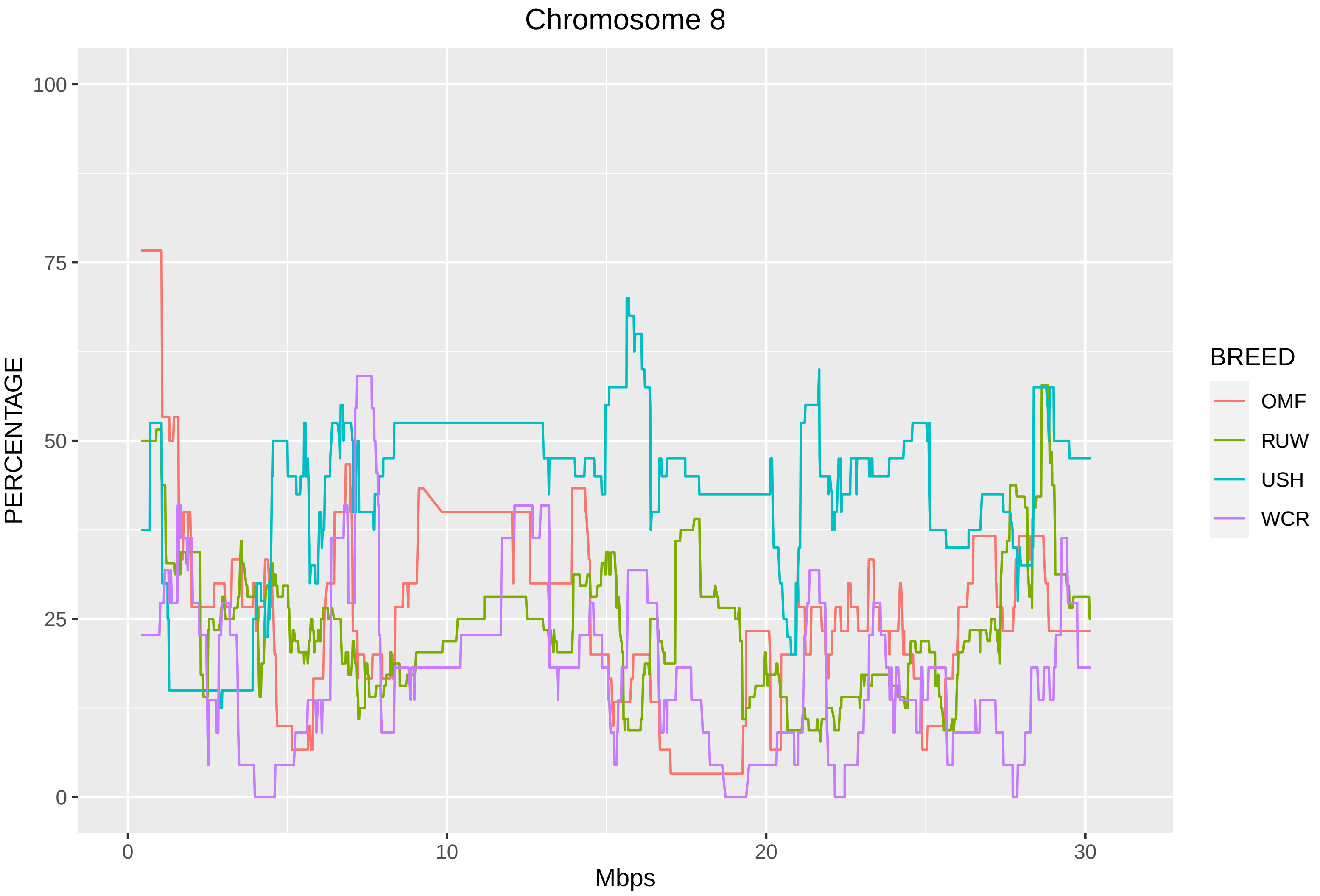
Chromosome 8
PERCENTAGE
100
75
50
25
0
0
10
20
30
Mbps
BREED
OMF
RUW
USH
WCR

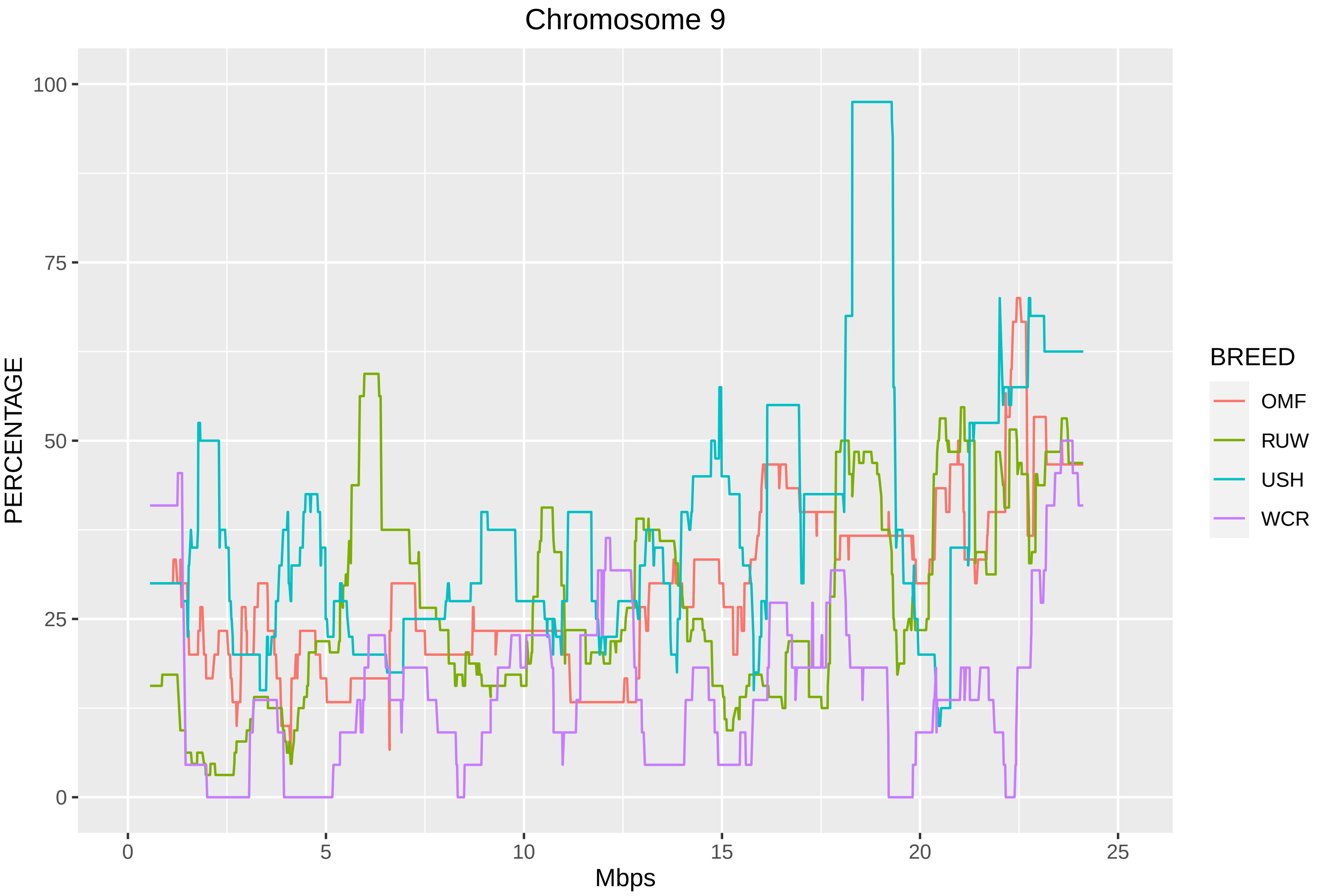
Chromosome 9
PERCENTAGE
100
75
50
25
0
0
5
10
15
20
25
Mbps
BREED
OMF
RUW
USH
WCR

Chromosome 10

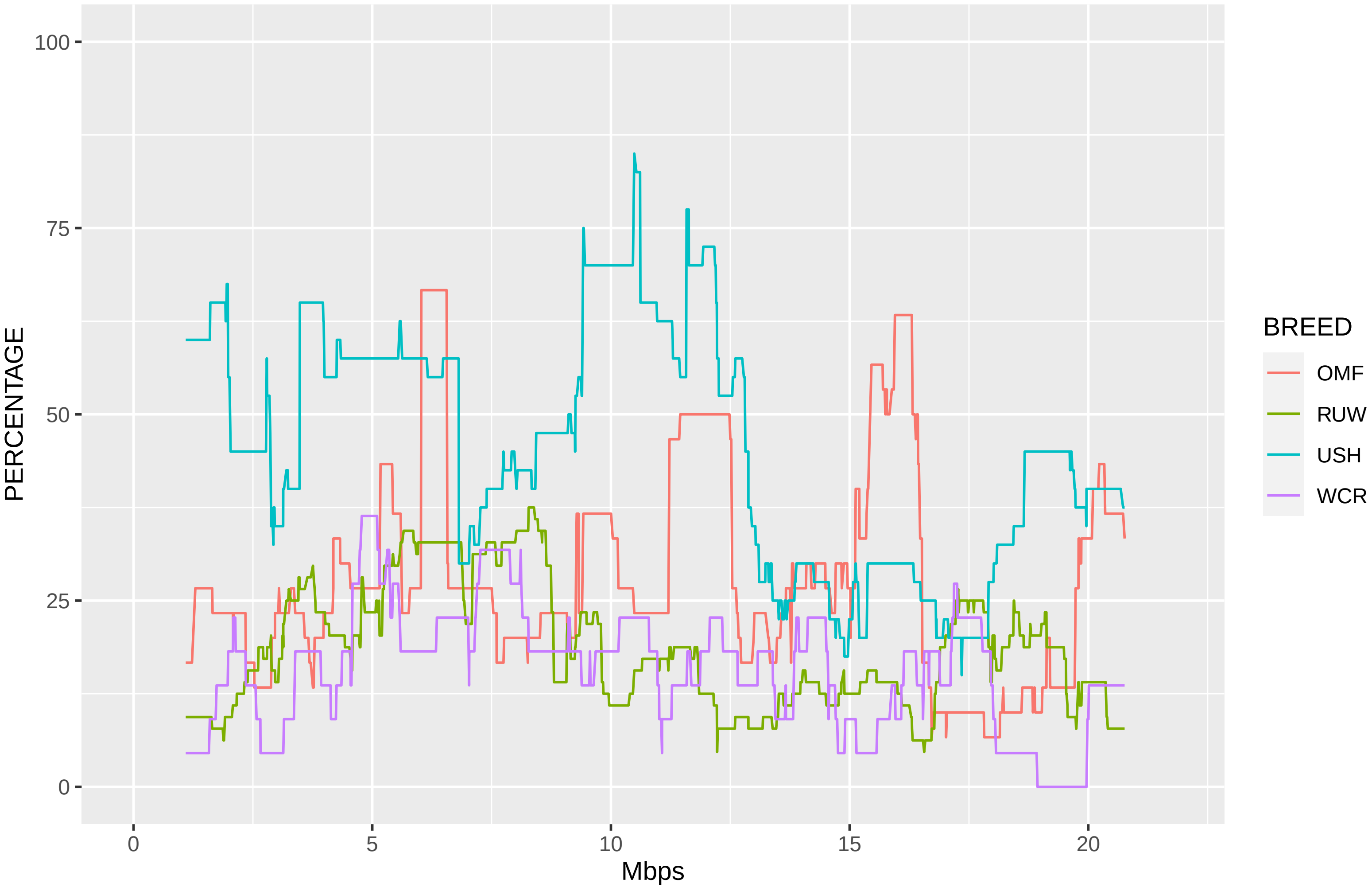

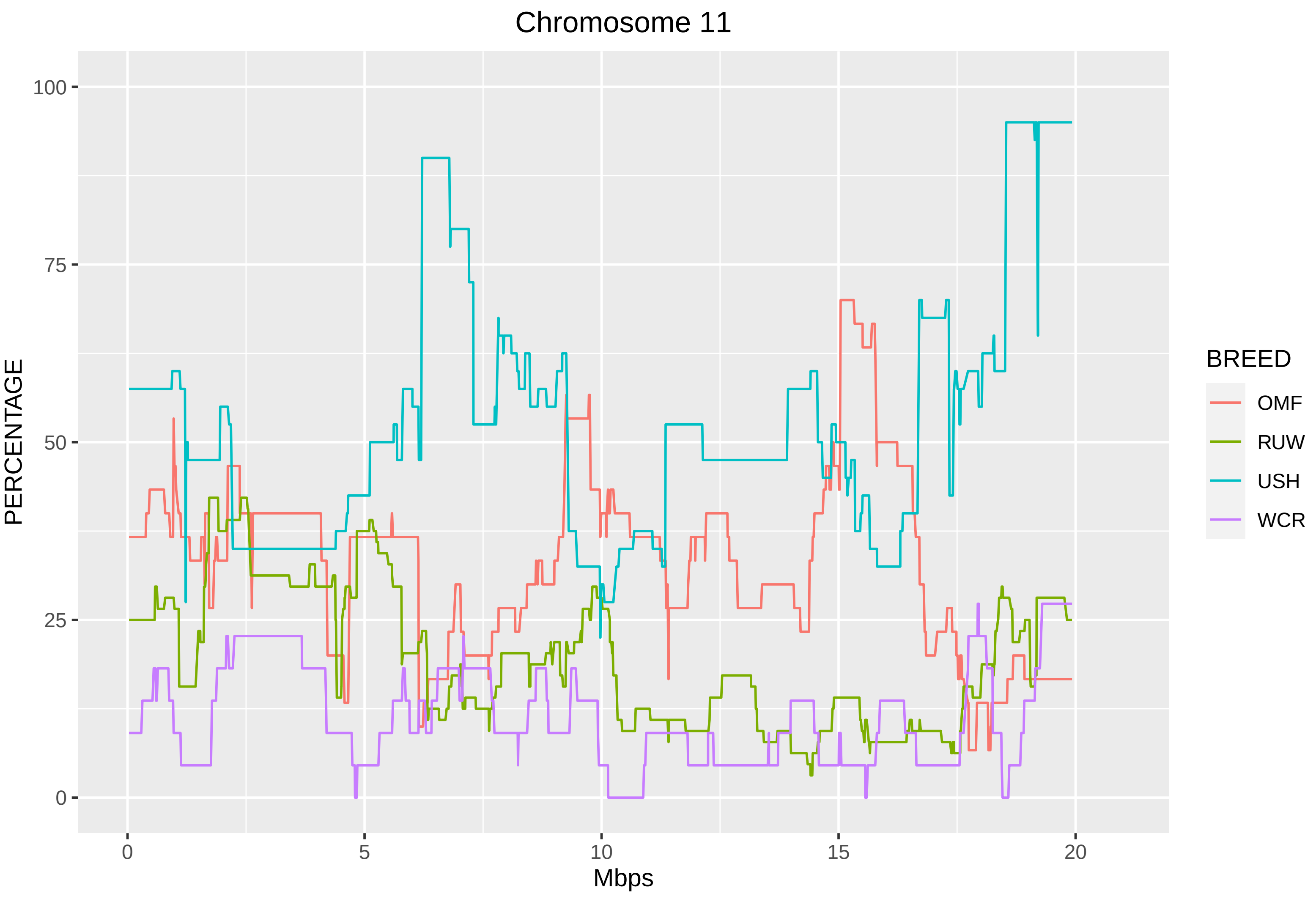

Chromosome 11
PERCENTAGE
100
75
50
25
0
0
5
10
15
20
Mbps
BREED
OMF
RUW
USH
WCR

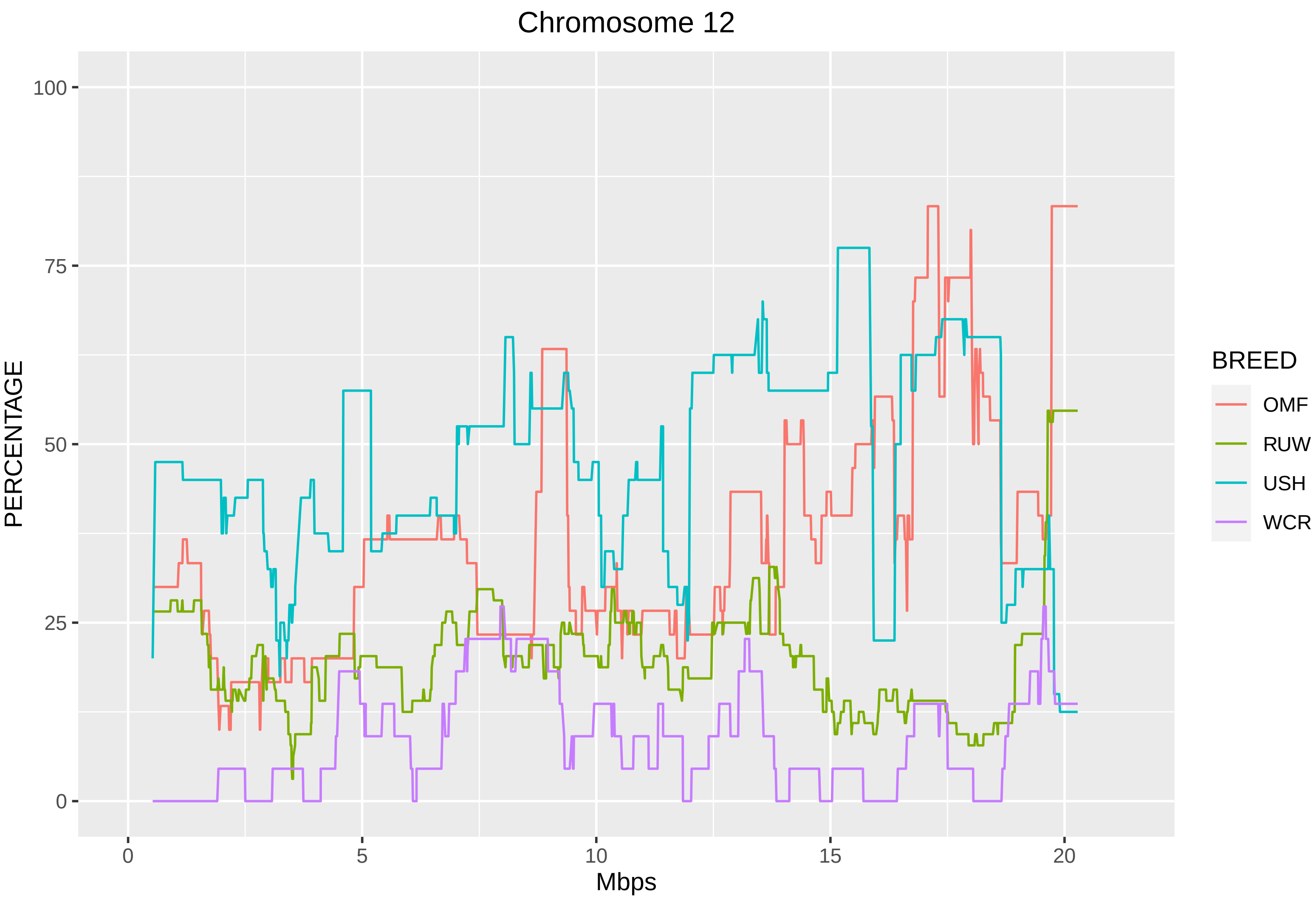
Chromosome 12
PERCENTAGE
100
75
50
25
0
0
5
10
15
20
Mbps
BREED
OMF
RUW
USH
WCR

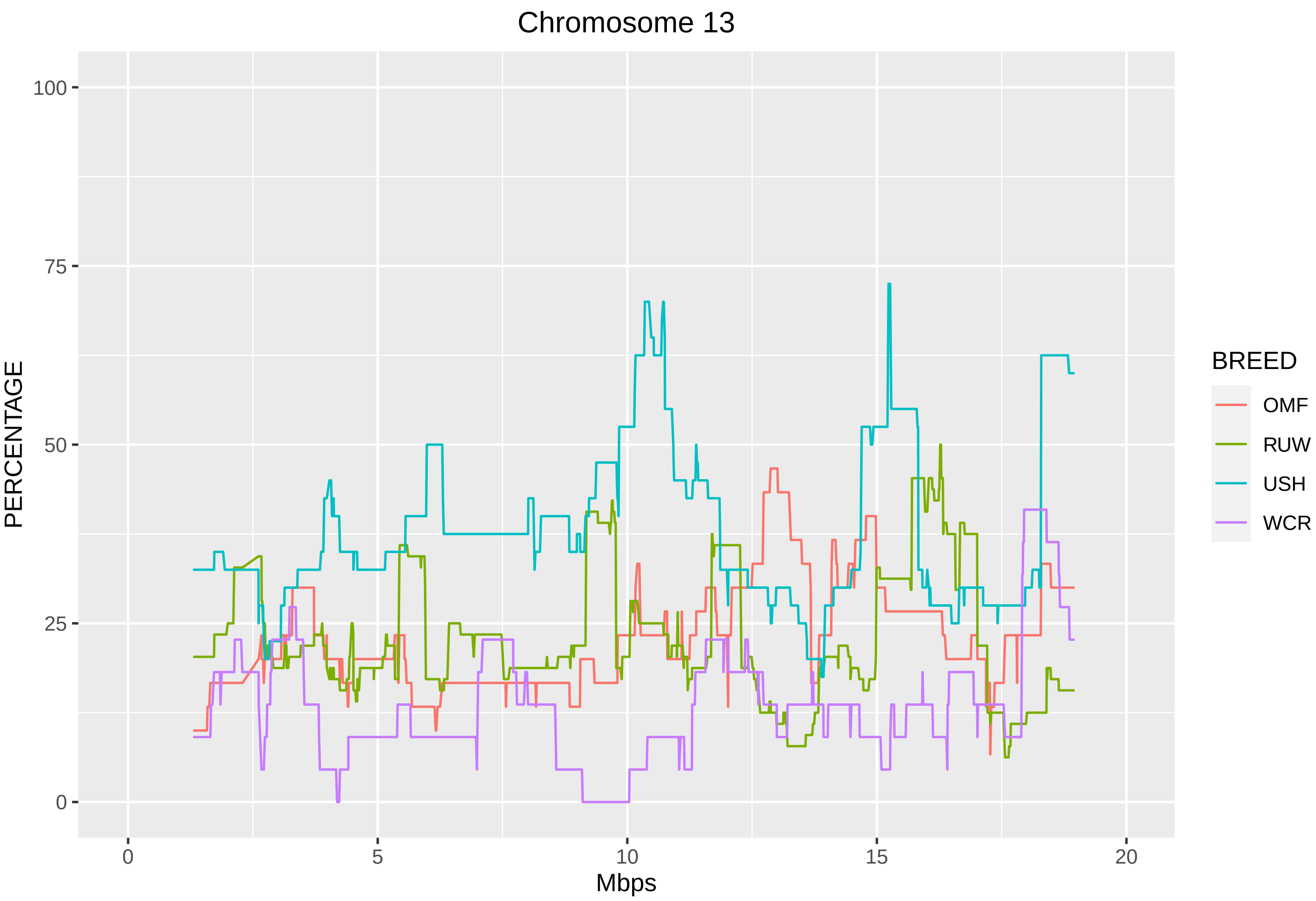
Chromosome 13
PERCENTAGE
100
75
50
25
0
0
5
10
15
20
Mbps
BREED
OMF
RUW
USH
WCR

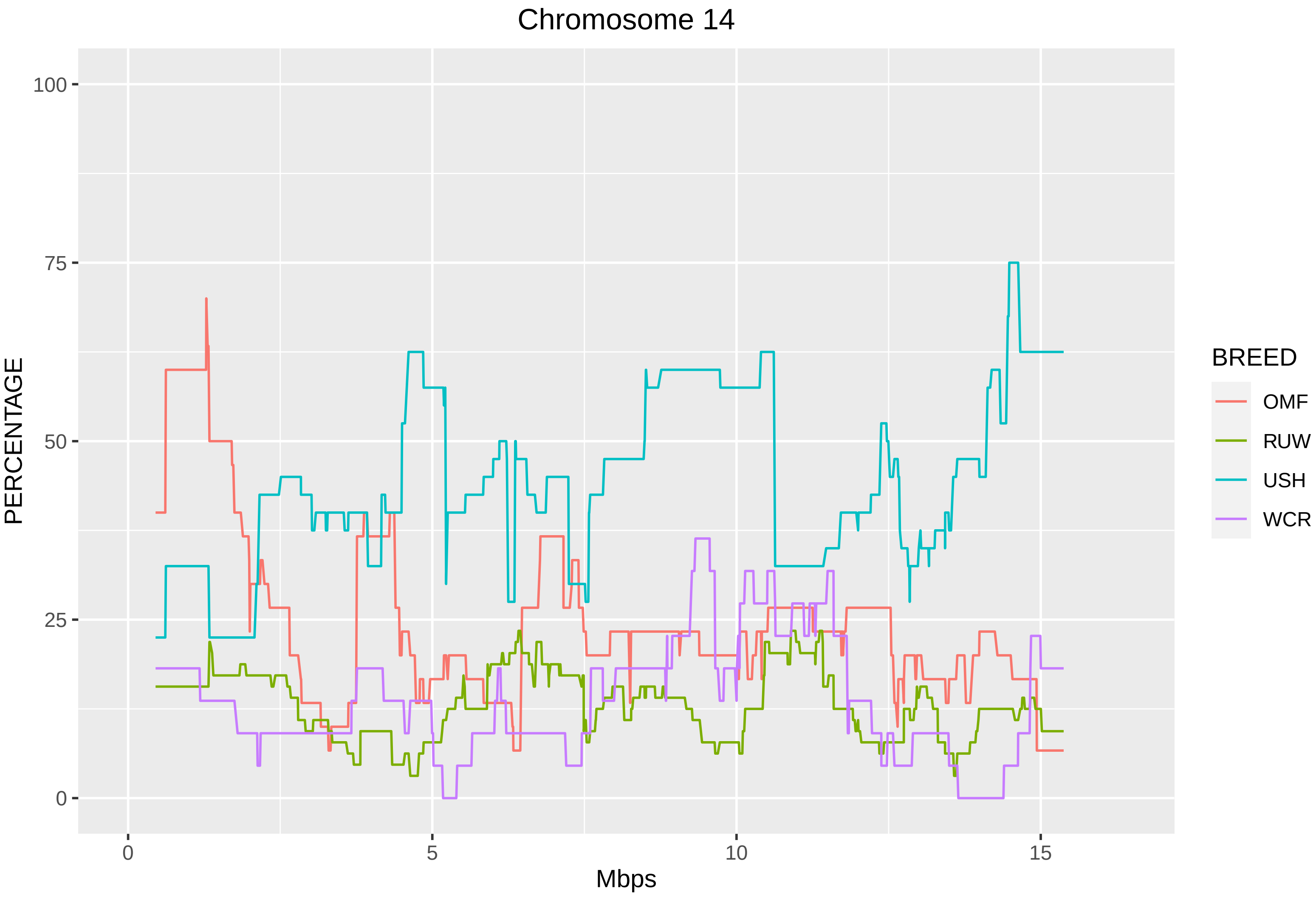
Chromosome 14
100
75
50
25
0
PERCENTAGE
0
5
10
15
Mbps
BREED
OMF
RUW
USH
WCR

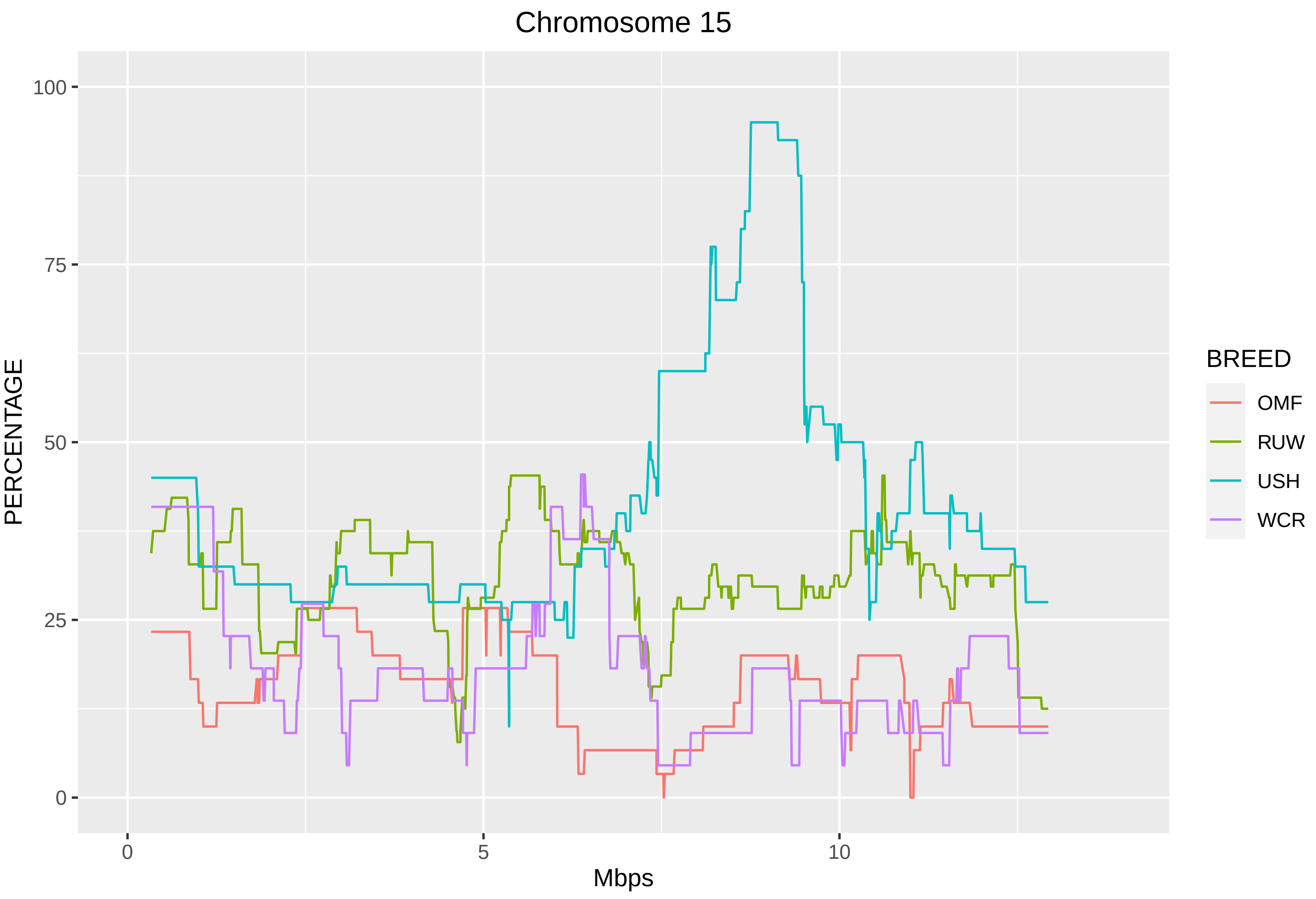

Chromosome 15
PERCENTAGE
100
75
50
25
0
0
5
10
Mbps
BREED
OMF
RUW
USH
WCR

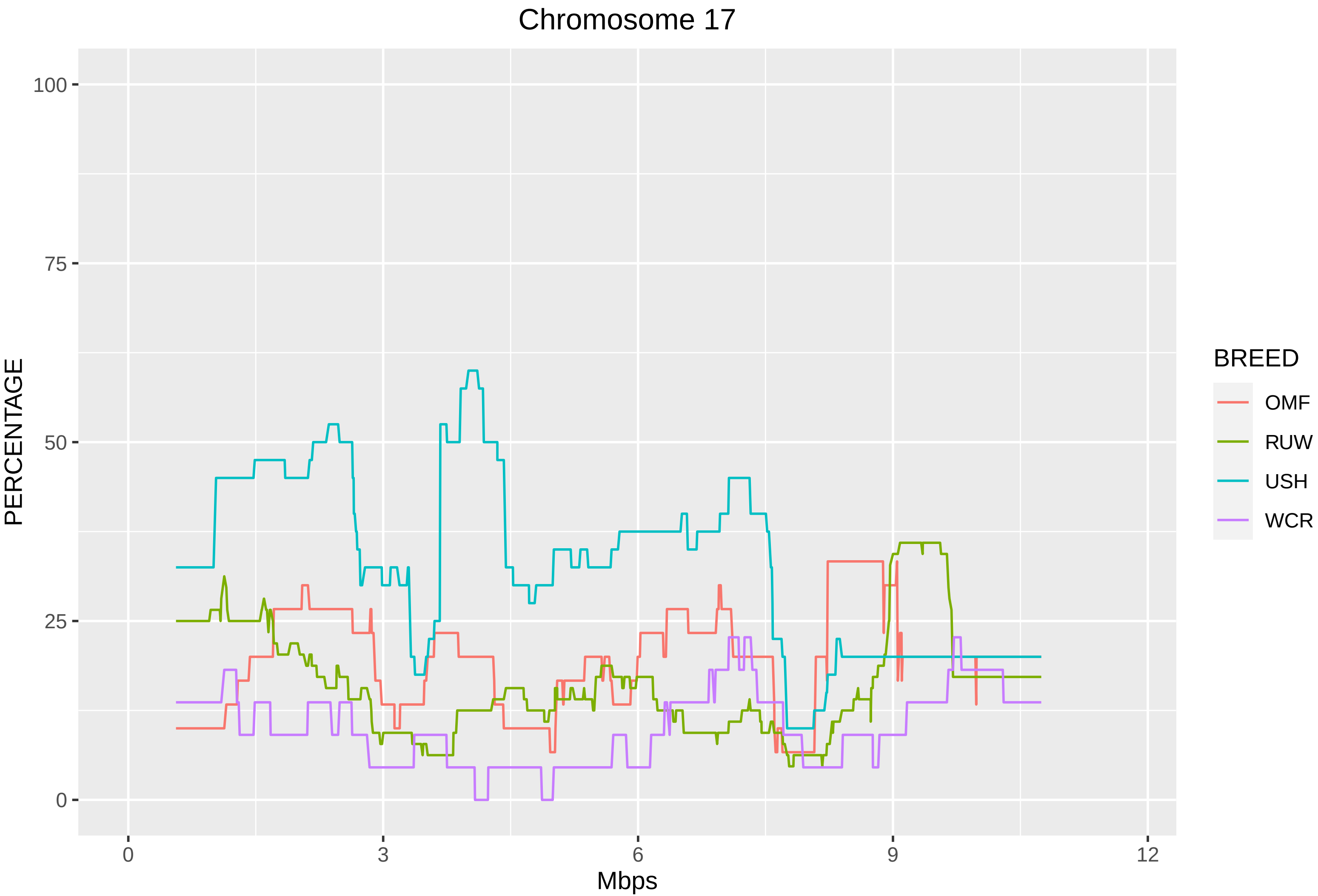
Chromosome 17
PERCENTAGE
100
75
50
25
0
0
3
6
9
12
Mbps
BREED
OMF
RUW
USH
WCR

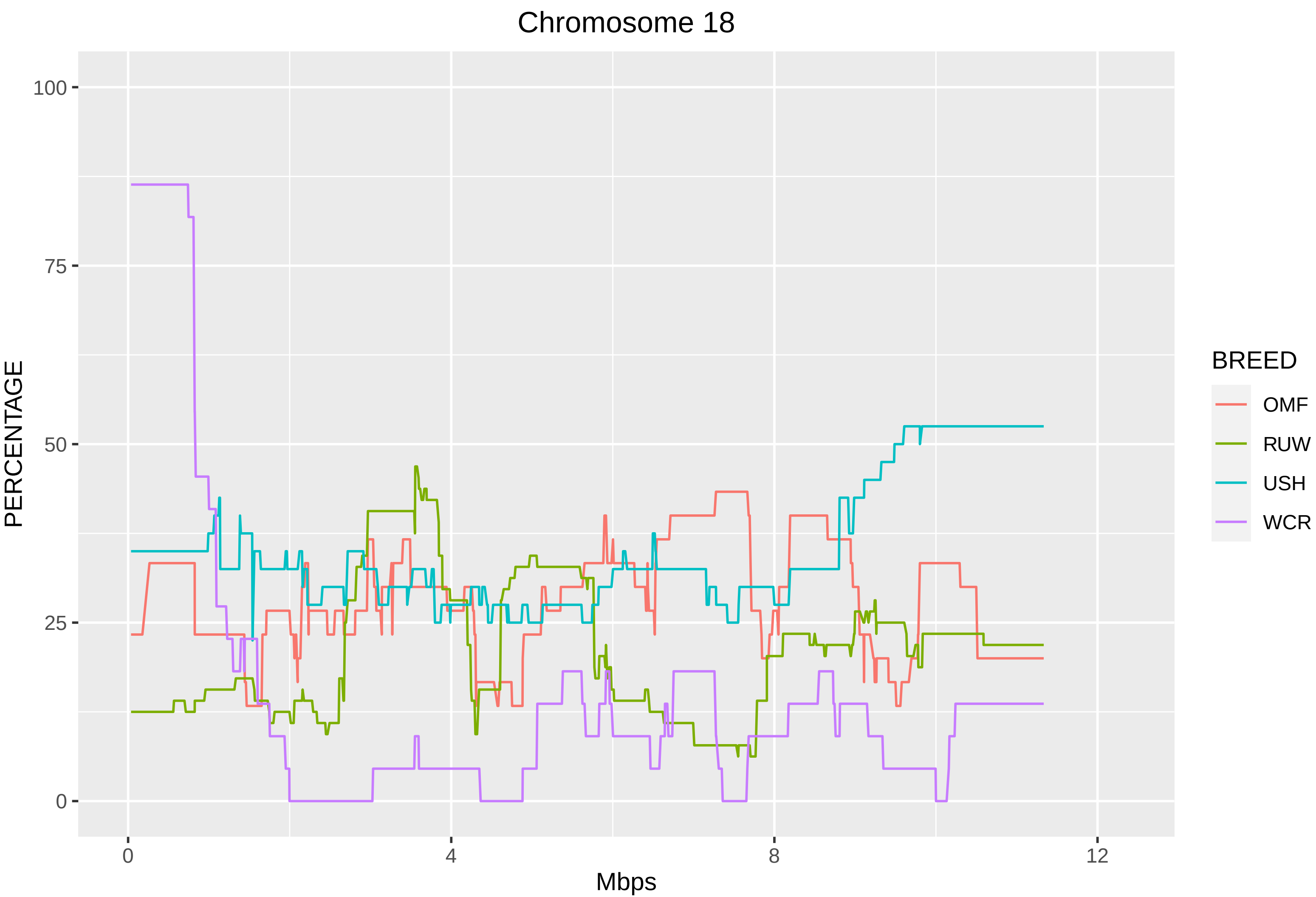
Chromosome 18
PERCENTAGE
100
75
50
25
0
0
4
8
12
Mbps
BREED
OMF
RUW
USH
WCR

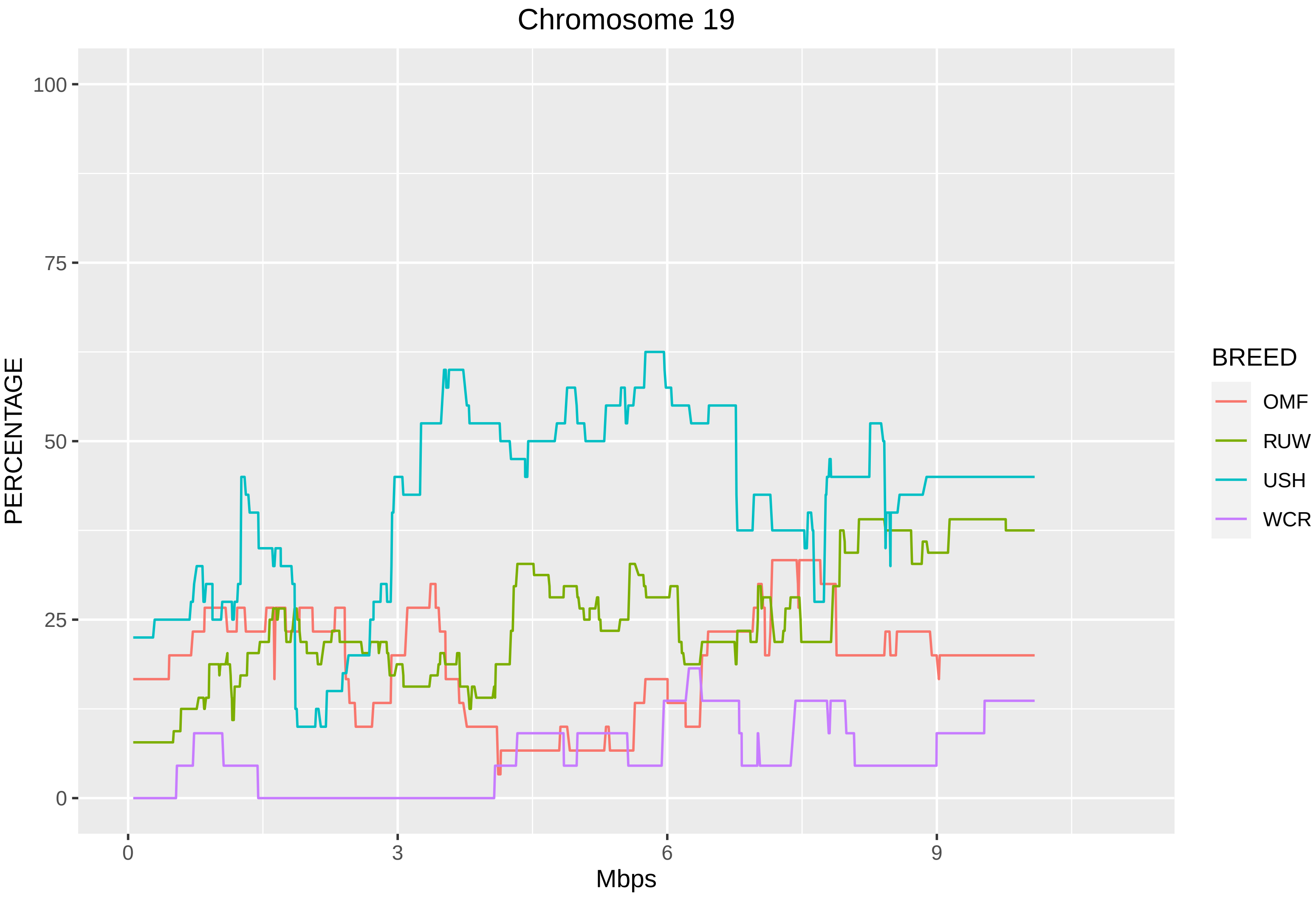
Chromosome 19
PERCENTAGE
100
75
50
25
0
0
3
6
9
Mbps
BREED
OMF
RUW
USH
WCR

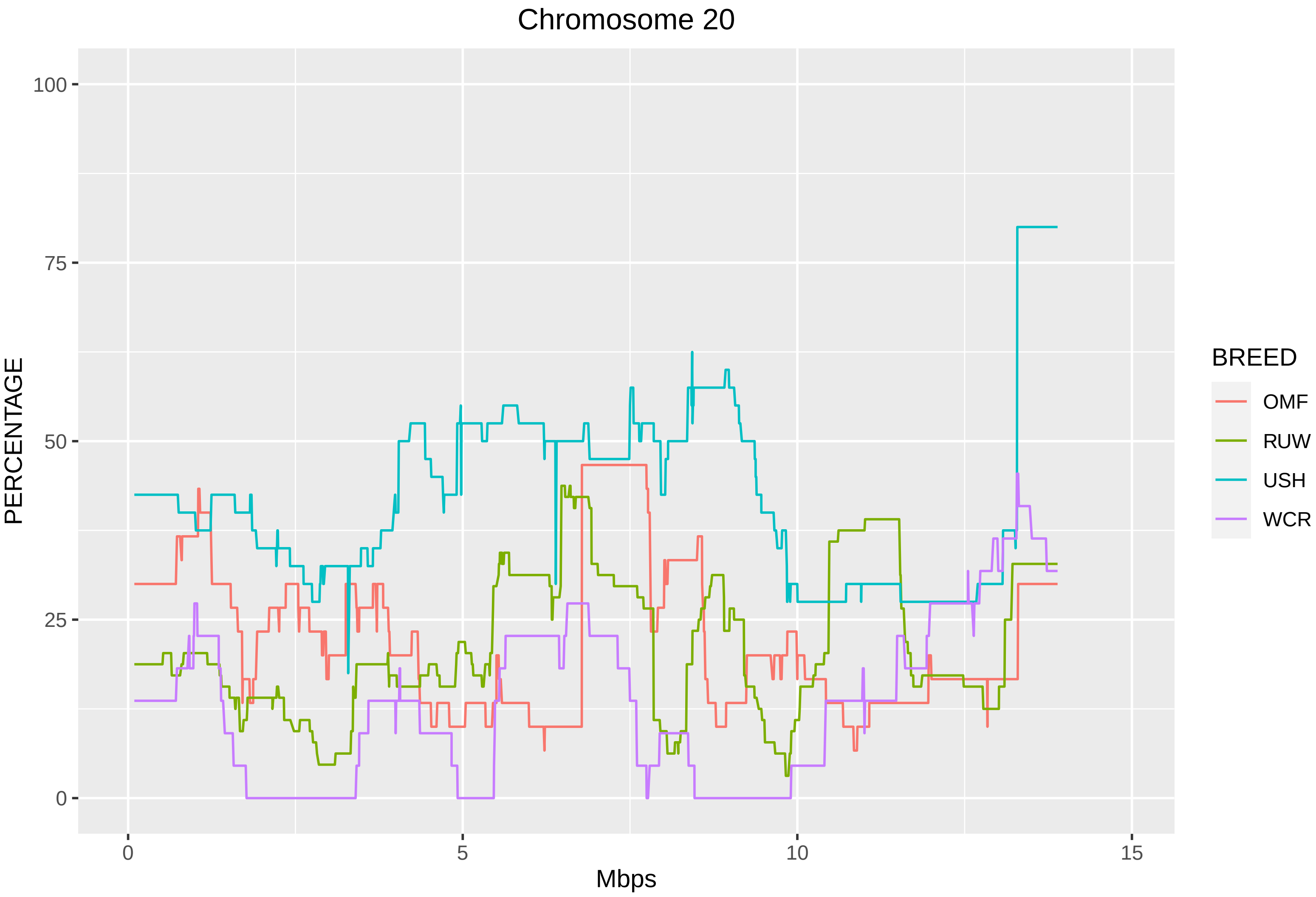

Chromosome 20
PERCENTAGE
100
75
50
25
0
0
5
10
15
Mbps
BREED
OMF
RUW
USH
WCR

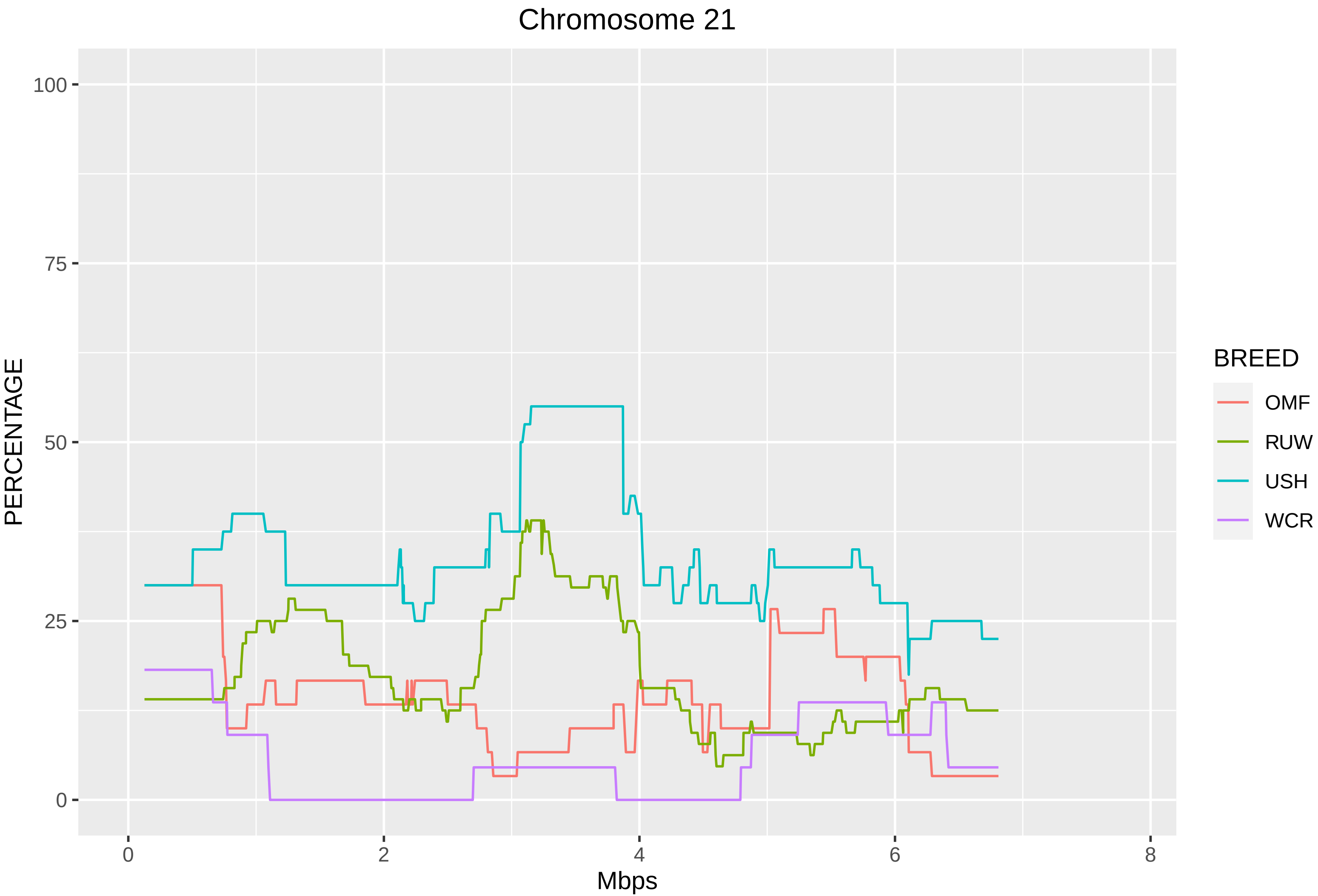

Chromosome 21
PERCENTAGE
100
75
50
25
0
0
2
4
6
8
Mbps
BREED
OMF
RUW
USH
WCR

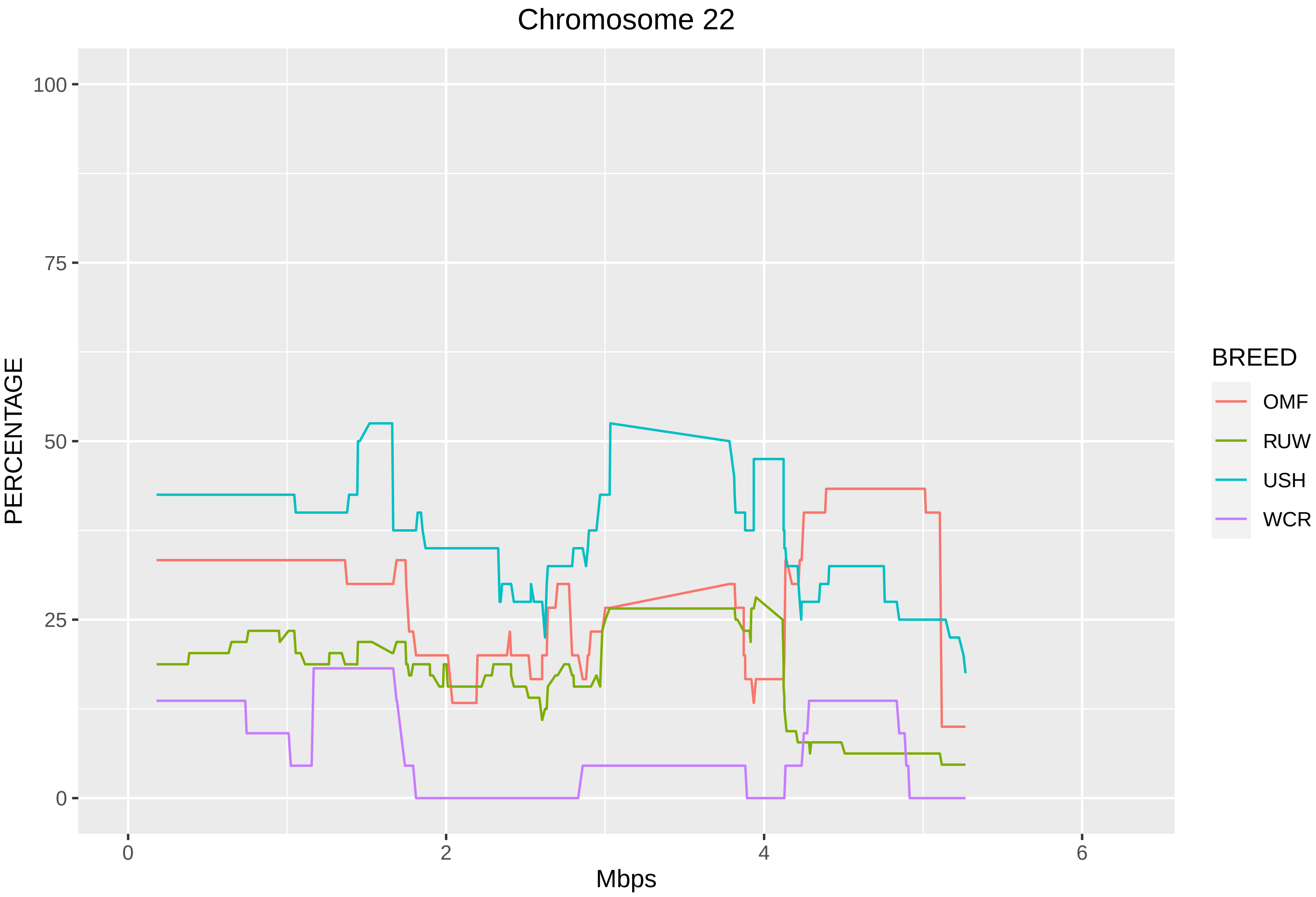
Chromosome 22
PERCENTAGE
100
75
50
25
0
0
2
4
6
Mbps
BREED
OMF
RUW
USH
WCR

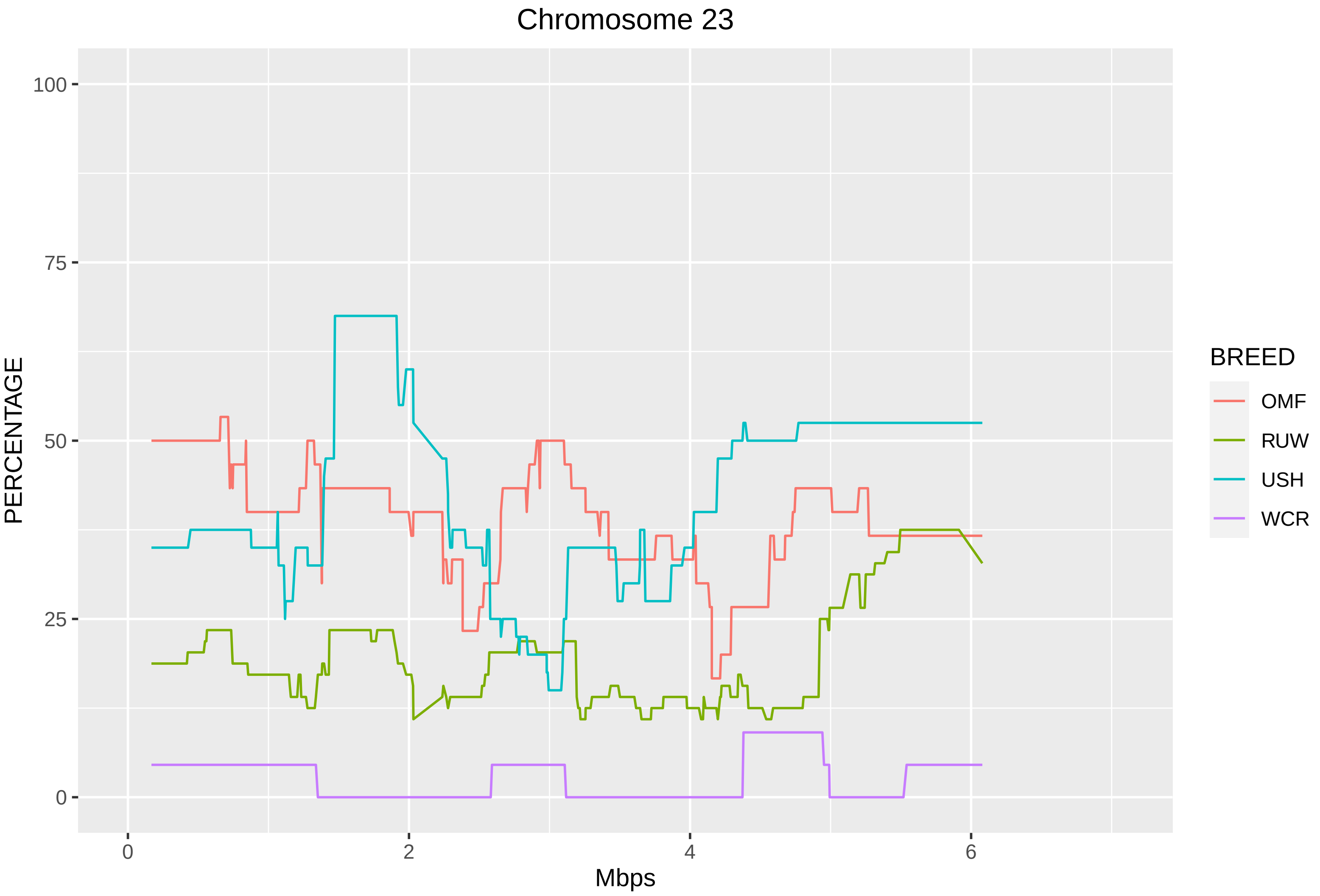
Chromosome 23
PERCENTAGE
100
75
50
25
0
0
2
4
6
Mbps
BREED
OMF
RUW
USH
WCR

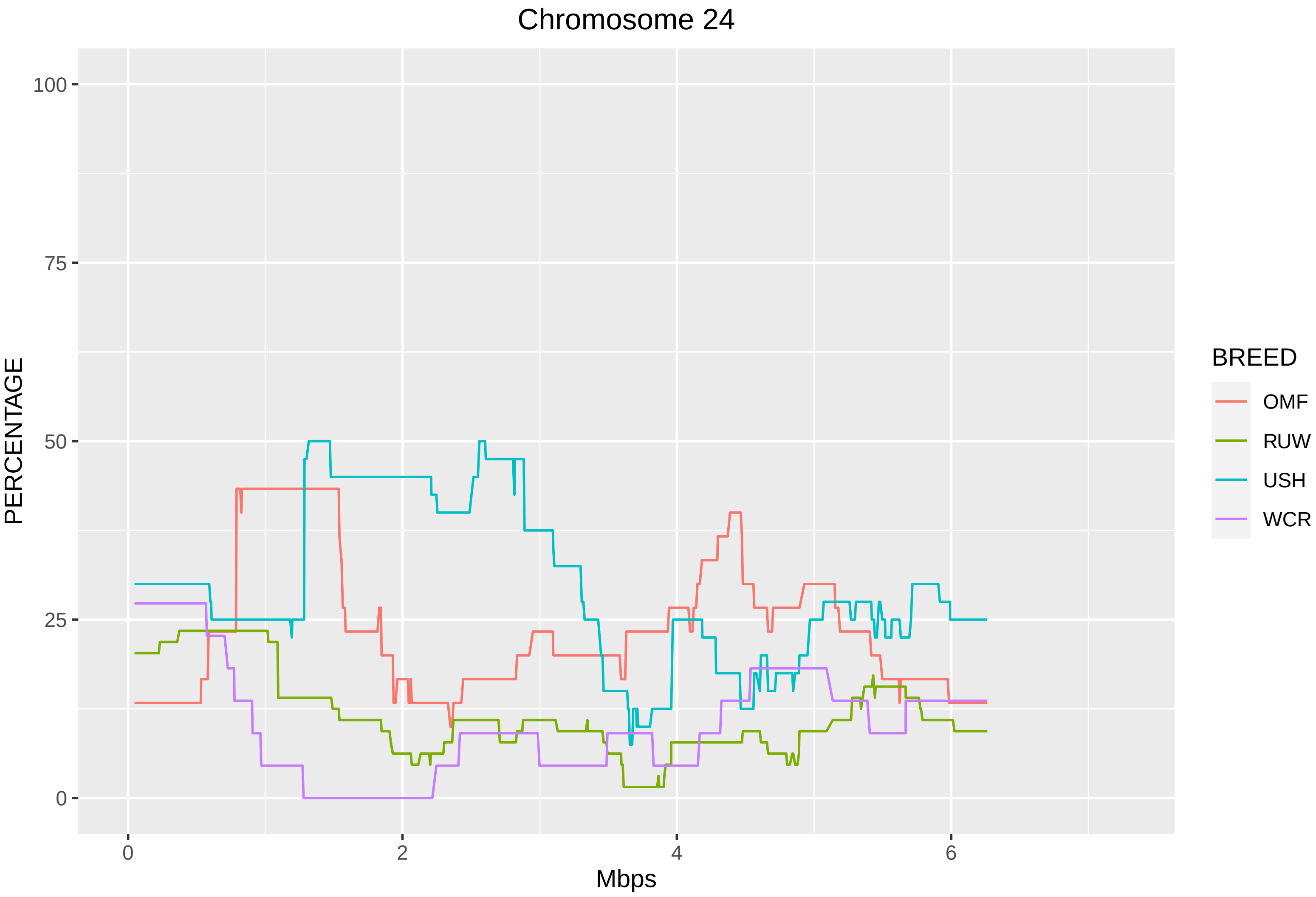

Chromosome 24
PERCENTAGE
100
75
50
25
0
0
2
4
6
Mbps
BREED
OMF
RUW
USH
WCR

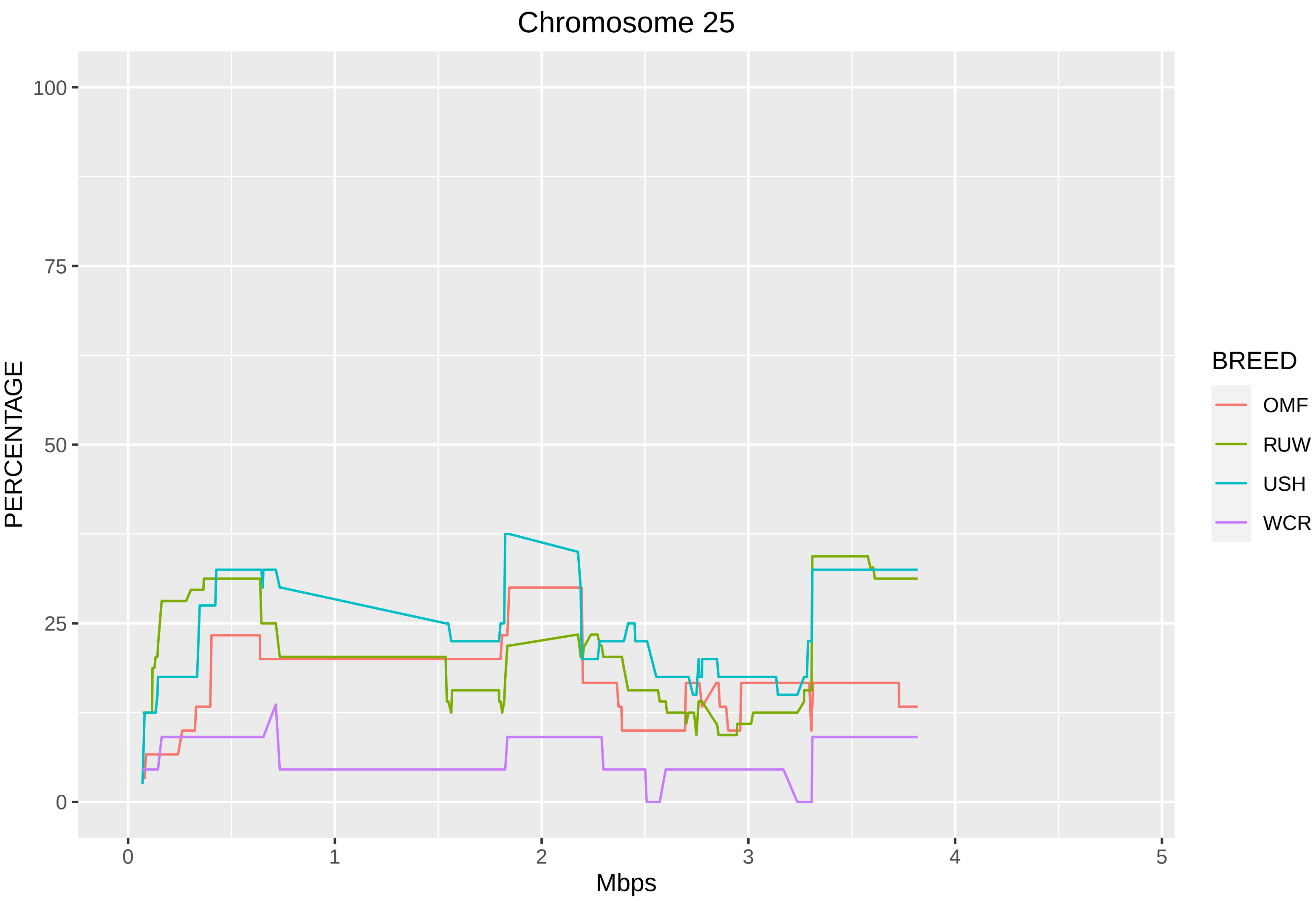

Chromosome 25
PERCENTAGE
100
75
50
25
0
0
1
2
3
4
5
Mbps
BREED
OMF
RUW
USH
WCR

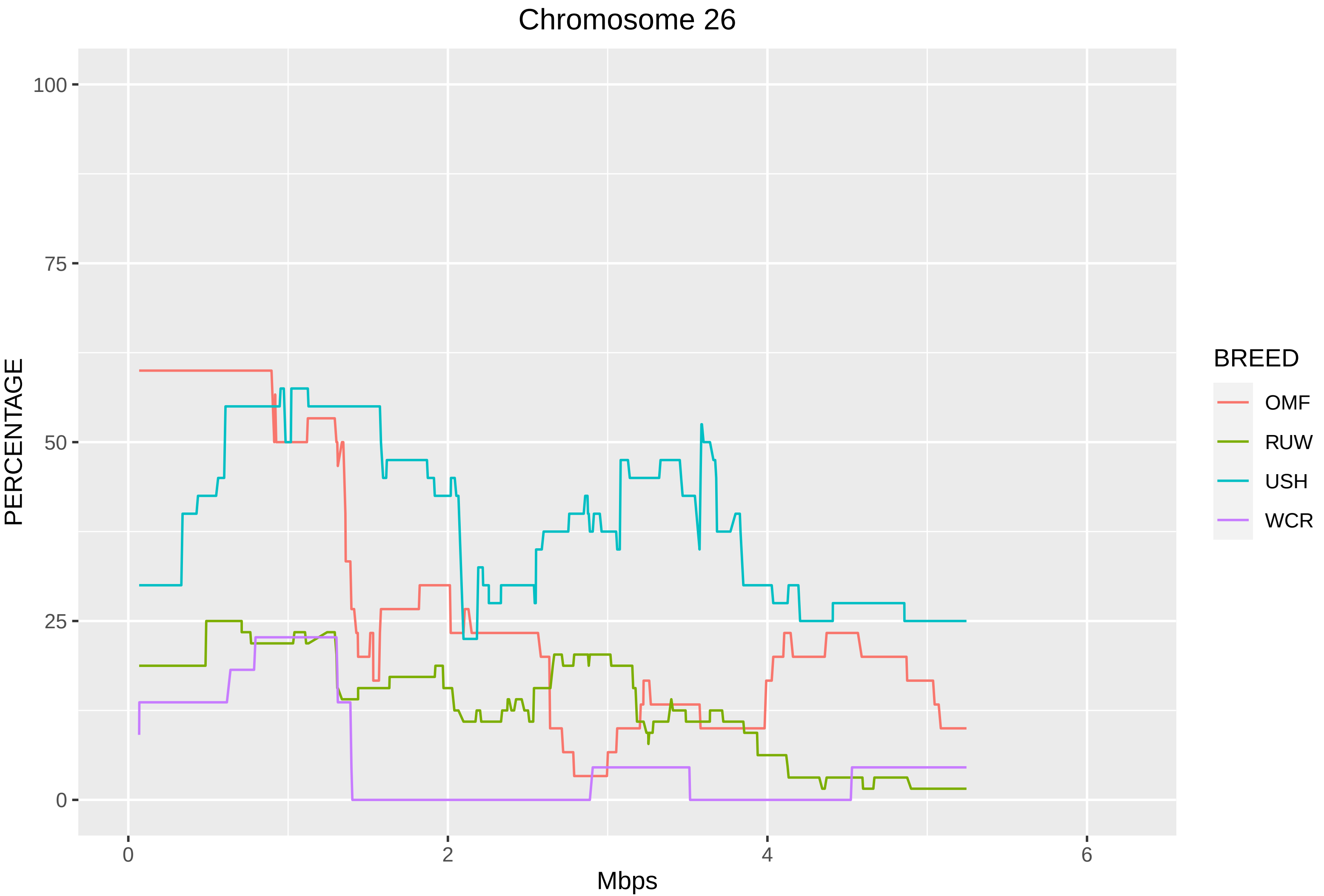
Chromosome 26
PERCENTAGE
100
75
50
25
0
0
2
4
6
Mbps
BREED
OMF
RUW
USH
WCR

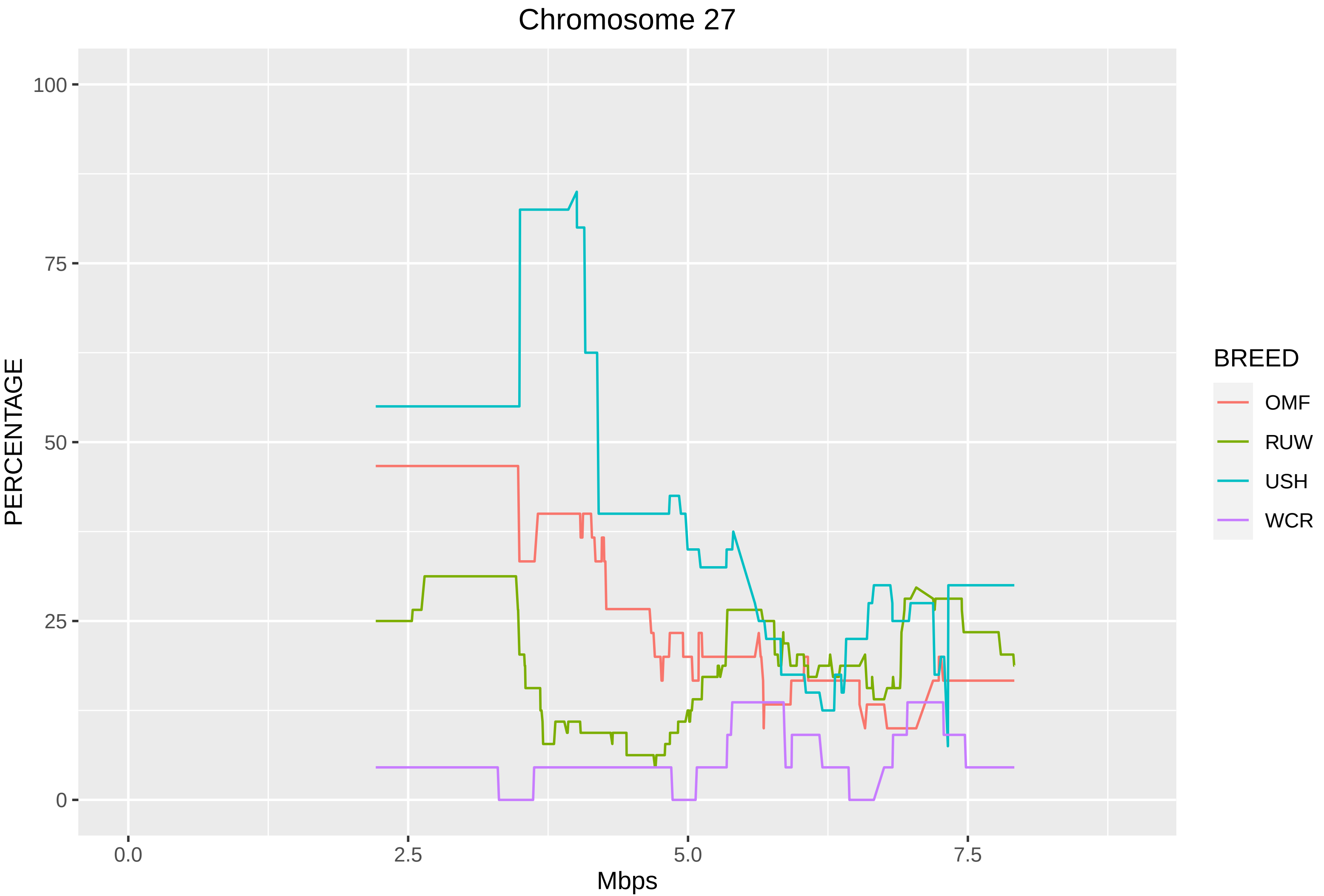
Chromosome 27
PERCENTAGE
100
75
50
25
0
0.0
2.5
5.0
7.5
Mbps
BREED
OMF
RUW
USH
WCR

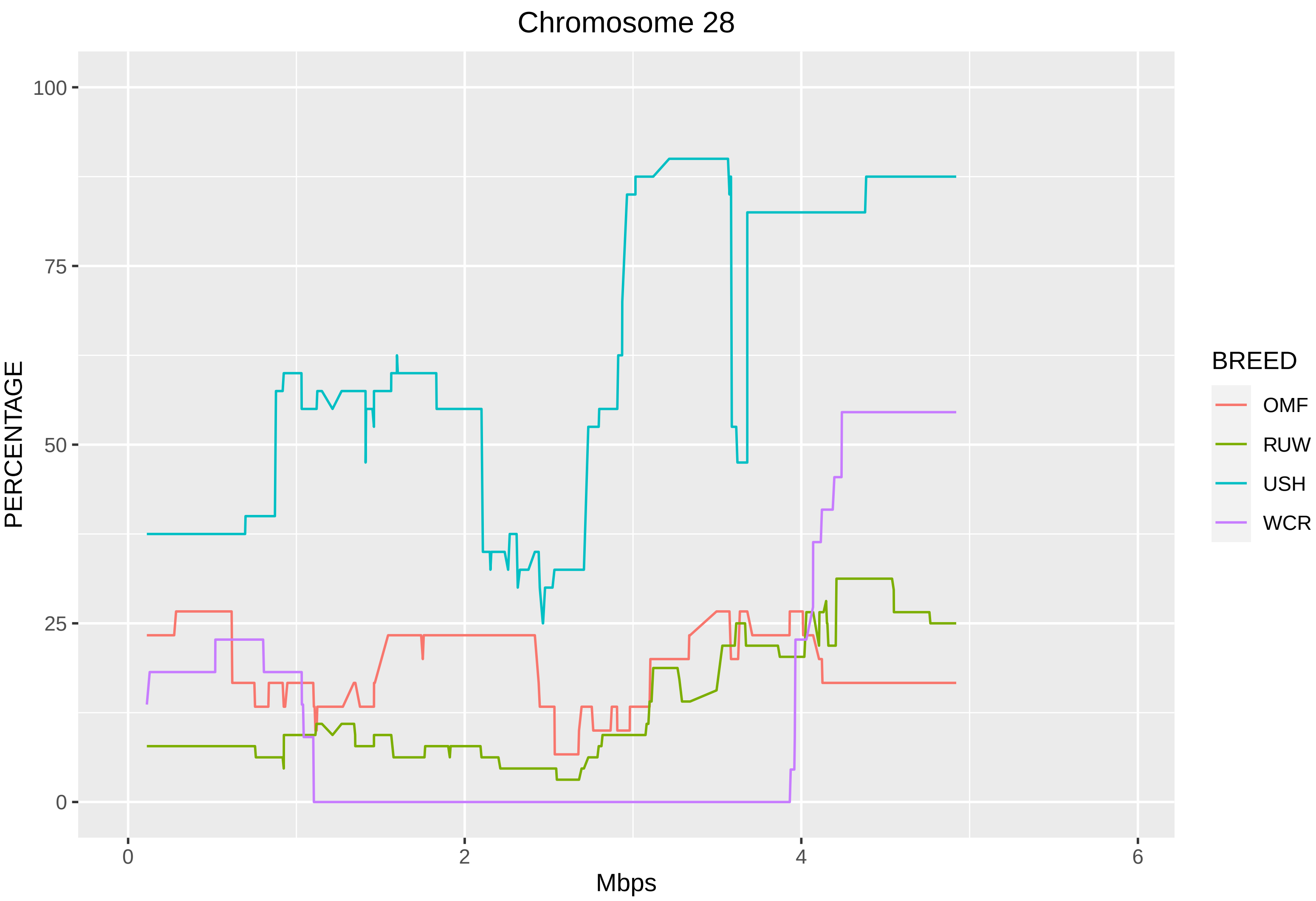
Chromosome 28
PERCENTAGE
100
75
50
25
0
0
2
4
6
Mbps
BREED
OMF
RUW
USH
WCR
