# Supplementary material for: Selective footprints and genes relevant to cold adaptation and other phenotypic traits are unscrambled in the genomes of divergently selected chicken breeds
Source: J Anim Sci Biotechnol. 2023 Feb 24;14:35. doi: 10.1186/s40104-022-00813-0 (PMC9951459; doi:10.1186/s40104-022-00813-0)
Supplement: Supplementary file 4 — Additional file 4: Fig. S4. Distribution of SNPs by FST value at pairwise comparison of breeds: A USH/OMF, B USH/RUW, and C USH/WCR. Breeds: OMF, Orloff Mille Fleur; RUW, Russian White; USH, Ushanka; WCR, White Cornish. [file 40104_2022_813_MOESM4_ESM.docx]

**Additional file 4: Fig. S4** Distribution of SNPs by *F*_ST_ value at pairwise comparison of breeds: **A** USH/OMF, **B** USH/RUW, and **C** USH/WCR. Breeds: OMF, Orloff Mille Fleur; RUW, Russian White; USH, Ushanka; WCR, White Cornish

**A**

**B**

**C**
