# Supplementary material for: Selective footprints and genes relevant to cold adaptation and other phenotypic traits are unscrambled in the genomes of divergently selected chicken breeds
Source: J Anim Sci Biotechnol. 2023 Feb 24;14:35. doi: 10.1186/s40104-022-00813-0 (PMC9951459; doi:10.1186/s40104-022-00813-0)
Supplement: Supplementary file 10 — Additional file 10: Table S7. Prioritized genes under selection distributed by chromosomes. Table S8. Prioritized genes under selection distributed by phenotypic category. Table S9. Prioritized genes under selection distributed by breed. [file 40104_2022_813_MOESM10_ESM.docx]

**Additional file 10: Table S7** Prioritized genes under selection distributed by chromosomes^1^

| **GGA** | ***n*** | **Gene symbols** |
| --- | --- | --- |
| 1 | 98 | *TMEM168*, *IFRD1*, *DOCK4*, *IMMP2L*, *IFNG*, *MYF6*, *MYF5*, *LRRIQ1*, *ALX1*, *KITLG*, *DUSP6*, *CRY1*, *TMEM263*, *NUAK1*, *SLC41A2*, *CHST11*, *HSP90B1*, *STAB2*, *IGF1*, *PMCH*, *AKR1B1*, *CALD1*, *STRAP*, *MGST1*, *PIK3C2G*, *PLCZ1*, *CAPZA3*, *SLCO1C1*, *SLCO1B3*, *SLCO1A2*, *IAPP*, *SOX5*, *TSPO*, *TTLL12*, *PNPLA3*, *CDKN1B*, *PTHLH*, *CCDC91*, *FGF23*, *NTF3*, *ANO2*, *FKBP4*, *SLC2A14*, *AICDA*, *OVST*, *MYH15*, *CYBB*, *NR0B1*, *TAB3*, *DMD*, *IL1RAPL1*, *POLA1*, *PCYT1B*, *PDK3*, *EIF2S3*, *APOO*, *SAT1*, *ACOT9*, *PRDX4*, *TSGA10*, *LYG2*, *AFF3*, *NMS*, *IL1R1*, *IL1RL1*, *IL18R1*, *FHL2*, *EDAR*, *PCDH9*, *PCDH17*, *PCDH8*, *TSC22D1*, *NUFIP1*, *GPALPP1*, *COG3*, *RNASEH2B*, *HSPH1*, *ATP8A2*, *CHORDC1*, *NOX4*, *TYR*, *GRM5*, *RAB38*, *MIR1657*, *FZD4*, *PRSS23*, *ME3*, *FAM181B*, *PRCP*, *DDIAS*, *RAB30*, *NARS2*, *THRSP*, *MYO7A*, *CAPN5*, *TSKU*, *WNT11*, *UVRAG* |
| 2 | 69 | *WNT3A*, *WNT9A*, *PTH2R*, *TNFRSF14*, *SETD2*, *HTR5A*, *MMR1L3*, *MRC1*, *ITGA8*, *ACBD7*, *CROT*, *ICA1*, *NXPH1*, *NDUFA4*, *EOMES*, *RBMS3*, *TGFBR2*, *GPD1L*, *CMTM7*, *EGFR*, *EXOC2*, *FOXF2*, *MYLK4*, *OVAL*, *OVALX*, *TNFRSF11A*, *MC4R*, *CDH12*, *ANKH*, *CTNND2*, *DAP*, *ROPN1L*, *CMBL*, *CCT5*, *SEMA5A*, *MTRR*, *FASTKD3*, *SRD5A1*, *GALNT1*, *INO80C*, *THOC1*, *GREB1L*, *ESCO1*, *ABHD3*, *MIB1*, *GATA6*, *RBBP8*, *CABLES1*, *TTR*, *TRAPPC8*, *MEP1B*, *DTNA*, *PRKDC*, *ST18*, *RB1CC1*, *HNF4G*, *WWP1*, *MMP16*, *RIPK2*, *OSGIN2*, *DECR1*, *CALB1*, *NECAB1*, *RUNX1T1*, *RIMS2*, *ZFPM2*, *HSF1*, *TRHR*, *CSMD3* |
| 3 | 25 | *FBXO11*, *EHBP1*, *MKKS*, *GLP1R*, *BTBD9*, *GGPS1*, *COA6*, *SLC35F3*, *MLK4*, *DISC1*, *TSNAX*, *EGLN1*, *GSTA3*, *LOC100859645* (*GSTAL2*), *GSTA2*, *TPO*, *PXDN*, *EIF2B4*, *HADHA*, *HADHB*, *POMC*, *NCOA1*, *CLIC5*, *TAS2R7*, *OPN5* |
| 4 | 69 | *IL2RG*, *ENSGALG00000046723*, *TLR2A*, *TLR2B*, *SFRP2*, *FGB*, *FGA*, *SPOCK3*, *ANXA10*, *DDX60*, *HPGDS*, *LEF1*, *HADH*, *CYP2U1*, *DKK2*, *LRP2BP*, *ACSL1*, *IRF2*, *GALNTL6*, *HMGB2*, *SAP30*, *HPGD*, *SPATA4*, *VEGFC*, *AREG*, *ANTXR2*, *FGF5*, *PKD2*, *SPP1*, *MEPE*, *DMP1*, *MAPK10*, *PITX2*, *ELOVL6*, *EGF*, *PLA2G12A*, *CASP6*, *PDLIM5*, *BMPR1B*, *EIF4E*, *MTTP*, *PPP3CA*, *BANK1*, *NFKB1*, *ENSGALG00000051269*, *FAM114A1*, *TLR1B*, *TLR1A*, *KLF3*, *TBC1D1*, *PGM2*, *PCDH7*, *CCKAR*, *RBPJ*, *PPARGC1A*, *ADGRA3*, *KCNIP4*, *PACRGL*, *SLIT2*, *NCAPG*, *LCORL*, *FAM184B*, *LAP3*, *QDPR*, *LDB2*, *TAPT1*, *FBXL5*, *NKX3-2*, *EVC2* |
| 5 | 50 | *PRMT3*, *NELL1*, *ANO5*, *SLC17A6*, *GAS2*, *SLC5A12*, *FIBIN*, *LGR4*, *BDNF*, *CCND1*, *FGF4*, *FGF3*, *SHANK2*, *NAT10*, *CAT*, *EHF*, *TRAF6*, *API5*, *SMOC1*, *SRSF5*, *ACTN1*, *THBS1*, *AVEN*, *RYR3*, *FMN1*, *GREM1*, *MEIS2*, *ACTC1*, *GJD2*, *SSTR1*, *ACYP1*, *FOS*, *JDP2*, *TGFB3*, *EML5*, *PSMC1*, *CALM1*, *FBLN5*, *NDUFB1*, *UNC79*, *VRK1*, *SIVA1*, *AKT1*, *JAG2*, *GCH1*, *CDKN3*, *SAV1*, *GNG2*, *PTGER2*, *BMP4* |
| 7 | 40 | *HIBCH*, *MSTN*, *FRZB*, *ACADL*, *MYL1*, *FN1*, *MLPH*, *AHR2*, *COL18A1*, *COL6A1*, *COL6A2*, *LOC124418108* (*BlSK1*), *LSS*, *PCNT*, *ITGB2*, *STAT1*, *STAT4*, *MYO1B*, *NABP1*, *ANKRD44*, *SF3B1*, *MIR1603*, *HSPD1*, *CREB1*, *KLF7*, *ADAM23*, *GPR1*, *NBEAL1*, *IDH1*, *PIKFYVE*, *PPP1R1C*, *SLC25A12*, *HAT1*, *LRP2*, *CERS6*, *MYLK*, *CCDC14*, *ITGB5*, *PARP9*, *MARCO* |
| 8 | 10 | *AMY2A*, *NTNG1*, *RFWD2*, *CACYBP*, *RABGAP1L*, *RNPC3*, *S1PR1*, *TGFBR3*, *LEPROT*, *LEPR* |
| 9 | 11 | *FOXL2*, *AHSG*, *MIR15B*, *IL12A*, *RARRES1*, *LXN*, *PTX3*, *MIR1658*, *DHX36*, *IGSF10*, *PFN2* |
| 10 | 12 | *TSPAN3*, *FBXO22*, *IREB2*, *CRABP1*, *DNAJA4*, *ACSBG1*, *IDH3A*, *ANXA2*, *TRPM1*, *TJP1*, *THSD4*, *CYP19A1* |
| 11 | 23 | *NKD1*, *ZNF423*, *PHKB*, *DNAJA2*, *GPT2*, *URI1*, *ZNF536*, *ZDHHC7*, *IRF8*, *SLC7A5*, *BANP*, *CIDEC*, *ZC3H18*, *IL17C*, *CYBA*, *RPL13*, *MC1R*, *CDH1*, *SNTB2*, *NFAT5*, *NQO1*, *ZFHX3*, *TAT* |
| 12 | 17 | *CACNA1D*, *ARF4*, *FLNB*, *DNASE1L3*, *QARS*, *ARIH2*, *PDHB*, *ACOX2*, *THOC7*, *MITF*, *CAV3*, *IRAK2*, *GHRL*, *RPL32*, *MBD4*, *RHO*, *TRH* |
| 13 | 5 | *TRPC7*, *LOC101749540*, *SMAD5*, *SH3RF2*, *STK32A* |
| 14 | 4 | *ACTB*, *MED9*, *USP7*, *MAPK8IP3* |
| 15 | 10 | *XBP1*, *LOC416924* (*THADA*), *IGLL1*, *CHCHD10*, *MMP11*, *MIF*, *GSTT1L*, *GSTT1*, *GGT5*, *SLC5A1* |
| 18 | 20 | *MYH1D*, *MYH1F*, *MYH1A*, *MYH1B*, *SOCS3*, *AFMID*, *TK1*, *TMC6*, *GCGR*, *NPB*, *SIRT7*, *PYCR1*, *SPAG9*, *WFIKKN2*, *CACNA1G*, *ACSF2*, *CD300LG*, *FADS6*, *KCTD2*, *MIF4GD* |
| 19 | 17 | *P2RX1*, *IFT22*, *CAMKK1*, *C1QBP*, *SLC43A2*, *SERPINF2*, *SERPINF1*, *HIC1*, *MIR1666*, *MIR1696*, *FOXN1*, *ALDOC*, *MIR451*, *GIT1*, *SLC6A4*, *BLMH*, *TRPV2* |
| 20 | 6 | *WISP2*, *ADA*, *HNF4A*, *FITM2*, *EYA2*, *SNAI1* |
| 21 | 1 | *KIF1B* |
| 23 | 4 | *CITED4*, *GBP1*, *THEMIS2*, *NR0B2* |
| 26 | 12 | *TULP1*, *FKBP5*, *CLPS*, *MAPK14*, *PIM1*, *KIF21B*, *ELF3*, *ARL8A*, *PTPN7*, *PHLDA3*, *TNNI1*, *LAD1* |
| 27 | 9 | *DAD1*, *MIR6644-2*, *GOSR2*, *WNT3*, *MAP3K14*, *GH*, *CD79B*, *LOC771308* (*GHRHR*), *FTSJ3* |
| 28 | 28 | *BTN1A1*, *PIAS4*, *EEF2*, *MYO1F*, *MIR6615*, *ZAP70*, *GDF3*, *COMP*, *CRTC1*, *CRLF1*, *UBA52*, *ELL*, *GDF15*, *JUND*, *IFI30*, *INSR*, *USE1*, *MYO9B*, *TMEM38A*, *MIR6693*, *SMIM7*, *CHERP*, *CALR3*, *KLF2*, *AP1M1*, *RAB8A*, *PTPRS*, *MIR6666* |

^1^GGA, chicken (*Gallus gallus*) chromosome; *n*, number of genes per chromosome

**Table S8** Prioritized genes under selection distributed by phenotypic category

| **Phenotypic category** | ***n*^1^** | **Gene symbols** |
| --- | --- | --- |
| cold tolerance | 84 | *MYH15*, *TSC22D1*, *NUFIP1*, *GPALPP1*, *COG3*, *RNASEH2B*, *TYR*, *PRCP*, *DDIAS*, *RAB30*, *NARS2*, *HTR5A*, *GPD1L*, *GREB1L*, *ESCO1*, *ABHD3*, *MIB1*, *GATA6*, *OSGIN2*, *DECR1*, *NECAB1*, *RUNX1T1*, *TRHR*, *COA6*, *DISC1*, *EVC2*, *PRMT3*, *ANO5*, *SLC17A6*, *GAS2*, *SLC5A12*, *FIBIN*, *EML5*, *UNC79*, *HSPD1*, *NBEAL1*, *IDH1*, *PIKFYVE*, *PPP1R1C*, *CERS6*, *ZNF536*, *TRH*, *LOC416924 (THADA)*, *AFMID*, *TK1*, *TMC6*, *GCGR*, *NPB*, *SIRT7*, *PYCR1*, *CD300LG*, *KCTD2*, *MIF4GD*, *TRPV2*, *KIF1B*, *PIK3C2G*, *NOX4*, *PRSS23*, *FAM181B*, *NDUFA4*, *RBBP8*, *CABLES1*, *HNF4G*, *WWP1*, *RIPK2*, *GGPS1*, *TPO*, *NELL1*, *LGR4*, *CAT*, *SPAG9*, *WFIKKN2*, *CACNA1G*, *ACSF2*, *FADS6*, *CLPS*, *TNNI1*, *CRLF1*, *APOO*, *ME3*, *BDNF*, *AKT1*, *LRP2*, *SOCS3* |
| domestication | 6 | *CTNND2*, *RHO*, *ALX1*, *GJD2*, *FKBP5*, *KITLG* |
| egg traits | 68 | *PIK3C2G*, *SLCO1C1*, *SLCO1B3*, *SLCO1A2*, *IAPP*, *TSPO*, *FGF23*, *CYBB*, *POLA1*, *PDK3*, *APOO*, *IL1RL1*, *MMR1L3*, *ICA1*, *RBMS3*, *OVAL*, *ANKH*, *DAP*, *ROPN1L*, *CMBL*, *CCT5*, *FASTKD3*, *RIMS2*, *CSMD3*, *EHBP1*, *ENSGALG00000046723*, *SPP1*, *MEPE*, *EIF4E*, *PPP3CA*, *BANK1*, *ENSGALG00000051269*, *NAT10*, *CALM1*, *LRP2*, *RARRES1*, *ACSF2*, *P2RX1*, *GDF3*, *COMP*, *CRTC1*, *CRLF1*, *ELL*, *GDF15*, *IFI30*, *KITLG*, *PLCZ1*, *PTHLH*, *OVST*, *ITGA8*, *OVALX*, *NCOA1*, *BMPR1B*, *NCAPG*, *ACTC1*, *ACADL*, *LEPR*, *FOXL2*, *PTX3*, *ACSBG1*, *ANXA2*, *GH*, *LOC771308* (*GHRHR*), *JUND*, *INSR*, *CALB1*, *FN1*, *ITGB5* |
| energy and feed intake | 27 | *SAT1*, *NMS*, *FOXF2*, *TSNAX*, *ANTXR2*, *SHANK2*, *EHF*, *COL18A1*, *TGFBR3*, *NR0B2*, *IAPP*, *FGF23*, *ACOT9*, *TTR*, *PGM2*, *NAT10*, *COL6A2*, *LOC416924* (*THADA*), *HSP90B1*, *CHORDC1*, *NOX4*, *TBC1D1*, *CCKAR*, *TGFB3*, *MLPH*, *LEPR*, *IDH3A* |
| fat metabolism | 83 | *FKBP4*, *ACOT9*, *MYO7A*, *ACBD7*, *CROT*, *TNFRSF11A*, *ZFPM2*, *GGPS1*, *HADHA*, *HADHB*, *SPOCK3*, *ANXA10*, *LRP2BP*, *ELOVL6*, *PLA2G12A*, *KLF3*, *PGM2*, *JDP2*, *JAG2*, *AHR2*, *LSS*, *KLF7*, *GPR1*, *HAT1*, *CCDC14*, *RABGAP1L*, *ZNF423*, *GPT2*, *URI1*, *CIDEC*, *PDHB*, *CHCHD10*, *SPAG9*, *FADS6*, *BLMH*, *FITM2*, *TULP1*, *CLPS*, *KIF21B*, *USE1*, *MYO9B*, *TMEM38A*, *MIR6693*, *SMIM7*, *CHERP*, *CALR3*, *AP1M1*, *RAB8A*, *PTPRS*, *MIR6666*, *IGF1*, *AKR1B1*, *SOX5*, *APOO*, *ME3*, *FBXO11*, *EHBP1*, *MKKS*, *EGLN1*, *MTTP*, *TBC1D1*, *PPARGC1A*, *HIBCH*, *COL6A1*, *ITGB2*, *MYLK*, *SLC7A5*, *FLNB*, *ACOX2*, *SOCS3*, *MAPK14*, *ZAP70*, *ELL*, *KLF2*, *PNPLA3*, *THRSP*, *POMC*, *ACSL1*, *FBLN5*, *ACADL*, *ACSBG1*, *GHRL*, *HNF4A* |
| growth, meat, carcass | 131 | *MYF6*, *MYF5*, *TMEM263*, *CHST11*, *IGF1*, *PMCH*, *MGST1*, *PNPLA3*, *CCDC91*, *DMD*, *PCYT1B*, *FHL2*, *CHORDC1*, *MIR1657*, *PRSS23*, *ME3*, *FAM181B*, *THRSP*, *WNT9A*, *PTH2R*, *SETD2*, *ITGA8*, *MC4R*, *CDH12*, *RBBP8*, *TRAPPC8*, *MEP1B*, *DTNA*, *PRKDC*, *ST18*, *RB1CC1*, *HNF4G*, *WWP1*, *MKKS*, *SLC35F3*, *TPO*, *POMC*, *FGB*, *HPGDS*, *HADH*, *GALNTL6*, *SAP30*, *AREG*, *FGF5*, *DMP1*, *PITX2*, *PDLIM5*, *TBC1D1*, *PCDH7*, *CCKAR*, *RBPJ*, *PPARGC1A*, *ADGRA3*, *KCNIP4*, *PACRGL*, *SLIT2*, *NCAPG*, *LCORL*, *FAM184B*, *LAP3*, *QDPR*, *LDB2*, *TAPT1*, *FBXL5*, *NKX3-2*, *NELL1*, *CCND1*, *FGF4*, *ACTN1*, *FMN1*, *GREM1*, *MEIS2*, *ACTC1*, *PSMC1*, *FBLN5*, *CDKN3*, *HIBCH*, *MSTN*, *MYL1*, *MLPH*, *COL6A1*, *COL6A2*, *NABP1*, *SLC25A12*, *LEPROT*, *LEPR*, *MIR1658*, *THSD4*, *NKD1*, *PHKB*, *SLC7A5*, *ZC3H18*, *TAT*, *FLNB*, *CAV3*, *GHRL*, *SH3RF2*, *MMP11*, *GSTT1L*, *MYH1D*, *MYH1F*, *MYH1A*, *MYH1B*, *MIR1666*, *GIT1*, *SLC6A4*, *EYA2*, *MAPK14*, *PTPN7*, *TNNI1*, *GH*, *LOC771308* (*GHRHR*), *FTSJ3*, *INSR*, *KLF2*, *IFRD1*, *PRDX4*, *TYR*, *TGFBR2*, *TLR2A*, *EGF*, *EVC2*, *BDNF*, *TGFB3*, *ITGB5*, *AHSG*, *MC1R*, *DNASE1L3*, *ACTB*, *EEF2*, *ACOX2* |
| immunity | 140 | *TMEM168*, *IFRD1*, *DOCK4*, *IMMP2L*, *IFNG*, *DUSP6*, *HSP90B1*, *AKR1B1*, *TTLL12*, *CDKN1B*, *AICDA*, *TAB3*, *IL1RAPL1*, *EIF2S3*, *PRDX4*, *LYG2*, *IL1R1*, *IL18R1*, *ATP8A2*, *TNFRSF14*, *MRC1*, *NDUFA4*, *EOMES*, *TGFBR2*, *CMTM7*, *OVALX*, *SEMA5A*, *INO80C*, *THOC1*, *RIPK2*, *CALB1*, *MLK4*, *GSTA3*, *EIF2B4*, *IL2RG*, *TLR2A*, *FGA*, *DDX60*, *LEF1*, *CYP2U1*, *ACSL1*, *IRF2*, *HPGD*, *VEGFC*, *MAPK10*, *EGF*, *CASP6*, *NFKB1*, *FAM114A1*, *TLR1B*, *TLR1A*, *BDNF*, *TRAF6*, *API5*, *SMOC1*, *SRSF5*, *THBS1*, *AVEN*, *TGFB3*, *NDUFB1*, *SIVA1*, *AKT1*, *GCH1*, *GNG2*, *FN1*, *PCNT*, *ITGB2*, *STAT1*, *SF3B1*, *MIR1603*, *ITGB5*, *PARP9*, *MARCO*, *RFWD2*, *CACYBP*, *RNPC3*, *S1PR1*, *AHSG*, *MIR15B*, *IL12A*, *LXN*, *PTX3*, *DHX36*, *PFN2*, *TSPAN3*, *DNAJA4*, *ACSBG1*, *ANXA2*, *DNAJA2*, *ZDHHC7*, *IRF8*, *BANP*, *IL17C*, *CYBA*, *MC1R*, *NFAT5*, *ZFHX3*, *ARF4*, *DNASE1L3*, *ARIH2*, *ACOX2*, *THOC7*, *IRAK2*, *RPL32*, *MBD4*, *ACTB*, *MED9*, *USP7*, *MAPK8IP3*, *XBP1*, *IGLL1*, *MIF*, *GGT5*, *SLC5A1*, *SOCS3*, *WFIKKN2*, *C1QBP*, *SLC43A2*, *SERPINF2*, *SERPINF1*, *HIC1*, *MIR451*, *WISP2*, *ADA*, *HNF4A*, *GBP1*, *THEMIS2*, *PIM1*, *GOSR2*, *MAP3K14*, *CD79B*, *BTN1A1*, *PIAS4*, *EEF2*, *UBA52*, *JUND*, *KLF3*, *CALM1*, *GREM1*, *MYLK* |
| reproduction | 54 | *LRRIQ1*, *PLCZ1*, *CAPZA3*, *PTHLH*, *OVST*, *CAPN5*, *NXPH1*, *SRD5A1*, *GALNT1*, *TTR*, *NCOA1*, *CLIC5*, *OPN5*, *SFRP2*, *HMGB2*, *SPATA4*, *BMPR1B*, *SSTR1*, *SAV1*, *ACADL*, *CREB1*, *FOXL2*, *TJP1*, *RPL13*, *CACNA1G*, *CITED4*, *ELF3*, *MYF5*, *SLCO1C1*, *PNPLA3*, *CDKN1B*, *NTF3*, *THRSP*, *POMC*, *HPGDS*, *AREG*, *CCKAR*, *KCNIP4*, *SLIT2*, *FAM184B*, *FBXL5*, *FBLN5*, *AKT1*, *FN1*, *GPR1*, *TAT*, *GHRL*, *RHO*, *HNF4A*, *CRTC1*, *DNAJA4*, *GSTT1L*, *LOC771308* (*GHRHR*), *HSP90B1* |
| response to heat | 35 | *NTF3*, *HSPH1*, *NOX4*, *HSF1*, *FBXO11*, *LOC100859645* (*GSTAL2*), *MTTP*, *CAT*, *ACYP1*, *FOS*, *VRK1*, *IDH3A*, *CDH1*, *SNTB2*, *NQO1*, *TRPC7*, *GSTT1*, *HSP90B1*, *CHORDC1*, *RB1CC1*, *CALB1*, *ACSL1*, *PITX2*, *CASP6*, *EHF*, *ACTN1*, *RYR3*, *DNAJA4*, *CYBA*, *XBP1*, *GSTT1L*, *SLC5A1*, *IFRD1*, *IL1R1*, *EEF2* |
| skin, feather, other skin appendages | 76 | *ALX1*, *KITLG*, *NUAK1*, *SLC41A2*, *STAB2*, *STRAP*, *SOX5*, *SLC2A14*, *TSGA10*, *AFF3*, *EDAR*, *PCDH9*, *PCDH17*, *PCDH8*, *GRM5*, *RAB38*, *FZD4*, *WNT11*, *UVRAG*, *WNT3A*, *EGFR*, *MYLK4*, *MTRR*, *CABLES1*, *MMP16*, *GLP1R*, *BTBD9*, *GSTA2*, *TLR2B*, *DKK2*, *LGR4*, *FGF3*, *RYR3*, *PTGER2*, *BMP4*, *FRZB*, *LOC124418108* (*BlSK1*), *ANKRD44*, *ADAM23*, *CRABP1*, *CYP19A1*, *CACNA1D*, *MITF*, *LOC101749540*, *SMAD5*, *STK32A*, *IFT22*, *CAMKK1*, *FOXN1*, *SNAI1*, *ARL8A*, *PHLDA3*, *LAD1*, *DAD1*, *WNT3*, *CHST11*, *FHL2*, *EOMES*, *PRKDC*, *LEF1*, *PCDH7*, *FGF4*, *SMOC1*, *GREM1*, *GNG2*, *MLPH*, *LRP2*, *IRF8*, *CDH1*, *TRPC7*, *TYR*, *TPO*, *HPGDS*, *EGF*, *ACTN1*, *MC1R* |
| stress and adaptation | 47 | *CRY1*, *CALD1*, *NR0B1*, *TSKU*, *EXOC2*, *EGLN1*, *PXDN*, *TAS2R7*, *GJD2*, *STAT4*, *MYO1B*, *MYLK*, *AMY2A*, *NTNG1*, *IGSF10*, *FBXO22*, *IREB2*, *QARS*, *MIR1696*, *ALDOC*, *FKBP5*, *MIR6644-2*, *MYO1F*, *MIR6615*, *ZAP70*, *MGST1*, *CYBB*, *IL1R1*, *TNFRSF11A*, *LOC100859645* (*GSTAL2*), *GSTA2*, *SFRP2*, *DDX60*, *FGF5*, *NFKB1*, *BMP4*, *STAT1*, *IDH3A*, *NFAT5*, *MAPK8IP3*, *CAMKK1*, *PRDX4*, *EOMES*, *BMPR1B*, *LGR4*, *CAT*, *EHF* |
| thermosensation | 5 | *ANO2*, *PKD2*, *TRPM1*, *TRPV2*, *TRPC7* |

^1^*n*, number of genes per phenotypic category

**Table S9** Prioritized genes under selection distributed by breed

| **Breed** | ***n*^1^** | **Gene symbols** |
| --- | --- | --- |
| USH | 480 | *TMEM168*, *IFRD1*, *DOCK4*, *IMMP2L*, *IFNG*, *AKR1B1*, *CALD1*, *STRAP*, *MGST1*, *PIK3C2G*, *PLCZ1*, *CAPZA3*, *SLCO1C1*, *SLCO1B3*, *SLCO1A2*, *IAPP*, *SOX5*, *TSPO*, *TTLL12*, *PNPLA3*, *CDKN1B*, *PTHLH*, *CCDC91*, *FGF23*, *NTF3*, *ANO2*, *FKBP4*, *SLC2A14*, *AICDA*, *OVST*, *MYH15*, *CYBB*, *NR0B1*, *TAB3*, *DMD*, *IL1RAPL1*, *POLA1*, *PCYT1B*, *PDK3*, *EIF2S3*, *APOO*, *SAT1*, *ACOT9*, *PRDX4*, *TSGA10*, *LYG2*, *AFF3*, *NMS*, *IL1R1*, *IL1RL1*, *IL18R1*, *FHL2*, *EDAR*, *PCDH9*, *PCDH17*, *PCDH8*, *TSC22D1*, *NUFIP1*, *GPALPP1*, *COG3*, *RNASEH2B*, *HSPH1*, *ATP8A2*, *CHORDC1*, *NOX4*, *TYR*, *GRM5*, *RAB38*, *MIR1657*, *FZD4*, *PRSS23*, *ME3*, *FAM181B*, *PRCP*, *DDIAS*, *RAB30*, *NARS2*, *THRSP*, *MYO7A*, *CAPN5*, *TSKU*, *WNT11*, *UVRAG*, *WNT3A*, *WNT9A*, *PTH2R*, *TNFRSF14*, *SETD2*, *HTR5A*, *ITGA8*, *ACBD7*, *CROT*, *EOMES*, *RBMS3*, *TGFBR2*, *GPD1L*, *CMTM7*, *EGFR*, *EXOC2*, *FOXF2*, *MYLK4*, *OVAL*, *OVALX*, *TNFRSF11A*, *MC4R*, *CDH12*, *ANKH*, *CTNND2*, *DAP*, *ROPN1L*, *CMBL*, *CCT5*, *SEMA5A*, *MTRR*, *FASTKD3*, *SRD5A1*, *GALNT1*, *INO80C*, *THOC1*, *GREB1L*, *ESCO1*, *ABHD3*, *MIB1*, *GATA6*, *RBBP8*, *CABLES1*, *TTR*, *TRAPPC8*, *MEP1B*, *DTNA*, *PRKDC*, *ST18*, *RB1CC1*, *DECR1*, *CALB1*, *NECAB1*, *RUNX1T1*, *RIMS2*, *ZFPM2*, *HSF1*, *TRHR*, *CSMD3*, *FBXO11*, *EHBP1*, *MKKS*, *GSTA3*, *LOC100859645* (*GSTAL2*) , *GSTA2*, *TPO*, *PXDN*, *HADHA*, *HADHB*, *POMC*, *NCOA1*, *CLIC5*, *TAS2R7*, *OPN5*, *IL2RG*, *TLR2A*, *TLR2B*, *SFRP2*, *HPGDS*, *LEF1*, *HADH*, *CYP2U1*, *DKK2*, *LRP2BP*, *ACSL1*, *IRF2*, *GALNTL6*, *HMGB2*, *SAP30*, *HPGD*, *SPATA4*, *VEGFC*, *AREG*, *ANTXR2*, *FGF5*, *PKD2*, *SPP1*, *MEPE*, *DMP1*, *MAPK10*, *PITX2*, *ELOVL6*, *EGF*, *PLA2G12A*, *CASP6*, *PDLIM5*, *BMPR1B*, *EIF4E*, *MTTP*, *PPP3CA*, *BANK1*, *NFKB1*, *ENSGALG00000051269*, *FAM114A1*, *TLR1B*, *TLR1A*, *KLF3*, *TBC1D1*, *PGM2*, *PCDH7*, *CCKAR*, *RBPJ*, *PPARGC1A*, *ADGRA3*, *KCNIP4*, *PACRGL*, *SLIT2*, *NCAPG*, *LCORL*, *FAM184B*, *LAP3*, *QDPR*, *LDB2*, *TAPT1*, *FBXL5*, *NKX3-2*, *PRMT3*, *NELL1*, *ANO5*, *SLC17A6*, *GAS2*, *SLC5A12*, *FIBIN*, *LGR4*, *BDNF*, *CCND1*, *FGF4*, *FGF3*, *SHANK2*, *NAT10*, *CAT*, *EHF*, *TRAF6*, *API5*, *SMOC1*, *MEIS2*, *ACTC1*, *GJD2*, *SSTR1*, *ACYP1*, *FOS*, *JDP2*, *TGFB3*, *EML5*, *PSMC1*, *CALM1*, *FBLN5*, *NDUFB1*, *VRK1*, *SIVA1*, *AKT1*, *JAG2*, *GCH1*, *CDKN3*, *SAV1*, *GNG2*, *PTGER2*, *BMP4*, *HIBCH*, *MSTN*, *FRZB*, *ACADL*, *MYL1*, *FN1*, *MLPH*, *AHR2*, *COL18A1*, *COL6A1*, *COL6A2*, *LOC124418108* (*BlSK1*) , *LSS*, *PCNT*, *ITGB2*, *STAT1*, *STAT4*, *MYO1B*, *NABP1*, *ANKRD44*, *SF3B1*, *MIR1603*, *HSPD1*, *CREB1*, *KLF7*, *ADAM23*, *GPR1*, *NBEAL1*, *IDH1*, *PIKFYVE*, *PPP1R1C*, *SLC25A12*, *HAT1*, *LRP2*, *CERS6*, *MYLK*, *CCDC14*, *ITGB5*, *PARP9*, *MARCO*, *RFWD2*, *RNPC3*, *S1PR1*, *TGFBR3*, *LEPROT*, *LEPR*, *FOXL2*, *AHSG*, *MIR15B*, *IL12A*, *RARRES1*, *LXN*, *PTX3*, *MIR1658*, *DHX36*, *IGSF10*, *PFN2*, *TSPAN3*, *FBXO22*, *IREB2*, *CRABP1*, *DNAJA4*, *ACSBG1*, *IDH3A*, *ANXA2*, *TRPM1*, *TJP1*, *THSD4*, *CYP19A1*, *NKD1*, *ZNF423*, *PHKB*, *DNAJA2*, *GPT2*, *URI1*, *ZNF536*, *ZDHHC7*, *IRF8*, *SLC7A5*, *BANP*, *CIDEC*, *ZC3H18*, *IL17C*, *CYBA*, *RPL13*, *MC1R*, *CDH1*, *SNTB2*, *NFAT5*, *NQO1*, *ZFHX3*, *TAT*, *CACNA1D*, *ARF4*, *FLNB*, *DNASE1L3*, *QARS*, *ARIH2*, *PDHB*, *ACOX2*, *THOC7*, *IRAK2*, *GHRL*, *RPL32*, *MBD4*, *RHO*, *TRH*, *TRPC7*, *LOC101749540*, *SMAD5*, *SH3RF2*, *STK32A*, *ACTB*, *MED9*, *USP7*, *MAPK8IP3*, *XBP1*, *LOC416924* (*THADA*) , *IGLL1*, *CHCHD10*, *MMP11*, *MIF*, *GSTT1L*, *GSTT1*, *GGT5*, *SLC5A1*, *MYH1D*, *MYH1F*, *MYH1A*, *MYH1B*, *SOCS3*, *AFMID*, *TK1*, *TMC6*, *GCGR*, *NPB*, *SIRT7*, *PYCR1*, *SPAG9*, *WFIKKN2*, *CACNA1G*, *ACSF2*, *CD300LG*, *FADS6*, *KCTD2*, *MIF4GD*, *P2RX1*, *IFT22*, *CAMKK1*, *C1QBP*, *SLC43A2*, *SERPINF2*, *SERPINF1*, *HIC1*, *MIR1666*, *MIR1696*, *FOXN1*, *ALDOC*, *MIR451*, *GIT1*, *SLC6A4*, *BLMH*, *TRPV2*, *WISP2*, *ADA*, *HNF4A*, *FITM2*, *EYA2*, *SNAI1*, *KIF1B*, *CITED4*, *GBP1*, *THEMIS2*, *NR0B2*, *PIM1*, *KIF21B*, *ELF3*, *ARL8A*, *PTPN7*, *PHLDA3*, *TNNI1*, *LAD1*, *DAD1*, *MIR6644-2*, *GOSR2*, *WNT3*, *MAP3K14*, *GH*, *CD79B*, *LOC771308*, *FTSJ3*, *BTN1A1*, *PIAS4*, *EEF2*, *MYO1F*, *MIR6615*, *ZAP70*, *GDF3*, *COMP*, *CRTC1*, *CRLF1*, *UBA52*, *ELL*, *GDF15*, *JUND*, *IFI30*, *INSR*, *USE1*, *MYO9B*, *TMEM38A*, *MIR6693*, *SMIM7*, *CHERP*, *CALR3*, *KLF2*, *AP1M1*, *RAB8A*, *PTPRS*, *MIR6666* |
| OMF | 121 | *TMEM168*, *IFRD1*, *DOCK4*, *IFNG*, *SLCO1A2*, *IAPP*, *SOX5*, *PNPLA3*, *NTF3*, *ANO2*, *FKBP4*, *SLC2A14*, *AICDA*, *MYH15*, *WNT3A*, *WNT9A*, *ITGA8*, *ACBD7*, *CROT*, *CTNND2*, *DAP*, *ROPN1L*, *GALNT1*, *INO80C*, *TTR*, *CSMD3*, *HADHA*, *HADHB*, *HPGDS*, *LEF1*, *HADH*, *CYP2U1*, *ACSL1*, *IRF2*, *SMOC1*, *SSTR1*, *VRK1*, *HIBCH*, *MSTN*, *FRZB*, *COL6A2*, *LOC124418108* (*BlSK1*) , *LSS*, *PCNT*, *ITGB2*, *STAT1*, *STAT4*, *MYO1B*, *NABP1*, *CREB1*, *KLF7*, *ADAM23*, *GPR1*, *MIR15B*, *IL12A*, *RARRES1*, *LXN*, *TRPM1*, *TJP1*, *SLC7A5*, *BANP*, *RPL13*, *MC1R*, *CDH1*, *SNTB2*, *NFAT5*, *NQO1*, *ARF4*, *FLNB*, *DNASE1L3*, *IRAK2*, *GHRL*, *RPL32*, *MBD4*, *RHO*, *TRH*, *SLC5A1*, *MYH1D*, *MYH1F*, *MYH1A*, *MYH1B*, *WISP2*, *ADA*, *HNF4A*, *FITM2*, *PIM1*, *KIF21B*, *ELF3*, *ARL8A*, *PTPN7*, *PHLDA3*, *TNNI1*, *LAD1*, *MAP3K14*, *PIAS4*, *EEF2*, *MYO1F*, *MIR6615*, *MMR1L3*, *MRC1*, *NDUFA4*, *GGPS1*, *COA6*, *SLC35F3*, *DISC1*, *TSNAX*, *EGLN1*, *EIF2B4*, *EVC2*, *SRSF5*, *ACTN1*, *RYR3*, *FMN1*, *UNC79*, *AMY2A*, *NTNG1*, *MITF*, *TULP1*, *FKBP5*, *CLPS*, *MAPK14* |
| RUW | 68 | *ACSL1*, *IRF2*, *IRAK2*, *GHRL*, *RPL32*, *MBD4*, *RHO*, *TRH*, *SLC5A1*, *COA6*, *SLC35F3*, *IL1RAPL1*, *PCDH9*, *TNFRSF11A*, *PRKDC*, *DECR1*, *CALB1*, *PXDN*, *TLR2A*, *TLR2B*, *SFRP2*, *DKK2*, *CCND1*, *FGF4*, *FGF3*, *SHANK2*, *FBLN5*, *SIVA1*, *AKT1*, *GCH1*, *CDKN3*, *FOXL2*, *MIR1658*, *DHX36*, *IGSF10*, *PFN2*, *P2RX1*, *IFT22*, *CAMKK1*, *C1QBP*, *SERPINF2*, *SERPINF1*, *HIC1*, *MIR1666*, *MIR1696*, *FOXN1*, *ALDOC*, *MYF6*, *MYF5*, *LRRIQ1*, *ALX1*, *KITLG*, *DUSP6*, *HNF4G*, *WWP1*, *MMP16*, *RIPK2*, *OSGIN2*, *GLP1R*, *BTBD9*, *MLK4*, *ENSGALG00000046723*, *FGB*, *FGA*, *SPOCK3*, *ANXA10*, *DDX60*, *CAV3* |
| WCR | 85 | *PRKDC*, *NTF3*, *ANO2*, *FKBP4*, *SLC2A14*, *AICDA*, *CTNND2*, *DAP*, *ROPN1L*, *CREB1*, *KLF7*, *RPL13*, *MYH1D*, *MYH1F*, *MYH1A*, *MYH1B*, *NDUFA4*, *RYR3*, *FMN1*, *OVST*, *RBMS3*, *CDH12*, *ANKH*, *CMBL*, *CCT5*, *SEMA5A*, *MTRR*, *FASTKD3*, *SRD5A1*, *EHBP1*, *PITX2*, *ELOVL6*, *EGF*, *PLA2G12A*, *CASP6*, *PDLIM5*, *BMPR1B*, *PCDH7*, *MEIS2*, *ACTC1*, *GJD2*, *ANKRD44*, *SF3B1*, *MIR1603*, *HSPD1*, *PARP9*, *MARCO*, *RFWD2*, *TGFBR3*, *NKD1*, *ZC3H18*, *IL17C*, *CYBA*, *CITED4*, *GBP1*, *THEMIS2*, *INSR*, *USE1*, *MYO9B*, *TMEM38A*, *MIR6693*, *SMIM7*, *CHERP*, *CALR3*, *KLF2*, *AP1M1*, *RAB8A*, *PTPRS*, *MIR6666*, *CRY1*, *TMEM263*, *NUAK1*, *SLC41A2*, *CHST11*, *HSP90B1*, *STAB2*, *IGF1*, *PMCH*, *ICA1*, *NXPH1*, *THBS1*, *AVEN*, *GREM1*, *CACYBP*, *RABGAP1L* |

^1^*n*, number of genes per breed
