# Supplementary material for: Selective footprints and genes relevant to cold adaptation and other phenotypic traits are unscrambled in the genomes of divergently selected chicken breeds
Source: J Anim Sci Biotechnol. 2023 Feb 24;14:35. doi: 10.1186/s40104-022-00813-0 (PMC9951459; doi:10.1186/s40104-022-00813-0)
Supplement: Supplementary file 11 — Additional file 11: Fig. S5. Venn diagrams representing distribution of 540 prioritized genes between the four studied breeds by phenotypic categories (A to L). [file 40104_2022_813_MOESM11_ESM.docx]

| **A** cold tolerance  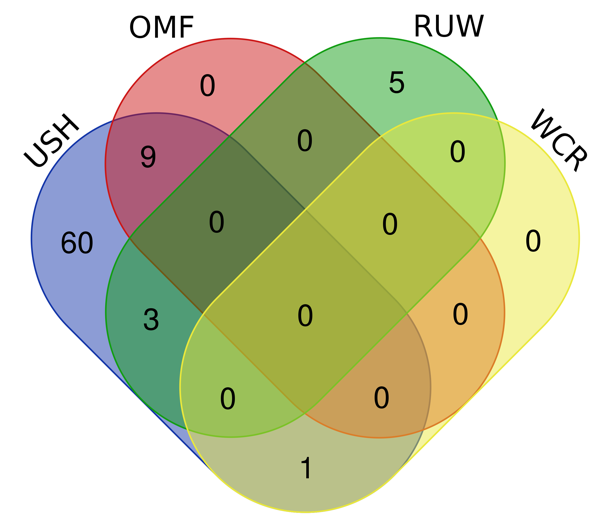 | **G** immunity  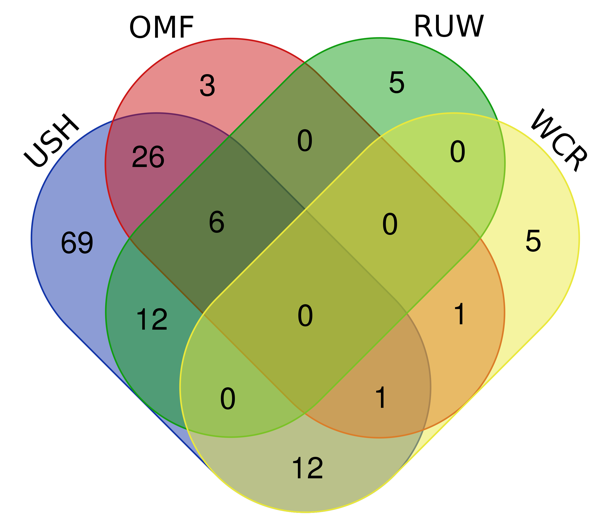 |
| --- | --- |
| **B** domestication  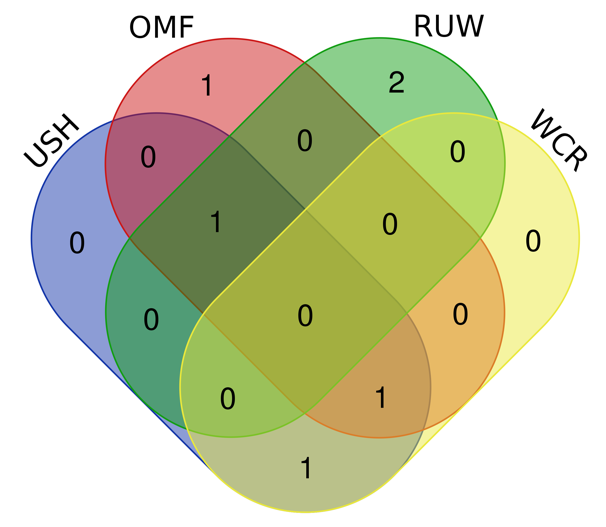 | **H** reproduction  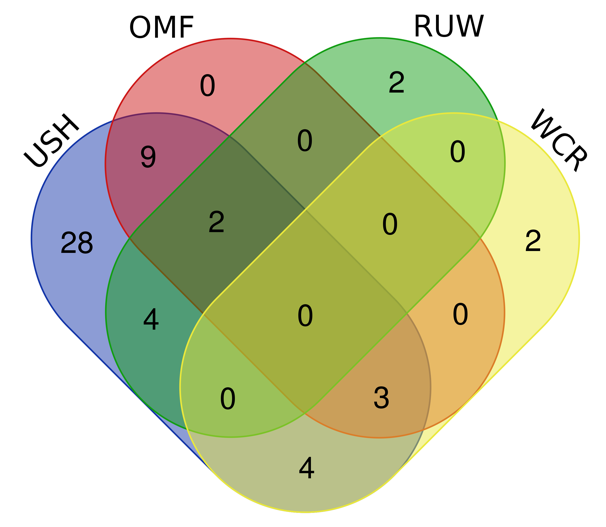 |
| **B** egg traits  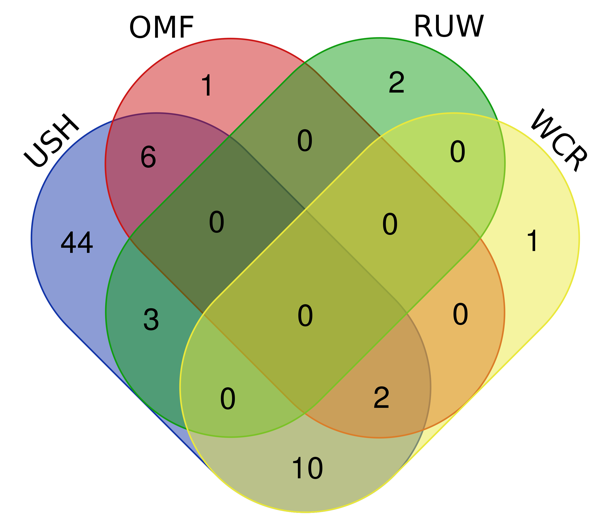 | **I** response to heat  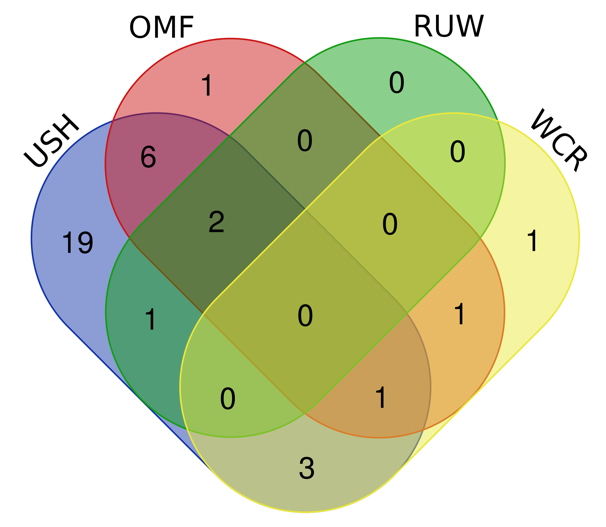 |

| **D** energy and feed intake  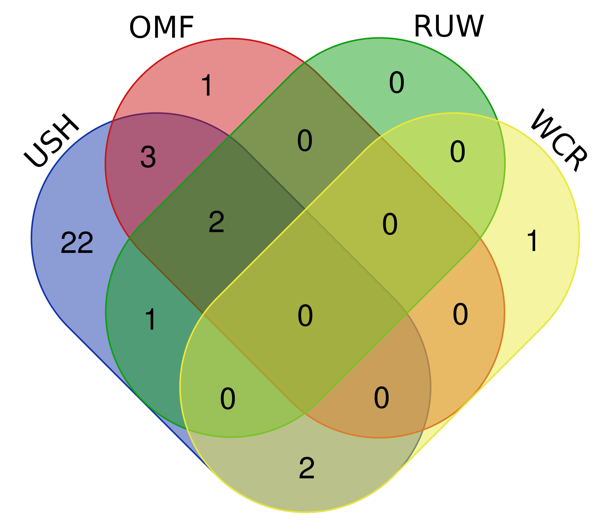 | **J** skin, feather, skin other appendages  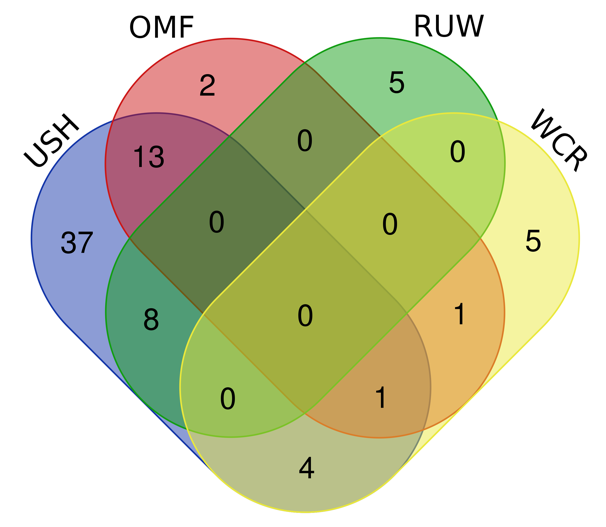 |
| --- | --- |
| **E** fat metabolism  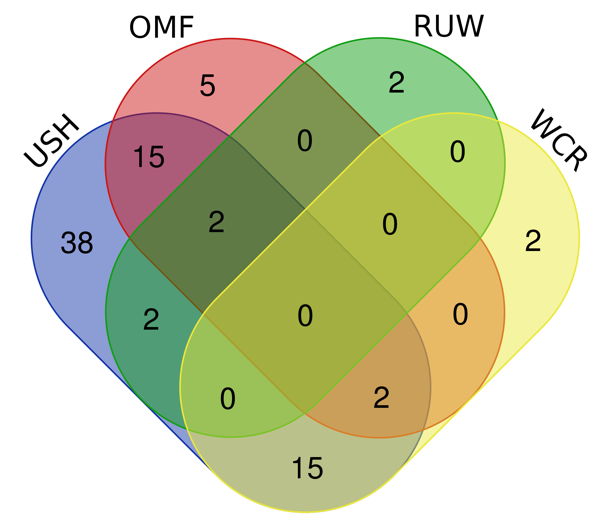 | **K** stress and adaptation  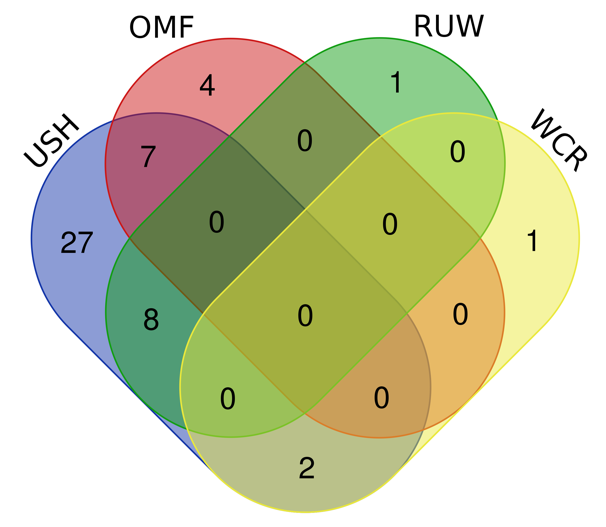 |
| **F** growth, meat, carcass  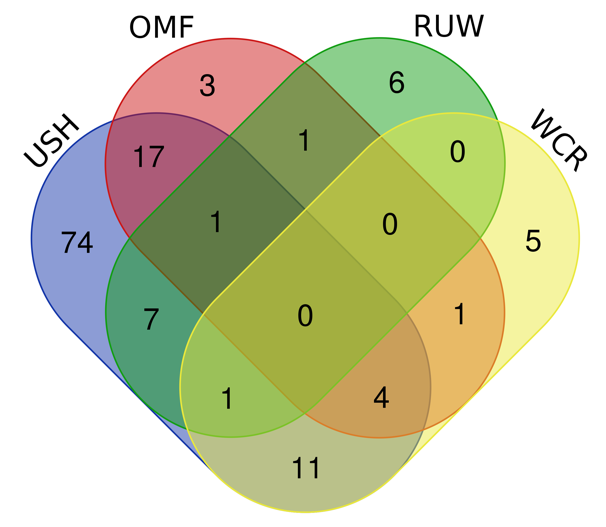 | **L** thermosensation  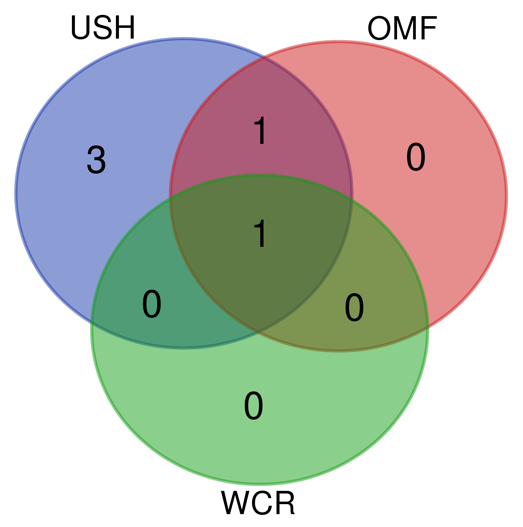 |

**Additional file 11: Fig. S5** Venn diagrams representing distribution of 540 prioritized genes between the four studied breeds by phenotypic categories (**A** to **L**). Breeds: OMF, Orloff Mille Fleur; RUW, Russian White; USH, Ushanka; WCR, White Cornish
