# Supplementary material for: Selective footprints and genes relevant to cold adaptation and other phenotypic traits are unscrambled in the genomes of divergently selected chicken breeds
Source: J Anim Sci Biotechnol. 2023 Feb 24;14:35. doi: 10.1186/s40104-022-00813-0 (PMC9951459; doi:10.1186/s40104-022-00813-0)
Supplement: Supplementary file 12 — Additional file 12: Table S10. Phenotypic traits linked to known QTLs and associated genes within selective sweep regions identified in the genomes of the four chicken breeds. [file 40104_2022_813_MOESM12_ESM.docx]

**Additional file 12: Table S10** Phenotypic traits linked to known QTLs and associated genes within selective sweep regions identified in the genomes of the four chicken breeds

| **Trait class: Trait name** | **Chromosome** | **Regions, Mb^1^** | **QTL ID^2^** | **Associated genes^3^** | **Breed^4^** |
| --- | --- | --- | --- | --- | --- |
| *Potentially related to thermoregulation* | | | | | |
| Exterior: Feather density | 1 | 163.9..167.3 | 194021 | n/a^5^ | USH |
|  | 4 | 69.3..77.5 | 194005, 194011, 194006 | n/a^5^ | USH |
| Health: Body temperature | 2 | 75.0..80.0 | 9112 | n/a^5^ | USH, WCR, OMF |
|  |  | 105.0..111.0 | 30853 | n/a^5^ | USH |
|  | 15 | 7.5..9.5 | 95399, 95398 | n/a^5^ | USH |
| *Others* | | | | | |
| Exterior: Aggressive behavior | 1 | 53.5..55.6 | 119901, 119902 | ***CRY1***, ***TMEM263*** | WCR |
|  |  |  | 119893, 119903 | ***CHST11*** | WCR, USH |
| Exterior: Feather pecking | 4 | 37.2..46.0 | 15640 | *GALNT7* | USH |
|  | 5 | 35.0..35.7 | 15659 | *NPAS3* | USH |
|  | 7 | 27.1..29.3 | 15669 | *SNX4* | USH |
|  |  |  | 15670 | *PARP14L* | USH, WCR |
| Exterior: Feather pigmentation | 1 | 53.5..55.6 | 137117, 137118 | ***NUAK1*** | WCR |
|  | 7 | 17.1..19.0 | 179163 | *PPP1R19L* | USH |
|  | 15 | 7.5..9.5 | 179179 | *TTC28* | USH |
| Health: Antibody titer to IBV | 5 | 50.9..52.6 | 24351 | *CEP170B* | USH |
| Health: Antibody titer to KLH antigen | 4 | 37.2..46.0 | 37497 | *BMP3* | USH |
| Health: Avian influenza susceptibility | 7 | 27.1..29.3 | 193661 | *DPP10* | USH |
| Health: Immunoglobulin Y level | 1 | 114.0..119.9 | 37945 | ***EIF2S3*** | USH |
| Health: Pullorum disease susceptibility | 5 | 56.5..59.8 | 170752, 170756, 170757 | *FERMT2*, *FANCM* | USH |
| Physiology: Heterophil/lymphocyte ratio | 7 | 11.6..12.7 | 223383 | *PLEKHM3* | OMF, USH, WCR |
|  | 11 | 17.4..19.9 | 171490 | ***BANP*** | USH, OMF |
|  | 12 | 12.0..15.9 | 171491 | *PTPRG* | USH |
| Physiology: Heterophil number | 11 | 17.4..19.9 | 171495 | ***BANP*** | USH, OMF |
| Physiology: Lymphocyte number | 12 | 12.0..15.9 | 171500 | *PTPRG* | USH |
| Physiology: Monocyte number | 5 | 44.3..45.5 | 171505 | *CPSF2* | USH |
| Production: Abdominal fat weight | 5 | 56.5..59.8 | 95359 | *CGRRF1* | USH, RUW |
|  | 28 | 1.4..2.1 | 24371 | *ACSBG2* | USH |
|  |  |  | 24370 |  | USH, OMF |
| Production: Albumen height | 4 | 69.3..77.5 | 24916 | ***NCAPG*** | USH |
| Production: Average daily gain | 4 | 69.3..77.5 | 160797–160800, 160802, 160803, 160807–160812, 160818, 160821 | ***SLIT2***, ***LCORL***, ***LAP3***, ***LDB2***, ***TAPT1***, *CD38* | USH |
| Production: Body depth | 9 | 21.4..24.1 | 161555 | ***MIR1666*** | USH, RUW, WCR |
| Production: Body slope length | 9 | 21.4..24.1 | 161557, 161558 | ***MIR1658*** | USH, RUW, WCR |
|  | 19 | 5.3..6.8 | 56217–56219 | ***MIR1666*** | USH, RUW |
|  | 27 | 2.2..4.2 | 221839 | *WNT9B* | USH |
| Production: Body weight | 1 | 61.9..66.1 | 167282 | ***SOX5*** | OMF |
|  |  | 188.7..195.7 | 24329–24333, 160881–160883, 19260 | ***MIR1657***, ***ME3***, ***WNT11*** | USH |
|  | 2 | 2.6..4.0 | 19279, 19319 | ***WNT3A***, ***WNT9A*** | OMF |
|  | 4 | 69.3..77.5 | 238219–238226, 238228, 238229, 193593, 65702, 65707, 64510, 65706, 64511, 65700, 65696, 65703, 62154 | ***CCKAR***, ***RBPJ***, ***KCNIP4***, ***FAM184B***, ***FBXL5*** | USH |
|  | 7 | 11.6..12.7 | 256985 | ***ADAM23*** | OMF, USH |
|  | 9 | 21.4..24.1 | 161551–161554 | ***MIR1658*** | USH, RUW, WCR |
|  | 12 | 12.0..15.9 | 167275, 167279 | *PTPRG* | USH |
|  |  | 19.6..20.3 | 193692 | ***GHRL*** | RUW, OMF, USH |
|  | 19 | 5.3..6.8 | 56205 | ***MIR1666*** | USH, RUW |
| Production: Breast bone crest length | 19 | 5.3..6.8 | 56214–56216 | ***MIR1666*** | USH, RUW |
|  | 27 | 2.2..4.2 | 221829 | *WNT9B* | USH |
| Production: Breast muscle percentage | 2 | 2.6..4.0 | 19278, 19275 | ***WNT3A*** | OMF |
|  | 4 | 37.2..46.0 | 19220 | *LEF1* | USH, OMF |
|  | 5 | 17.7..21.8 | 19211 | ***CCND1*** | USH |
| Production: Breast muscle weight | 2 | 2.6..4.0 | 19292, 19283, 19317 | ***WNT3A***, ***WNT9A*** | OMF |
|  | 19 | 5.3..6.8 | 56202 | ***MIR1666*** | USH, RUW |
| Production: Bursa of Fabricius weight | 10 | 3.5..6.8 | 101238 | *LRRC49* | USH |
| Production: Carcass weight | 2 | 2.6..4.0 | 19280, 19314, 19308, 19323 | ***WNT3A***, ***WNT9A*** | OMF |
|  | 4 | 69.3..77.5 | 24404, 24395 | ***LDB2*** | USH |
|  | 19 | 5.3..6.8 | 56201, 56200 | ***MIR1666*** | USH, RUW |
| Production: Chest width | 9 | 21.4..24.1 | 161556 | ***MIR1658*** | USH, RUW, WCR |
|  | 19 | 5.3..6.8 | 56211–56213 | ***MIR1666*** | USH, RUW |
| Production: Cingular fat width | 15 | 7.5..9.5 | 24376 | *DDTL* | USH |
| Production: Claw percentage | 4 | 69.3..77.5 | 221895, 221911, 221871, 221943, 221921 | *CCDC149*, ***KCNIP4***, ***LAP3***, *PROM1* | USH |
| Production: Claw weight | 4 | 69.3..77.5 | 221975, 222064, 221960, 222090, 222051, 222030, 221959, 222019, 222065, 221961–221963, 222039, 222072, 222091, 222084 | ***TBC1D1***, *NWD2*, *SLC34A2*, *CCDC149*, ***ADGRA3***, ***KCNIP4***, ***PACRGL***, ***SLIT2***, ***TAPT1***, *PROM1*, ***FBXL5*** | USH |
| Production: Drumstick and thigh muscle percentage | 1 | 188.7..195.7 | 19262 | ***WNT11*** | USH |
|  | 2 | 2.6..4.0 | 19321 | ***WNT9A*** | OMF |
| Production: Drumstick and thigh muscle weight | 2 | 2.6..4.0 | 19291, 19282, 19316 | ***WNT3A***, ***WNT9A*** | OMF |
|  | 19 | 5.3..6.8 | 56204 | ***MIR1666*** | USH, RUW |
| Production: Drumstick and thigh percentage | 1 | 188.7..195.7 | 19261, 19264 | ***WNT11*** | USH |
|  | 2 | 2.6..4.0 | 19276, 19320 | ***WNT3A***, ***WNT9A*** | OMF |
| Production: Drumstick and thigh weight | 2 | 2.6..4.0 | 19290, 19288, 19281, 19315 | ***WNT3A***, ***WNT9A*** | OMF |
|  | 4 | 37.2..46.0 | 19219 | *LEF1* | USH, OMF |
|  |  | 69.3..77.5 | 222162, 222159, 222168 | ***ADGRA3***, ***PACRGL*** | USH |
|  | 19 | 5.3..6.8 | 56203 | ***MIR1666*** | USH, RUW |
| Production: Egg number | 1 | 114.0..119.9 | 177382–177390 | ***POLA1*** | USH |
|  |  |  | 177395 | ***ACOT9*** | USH |
| Production: Eggshell thickness | 26 | 0.1..1.0 | 193611, 193612 | ***ARL8A*** | OMF, USH |
| Production: Eggshell weight | 2 | 81.0..84.3 | 16739 | ***GALNT1*** | OMF, USH |
|  | 11 | 6.2..9.3 | 16741 | ***ZNF536*** | USH |
| Production: Feed conversion ratio | 12 | 19.6..20.3 | 193694 | ***GHRL*** | RUW, OMF, USH |
| Production: Feed intake | 4 | 37.2..46.0 | 195035–195049 | *TET2* | USH |
|  |  | 57.7..61.2 | 194985 | ***BMPR1B*** | USH |
| Production: Femur area | 4 | 69.3..77.5 | 193680 | ***NCAPG*** | USH |
| Production: Femur length | 4 | 69.3..77.5 | 193682 | ***NCAPG*** | USH |
| Production: Gizzard weight | 4 | 69.3..77.5 | 170517, 170521, 170525 | ***NCAPG*** | USH |
| Production: Growth | 12 | 19.6..20.3 | 193693 | ***GHRL*** | RUW, OMF, USH |
| Production: Heart weight | 4 | 69.3..77.5 | 170514, 170518, 170522 | ***NCAPG*** | USH |
| Production: Intramuscular fat percentage | 2 | 2.6..4.0 | 62100 | ***SETD2*** | USH |
|  |  | 129.5..134.4 | 62102 | ***ZFPM2*** | USH |
| Production: Keel length | 1 | 188.7..195.7 | 24334 | ***MIR1657*** | USH |
|  | 7 | 0.1..4.6 | 256989 | *ITGAV* | USH, OMF |
| Production: Linoleic acid content | 4 | 57.7..61.2 | 193108, 193107 | ***ELOVL6*** | WCR, USH |
| Production: Liver weight | 4 | 69.3..77.5 | 170515, 170523 | ***NCAPG*** | USH |
| Production: Muscle dry matter content | 2 | 75.0..80.0 | 24477 | *FAM105A* | USH |
|  |  | 129.5..134.4 | 24453 | *ANGPT1* | USH |
| Production: Ovary weight | 28 | 1.4..2.1 | 62024, 62033, 62044 | *ACER1*, *ANP32B*, ***MYO1F*** | USH, OMF |
|  |  |  | 62043, 62026 | *ADAMTS10* | USH |
| Production: Pelvis breadth | 1 | 188.7..197.7 | 24335 | ***MIR1657*** | USH |
|  | 9 | 21.4..24.1 | 161559 | ***MIR1658*** | USH, RUW, WCR |
| Production: Proventriculus weight | 4 | 69.3..77.5 | 170516, 170519, 170524 | ***NCAPG*** | USH |
| Production: Residual feed intake | 12 | 16.5..18.6 | 107838, 107839, 107841, 108152–108155, 107843, 107844, 107849–107851 | *RYBP*, *PDZRN3*, *CHL1* | USH, OMF |
|  | 23 | 1.3..2.0 | 108156 | *ZMPSTE24* | USH |
|  | 27 | 2.2..4.2 | 194976, 195057–195073, 194977, 194981, 194979, 194991, 194992 | *GTSF1*, *WNT9B*, ***WNT3A***, ***WNT3***, *NSF* | USH |
| Production: Shank circumference | 1 | 188.7..195.7 | 19263, 19265 | ***WNT11*** | USH |
|  | 2 | 2.6..4.0 | 19295, 19293, 19287, 19322, 19311, 19325, 19313 | ***WNT3A***, ***WNT9A*** | OMF |
|  | 4 | 37.2..46.0 | 19221 | *LEF1* | USH, OMF |
|  | 5 | 17.7..21.8 | 19212, 19213 | ***CCND1*** | USH |
|  | 19 | 5.3..6.8 | 56208–56210 | ***MIR1666*** | USH, RUW |
| Production: Shank length | 2 | 2.6..4.0 | 19289, 19286, 19318, 19310, 19312 | ***WNT3A***, ***WNT9A*** | OMF |
|  | 4 | 69.3..77.5 | 222237, 193683 | ***PACRGL***, ***NCAPG*** | USH |
|  | 19 | 5.3..6.8 | 56206, 56207 | ***MIR1666*** | USH, RUW |
| Production: Spleen percentage | 3 | 104.3..105.5 | 222243 | *ASXL2* | OMF |
| Production: Subcutaneous fat thickness | 1 | 188.7..195.7 | 24328 | ***MIR1657*** | USH |
|  | 4 | 57.7..61.2 | 24375 | ***ELOVL6*** | WCR, USH |
| Production: Tibia length | 4 | 69.3..77.5 | 193678, 193681 | ***LCORL***, ***NCAPG*** | USH |
| Production: Tibia weight | 4 | 69.3..77.5 | 193679, 193684 | ***LCORL***, ***NCAPG*** | USH |
| Production: Wing percentage | 2 | 2.6..4.0 | 19277, 19285, 19309, 19324 | ***WNT3A***, ***WNT9A*** | OMF |
| Production: Wing weight | 2 | 2.6..4.0 | 19294, 19284 | ***WNT3A*** | OMF |

^1^ Genomic selective sweep regions (in Mb) identified in present study. ^2^ As shown in Chicken QTLdb [47]. ^3^ Associated genes per category include candidate genes and prioritized candidate genes (in bold) identified in this study. ^4^ Breeds that harbor loci/genes in this study: OMF, Orloff Mille Fleur; RUW, Russian White; USH, Ushanka; WCR, White Cornish. ^5^ n/a, not available (meaning QTLs for which association data type is indicated in Chicken QTLdb but the respective genes are not reported).
